# Discovery of new pyridine-quinoline hybrids as competitive and non-competitive PIM-1 kinase inhibitors with apoptosis induction and caspase 3/7 activation capabilities

**DOI:** 10.1080/14756366.2022.2152810

**Published:** 2023-01-11

**Authors:** Mostafa M. M. El-Miligy, Marwa E. Abdelaziz, Salwa M. Fahmy, Tamer M. Ibrahim, Marwa M. Abu-Serie, Mona A. Mahran, Aly A. Hazzaa

**Affiliations:** a Pharmaceutical Chemistry Department, Faculty of Pharmacy, Alexandria University, Alexandria, Egypt; b Department of Pharmaceutical Chemistry, Faculty of Pharmacy, Kafrelsheikh University, Kafrelsheikh, Egypt; c Medical Biotechnology Department, Genetic Engineering and Biotechnology Research Institute (GEBRI, City of Scientific Research and Technological Applications (SRTA-City), Alexandria, Egypt)

**Keywords:** Pyridine, quinoline, apoptosis induction, caspase 3/7 activation, PIM-1 kinase inhibitors

## Abstract

New quinoline-pyridine hybrids were designed and synthesised as PIM-1/2 kinase inhibitors. Compounds **5b**, **5c**, **6e, 13a**, **13c,** and **14a** showed *in-vitro* low cytotoxicity against normal human lung fibroblast Wi-38 cell line and potent *in-vitro* anticancer activity against myeloid leukaemia (NFS-60), liver (HepG-2), prostate (PC-3), and colon (Caco-2) cancer cell lines. In addition, **6e, 13a,** and **13c** significantly induced apoptosis with percentage more than 66%. Moreover, **6e, 13a,** and **13c** significantly induced caspase 3/7 activation in HepG-2 cell line. Furthermore, **5c, 6e,** and **14a** showed potent *in-vitro* PIM-1 kinase inhibitory activity. While, **5b** showed potent *in-vitro* PIM-2 kinase inhibitory activity. Kinetic studies using Lineweaver–Burk double-reciprocal plot indicated that **5b**, **5c**, **6e,** and **14a** behaved as competitive inhibitors while **13a** behaved as both competitive and non-competitive inhibitor of PIM-1 kinase enzyme. Molecular docking studies indicated that, *in-silico* affinity came in coherence with the observed *in-vitro* inhibitory activities against PIM-1/2 kinases.

## Introduction

**Figure 1.**
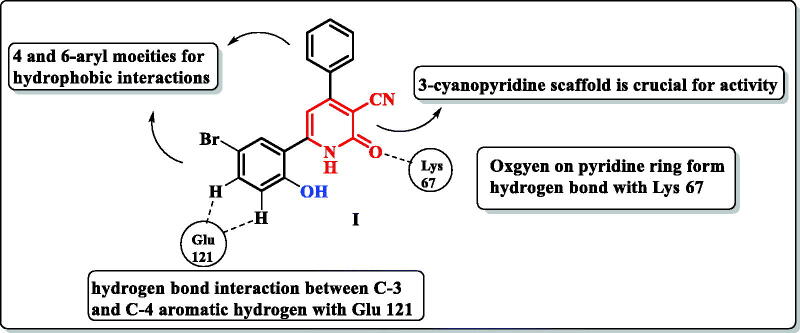
A schematic representation of pyridone derivative I complexed with PIM-1 kinase. Dashed lines indicated hydrogen bonds.

Human Proviral Integration Moloney (PIM) kinases are a family of serine/threonine kinases includes PIM-1, PIM-2, and PIM-3 isoforms. The PIM-1 kinase has direct role in tumorigenesis in various haematological malignancies^1^, such as leukaemia and lymphoma^2–5^. Also, PIM-1 kinase is overexpressed in several solid tumours such as prostate^6^, colon^7^, hepatic^8^, and breast cancers^9^. In addition, it is involved in many biological processes such as cell cycle, cell proliferation, apoptosis, and drug resistance^10^. PIM-1 kinase has unusual adenosine triphosphate (ATP) binding pocket having proline 123 in the hinge region which is specific to PIM-1 kinase over other serine/threonine kinases^11^. Therefore, ATP could not bind to the hinge region by a second hydrogen bond as other protein kinases^12^^,^^13^. In addition, most PIM-1 kinase inhibitors are ATP-competitive inhibitors^14^. PIM-1 kinase competitive inhibitors are classified into non-ATP mimetics and ATP-mimetics. ATP-mimetics bind directly to the hinge region via Glu121 while non-ATP mimetics bind to Lys67^15^^,^^16^. PIM-1 kinase inhibition is an attractive target to overcome PIM-1 kinase induced chemotherapy resistance caused by induction of hypoxia in tumour cells^17^. X-ray crystallographic study of the lead scaffold **I** showed on interaction to ATP-binding site of PIM-1 kinase through prominent Hydrogen bond interaction of the carbonyl group on the pyridone ring with the Lys67. Another weak H-bond was observed with the hinge region between the main chain carbonyl of Glu121 and an aromatic hydrogen (C-3 position and C-4-position) on 6-phenyl moiety. Besides, the hydrophobic interactions with aryl moieties at 4 and 6 positions^18^ (Figure 1).

**Figure 2.**
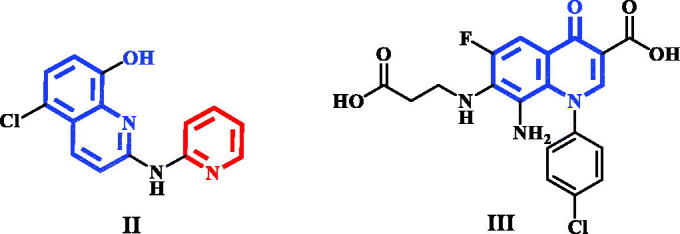
Quinoline and quinolone having PIM-1 kinase inhibitory activity.

Pyridine-quinoline hybrid structure (compound **II**) showed potent inhibition of PIM-1 kinase, increase anticancer activity against human prostatic PC-3 cancer cell line as well as induction apoptosis^19^. In addition, structure based drug design of PIM-1 kinase inhibitors revealed that electron deficient quinolone core, as in compound **III**, was able to form strong interactions with the hinge region of PIM-1 kinase^20^ (Figure 2).

**Figure 3.**
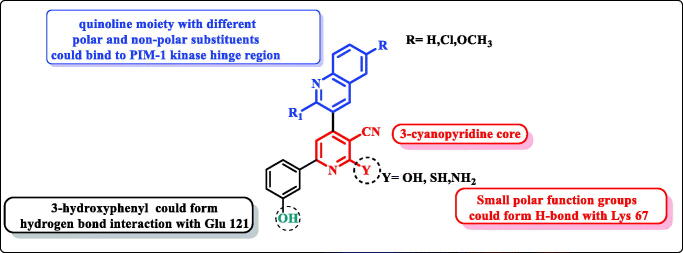
Design of pyridine-quinoline hybrids.

In continuation of our previously published work^21^, the structure-based design strategy was rationalised via tethering the pyridine moiety with the quinoline moiety with keeping the common respective pharmacophoric features of the lead scaffold **I** (Figure 1) to improve the PIM kinase inhibitory activity. It must be pointed out that, the six-position of pyridine ring was selected to be substituted with the bioactive pharmacophore 3-hydroxyphenyl group which was reported to enhance the anticancer activity through hydrogen bond interactions. In addition, the designed scaffold was modified at numerous positions, where the pyridine ring was substituted with either oxygen or its isostere sulphur or its isostere amino group at position 2. Moreover, pyridine was also hybridised with electron deficient quinolone or quinoline substituted at position 2 with either piperidine or morpholine and substituted at six-position with either hydrophilic or hydrophobic groups to impart variety of enzyme interactions hoping to enhance binding affinity hence potency (Figure 3).

## Experimental

### Chemistry

#### General

All chemicals and solvents were purchased from commercial suppliers. Melting points were determined in open-glass capillaries using a Griffin melting point apparatus and were uncorrected. The progress of the reactions was monitored by thin-layer chromatography (TLC) on commercially available precoated silica gel aluminium-backed plates and the spots were visualised by exposure to iodine vapours or UV-lamp at λ 254 nm for few seconds. Infra-red spectra (IR) were recorded, for KBr discs, on a PerkinElmer RXIFT-IR. Nuclear magnetic resonance spectra, ^1^H-NMR and ^13^C-NMR, were recorded on Bruker spectrometer (400 MHz) using DMSO-d6 as solvent using deuterated dimethyl sulfoxide as solvents. The data were reported as chemical shifts or δ values (ppm) relative to tetramethyl silane (TMS) as internal standard. Signals were indicated by the following abbreviations: s = singlet, d = doublet, t = triplet, q = quartette and m = multiplet, dd = doublet of doublet and br. = broad. Electron impact mass spectra (EIMS) were run on a gas chromatograph/mass spectrophotometer Shimadzu GCMS/QP-2010 plus (70 eV). Relative intensity % corresponding to the most characteristic fragments is recorded. Elemental microanalyses were performed at the microanalytical unit, the Regional Centre of Microbiology and Biotechnology, Al-Azhar University, Egypt.

Compounds **2a–c**, **3a–c**, and **4a–c** were prepared as reported^22–24^.

### General experimental procedure for the synthesis of 4-(6-substituted-2-morpholino or (piperidin-1-yl)quinolin-3-yl)-6-(3-hydroxyphenyl)-2-oxo-1,2-dihydropyridine-3-carbonitrile; (5a–f)

A mixture of 3-hydroxyacetophenone (1 mmol), substituted aldehyde **4a–f** (1 mmol), ethyl cyanoacetate (1 mmol), and ammonium acetate (8 mmol) in 5 ml absolute ethanol was heated under reflux for 4 h during which the product was separated out. Then, the reaction mixture was cooled, and the yellow precipitate was filtered, washed with cold ethanol, air dried, and recrystallized from ethanol/DMF (9:1) mixture to give pure oxo-cyanopyridine derivatives.

#### 6-(3-Hydroxyphenyl)-4-(morpholin-4-yl-quinolin-3-yl)-2-oxo-1,2-dihydropyridine-3-carbonitrile (5a)

Yield, 40%; m.*p* > 300 °C; IR (KBr, v cm^−1^): 3400 (OH), 3218 (NH), 2200 (C≡N), 1649 (C=O), 1596 (C=N). ^1^H-NMR (400 MHz, DMSO-d_6_, δ ppm): 3.20–3.30 (m, 4H, morpholine-C_2,6_-H); 3.63 (t, *J* = 4,4.8 Hz, 4H, morpholine-C_3,5_-H); 6.97–6.99 (m, 2H, phenyl-C_4_-H and pyridine-C_5_-H); 7.25 (s, 1H, phenyl-C_2_-H); 7.31–7.37 (m, 2H, phenyl-C_5,6_-H); 7.45 (t, *J* = 7.2,7.6 Hz, 1H, quinoline- C_7_-H); 7.73 (t, *J* = 7.2, 8 Hz, 1H, quinoloine-C_6_-H); 7.80 (d, *J* = 8.4 Hz, 1H, quinoline-C_5_-H); 7.91 (d, *J* = 8 Hz, 1H, quinoline-C_8_-H); 8.38 (s, 1H, quinoline-C_4_-H); 9.87 (s, 1H, OH, D_2_O-exchangeable); 12.70 (brs, 1H, NH, D_2_O-exchangeable). Elemental analysis Calcd for C_25_H_20_N_4_O_3_ (424.46): C 70.74; H 4.75; N 13.20. Found: C 71.02, H 4.89, N 13.43

#### 4-(6-Chloro-2-morpholin-4-yl-quinolin-3-yl)-6-(3-hydroxyphenyl)-2-oxo-1,2-dihydropyridine-3-carbonitrile (5b)

Yield, 40%; m.*p* > 300 °C; IR (KBr, v cm^−1^): 3400 (OH), 3233 (NH), 2220 (C≡N), 1649 (C=O), 1592 (C=N). ^1^H-NMR (400 MHz, DMSO-d_6_, δ ppm): 3.20–3.30 (m, 4H, morpholine-C_2,6_-H); 3.60–3.70 (m, 4H, morpholine-C_3,5_-H); 6.90 (brs, 1H, pyridine-C_5_-H); 6.98 (d, *J* = 6.8 Hz, 1H, phenyl-C_4_-H); 7.23 (s, 1H, phenyl-C_2_-H); 7.31–7.37 (m, 2H, phenyl-C_5,6_-H); 7.72 (d, *J* = 8.8 Hz, 1H, quinoline-C_7_-H); 7.80 (d, *J* = 8.8 Hz, 1H, quinoline-C_8_-H); 8.02 (s, 1H, quinoline-C_5_-H); 8.37 (s, 1H, quinoline-C_4_-H); 9.91 (s, 1H, OH, D_2_O-exchangeable); 12.83 (brs, 1H, NH, D_2_O-exchangeable). EI-MS m/z (% relative abundance): 460 [M^+•^+2] (66.47); 458 [M^+•^] (100); 400(59.39); 402 (53.11). Elemental analysis Calcd for C_25_H_19_ClN_4_O_3_ (458.90): C 65.43; H 4.17; N 12.21. Found: C 65.27, H 4.28, N 12.47.

#### 6-(3-Hydroxyphenyl)-4-(6-methoxy-2-morpholin-4-yl-quinolin-3-yl)-2-oxo-1,2-dihydropyridine-3-carbonitrile (5c)

Yield, 40%; m.*p* > 300 °C; IR (KBr, v cm^−1^): 3400 (OH), 3237 (NH), 2219 (C≡N), 1647 (C=O), 1596 (C=N). ^1^H-NMR (400 MHz, DMSO-d_6_, δ ppm): 3.15–3.22 (m, 4H, morpholine-C_2,6_-H); 3.57–3.62 (m, 4H, morpholine-C_3,5_-H); 3.87 (s, 3H, OCH_3_); 6.93 (brs, 1H, pyridine-C_5_-H); 6.98 (d, *J* = 6.8 Hz, 1H, phenyl-C_4_-H); 7.25 (s, 1H, phenyl-C_2_-H); 7.31 (d, *J* = 8 Hz, 1H, quinoline-C_7_-H); 7.34 (s, 1H, quinoline-C_5_-H); 7.37 (dist. t, *J* = 8 Hz, phenyl-C_5_-H); 7.40 (d, *J* = 4 Hz, 1H, phenyl-C_6_-H); 7.74 (d, *J* = 8.8 Hz, 1H, quinoline-C_8_-H); 8.29 (s, 1H, quinoline-C_4_-H); 9.86 (s, 1H, OH, D_2_O-exchangeable); 12.71 (brs, 1H, NH, D_2_O-exchangeable). ^13^C-NMR (100 MHz, DMSO-d_6_δ ppm): 49.95 (morpoline-C_2,6_); 55.97 (OCH_3_); 66.41 (morpholine-C_3,5_); 104.96 (pyridine-C_5_); 107.02 (quinoline-C_5_); 114.73 (phenyl-C_2_); 116.60 (CN); 118.75 (phenyl-C_4_); 123.13 (phenyl-C_6_); 123.30 (pyridine-C_3_); 124.00 (quinoline-C_3_); 125.26 (quinoline-C_7_); 128.87 (quinoline-C_4a_); 130.64 (quinoline-C_8_); 139.06 (phenyl-C_5_); 139.13 (quinoline-C_4_); 141.64 (phenyl-C_1_); 142.98 (quinoline-C_8a_); 152.64 (quinoline-C_6_); 155.92 (phenyl-C_3_); 156.53 (C=O); 158.22 (pyridine-C_6_); 162.92 (pyridine-C_4_); 163.22 (quinoline-C_2_);. Elemental analysis Calcd for C_26_H_22_N_4_O_4_ (454.49): C 68.71; H 4.88; N 12.33. Found: C 68.94, H 5.12, N 12.59.

#### 6-(3-Hydroxyphenyl)-2-oxo-4-(2-(piperidin-1-yl)quinoline-3-yl)-1,2-dihydropyridine-3-carbonitrile (5d)

Yield, 40%; m.p 244–246 °C; IR (KBr, v cm^−1^): 3519 (OH), 3400 (NH), 2225 (C≡N), 1629 (C=O), 1595 (C=N). ^1^H-NMR (400 MHz, DMSO-d_6_, δ ppm): 1.53 (s, 6H, piperidine-C_3,4,5_-H); 3.23 (s, 4H, piperidine-C_2,4_-H); 6.94 (brs, 1H, pyridine-C_5_-H); 6.98 (d, *J* = 6.8 Hz, 1H, phenyl-C_4_-H); 7.25 (s, 1H, phenyl-C_2_-H); 7.31 (d, *J* = 7.6 Hz, 1H, phenyl-C_6_-H); 7.35 (dist. t, *J* = 7.6, 8 Hz, 1H, phenyl-C_5_-H); 7.41 (t, *J* = 7.2,7.6 Hz, 1H, quinoline-C_6_-H) 7.69 (t, *J* = 7.2, 8 Hz, 1H, quinoline-C_7_-H); 7.76 (d, *J* = 8.4 Hz, 1H, quinoline-C_8_-H); 7.87 (d, *J* = 8 Hz, 1H, quinoline-C_5_-H); 8.31 (s, 1H, quinoline-C_4_-H); 9.87 (s, 1H, OH, D_2_O-exchangeable); 12.76 (brs, 1H, NH, D_2_O-exchangeable). ^13^C-NMR (100 MHz, DMSO-d_6_δ ppm): 24.49 (piperdine-C_4_); 25.54 (piperdine-C_3,5_); 50.37 (piperdine-C_2,6_); 114.74 (pyridine-C_5_); 116.52 (phenyl-C_2_); 116.55 (CN); 116.57 (phenyl-C_4_); 118.75 (phenyl-C_6_); 123.86 (quinoline-C_3_); 124.09 (pyridine-C_3_); 124.10 (quinoline-C_4a_); 124.64 (quinoline-C_6_); 127.17 (quinoline-C_8_); 128.65 (quinoline-C_5_); 130.69 (phenyl-C_5_); 131.32 (quinoline-C_7_); 139.87 (quinoline-C_4_); 147.47 (phenyl-C_1_); 147.48 (quinoline-C_8a_); 156.97 (phenyl-C_3_); 158.20 (C=O); 158.23 (pyridine-C_4_); 158.24 (pyridine-C_6_); 158.27 (quinoline-C_2_). Elemental analysis Calcd for C_26_H_22_N_4_O_2_ (422.49): C 73.92; H 5.25; N 13.26. Found: C 74.19, H 5.39, N 13.53.

#### 4-(6-Chloro-2-(piperidin-1-yl)quinoline-3-yl)-6-(3-hydroxyphenyl)-2-oxo-1,2-dihydropyridine-3-carbonitrile (5e)

Yield, 40%; m.*p* > 300 °C; IR (KBr, v cm^−1^): 3454 (OH), 3210 (NH), 2224 (C≡N), 1634 (C=O), 1594 (C=N). ^1^H-NMR (400 MHz, DMSO-d_6_, δ ppm): 1.50 (s, 6H, piperidine-C_3,4,5_-H); 3.23 (s, 4H, piperidine-C_2,4_-H); 6.89 (s, 1H, pyridine-C_5_-H); 6.91 (d, *J* = 4.8 Hz, 1H, phenyl-C_4_-H); 7.26–7.33 (m, 3H, phenyl-C_2,5,6_-H); 7.65 (d, *J* = 8.4 Hz, 1H, quinoline- C_8_-H); 7.72 (d, *J* = 8.8 Hz, 1H, quinoline-C_7_-H); 7.95 (s, 1H, quinoline-C_5_-H); 8.19 (s, 1H, quinoline-C_4_-H); EI-MS m/z (% relative abundance): 458 [M^+•^+2] (24.70); 457 [M^+•^+1] (35.23); 456 [M^+•^] (100); 430 (70.40); 388 (54.53). Elemental analysis Calcd for C_26_H_21_ClN_4_O_2_ (456.93): C 68.34; H 4.63; N 12.26. Found: C 68.60, H 4.80, N 12.49.

#### 6-(3-Hydroxyphenyl)-4-(6-methoxy-2-(piperidin-1-yl)quinolin-3-yl)-2-oxo-1,2-dihydropyridine-3-carbonitrile (5f)

Yield, 40%; m.p 265–267 °C; IR (KBr, v cm^−1^): 3500 (OH), 3400 (NH), 2220 (C≡N), 1659 (C=O), 1598 (C=N). ^1^H-NMR (400 MHz, DMSO-d_6_, δ ppm): 1.51 (s, 6H, piperidine-C_3,4,5_-H); 3.15 (s, 4H, piperidine-C_2,4_-H); 3.86 (s, 3H, OCH_3_); 6.92 (brs, 1H, pyridine-C_5_-H); 6.98 (d, *J* = 7.2 Hz, 1H, phenyl-C_4_-H); 7.24 (s, 1H, phenyl-C_2_-H); 7.30–7.34 (m, 3H, quinoline-C_5,7_-H and phenyl-C_5_-H); 7.37 (d, *J* = 4 Hz, 1H, phenyl-C_6_-H); 7.70 (d, *J* = 9.2 Hz, 1H, quinoline-C_8_-H); 8.23 (s, 1H, quinoline-C_4_-H); 9.85 (s, 1H, OH, D_2_O-exchangeable); 12.73 (brs, 1H, NH, D_2_O-exchangeable). Elemental analysis Calcd for C_27_H_24_N_4_O_3_ (452.51): C 71.67; H 5.35; N 12.38. Found: C 71.90, H 5.26, N 12.56.

#### 4-(6-Substituted-2-morpholino or (piperidin-1-yl) quinolin-3-yl)-6-(3-hydroxyphenyl)-2-mercaptonicotinonitrile; (6a–e)

A mixture of 3-hydroxyacetophenone (1 mmol), substituted aldehyde **4a–f** (1 mmol), cyanothioacetamide (1 mmol) and ammonium acetate (8 mmol) was refluxed in ethanol (10 ml) with stirring for 3 h during which the product was obtained. The reaction mixture was cooled, and the yellow precipitate was filtered, washed with cold ethanol, air dried and recrystallized from ethanol/DMF (1:9) mixture.

#### 6-(3-Hydroxyphenyl)-4-(2-morpholin-4-yl-quinolin-3-yl)-2-mercaptopyridine-3-carbonitrile (6a)

Yield, 40%; m.p 240–242 °C; IR (KBr, v cm^−1^): 3389 (OH), 2590 (SH), 2221 (C≡N), 1599 (C=N). ^1^H-NMR (400 MHz, DMSO-d_6_, δ ppm): 3.05–3.20 (m, 4H, morpholine-C_2,6_-H); 3.42–3.50 (m, 4H, morpholine-C_3,5_-H); 6.89 (dd, *J* = 2,8 Hz, 1H, phenyl-C_4_-H); 7.19 (t, *J* = 8,7.6 Hz, 1H, phenyl-C_5_-H); 7.47 (s, 1H, phenyl-C_2_-H); 7.51 (t, *J* = 7.6 Hz 1H, quinoline-C_6_-H); 7.59 (d, *J* = 8 Hz, 1H, quinoline-C_5_-H); 7.77 (t, *J* = 7.6,8 Hz, 1H, quinoline-C_7_-H); 7.86 (d, *J* = 8.4 Hz, 1H, phenyl-C_6_-H); 7.96 (d, *J* = 7.6 Hz, 1H, quinoline-C_8_-H); 8.28 (s, 1H, pyridine-C_5_-H); 8.47 (s, 1H, quinoline-C_4_-H); 9.68 (s, 1H, OH, D_2_O-exchangeable); 14.15 (s, 1H, SH, D_2_O-exchangeable). Elemental analysis Calcd for C_25_H_20_N_4_O_2_S (440.52): C 68.16; H, 4.58; N, 12.72. Found: C 68.42, H 4.75, N 12.94.

#### 4-(6-Chloro-2-morpholin-4-yl-quinolin-3-yl)-6-(3-hydroxyphenyl)-2-mercaptopyridine-3-carbonitrile (6b)

Yield, 30%; m.p 260–261 °C; IR (KBr, v cm^−1^): 3451 (OH), 2595 (SH), 2202 (C≡N), 1633 (C=N). ^1^H-NMR (400 MHz, DMSO-d_6_, δ ppm): 3.60–3.65 (m, 4H, morpholine-C_2,6_-H, under DMSO); 3.75–3.78 (m, 4H, morpholine-C_3,5_-H, under DMSO); 6.96 (d, *J* = 8 Hz, 1H, phenyl-C_4_-H); 7.35 (t, *J* = 4,8 Hz, 1H, phenyl-C_5_-H); 7.60–7.81 (m, 5H, quinoline-C_5,7,8_-H and phenyl-C_2,6_-H); 8.42 (s, 1H, pyridine-C_5_-H); 9.49 (s, 1H, quinoline-C_4_-H); 9.75 (s, 1H, OH, D_2_O-exchangeable); 14.56 (s, 1H, SH, D_2_O-exchangeable). MS m/z (% relative abundance): 476 [M^+•^+2] (6.71); 474 [M^+•^] (23.44); 425 (72.98); 334 (100); 190 (84.18); 161 (82.30). Elemental analysis Calcd for C_25_H_19_ClN_4_O_2_S (474.96): C 63.22, H 4.03, N 11.80. Found: C 63.45, H 4.11, N 11.94

#### 6-(3-Hydroxyphenyl)-4-(6-methoxy-2-morpholin-4-yl-quinolin-3-yl)-2-mercaptopyridine-3-carbonitrile (6c)

Yield, 40%; m.*p* > 300 °C; IR (KBr, v cm^−1^): 3392 (OH), 2600 (SH), 2230 (C≡N), 1602 (C=N). ^1^H-NMR (400 MHz, DMSO-d_6_, δ ppm): 2.90–3.10 (m, 4H, morpholine-C_2,6_-H); 3.99–3.45 (m, 4H, morpholine-C_3,5_-H); 3.90 (s, 3H, OCH_3_); 6.89 (d, *J* = 8 Hz, 1H, phenyl-C_4_-H); 7.19 (t, *J* = 8 Hz, 1H, phenyl-C_5_-H); 7.37 (s, 1H, phenyl-C_2_-H); 7.42–7.47 (m, 2H, quinoline-C_5,7_-H); 7.59 (d, *J* = 8 Hz, 1H, quinoline-C_8_-H); 7.80 (d, *J* = 8 Hz, 1H, phenyl-C_6_-H); 8.27 (s, 1H, pyridine-C_5_-H); 8.37 (s, 1H, quinoline-C_4_-H); 9.67 (s, 1H, OH, D_2_O-exchangeable). ^13^C-NMR (100 MHz, DMSO-d_6_δ ppm): 49.99 (morpoline-C_2,5_); 56.01 (OCH_3_); 66.19 (morpholine-C_3.5_); 104.62 (pyridine-C_5_); 107.08 (quinoline-C_5_); 114.52 (pyridine-C_3_); 115.57 (CN); 118.53 (phenyl-C_2_); 118.59 (phenyl-C_4_); 119.00 (phenyl-C_6_); 123.40 (quinoline-C_3_); 123.66 (quinoline-C_7_); 125.73 (quinoline-C_4a_); 129.09 (quinoline-C_8_); 130.44 (phenyl-C_5_); 137.93 (quinoline-C_4_); 139.95 (phenyl-C_1_); 143.07 (quinoline-C_8a_); 153.49 (quinoline-C_6_); 156.49 (phenyl-C_3_); 156.76 (C=S); 158.41 (pyridine-C_4_); 158.73 (quinoline-C_2_); 159.12 (pyridine-C_6_). Elemental analysis Calcd for C_26_H_22_N_4_O_3_S (470.55): C 66.37, H 4.71, N 11.91. Found: C 66.59, H 4.83, N 11.75.

#### 6-(3-Hydroxyphenyl)-4-(2-(piperidin-1-yl)quinolin-3-yl)-2-mercaptopyridine-3-carbonitrile (6d)

Yield, 40%; m.*p* > 300 °C; IR (KBr, v cm^−1^): 3400 (OH), 2600 (SH), 2220 (C≡N), 1610 (C=N). ^1^H-NMR (400 MHz, DMSO-d_6_, δ ppm): 1.10–1.50 (m, 6H, piperidine-C_3,4,5_-H); 3.07 (s, 4H, piperidine-C_2,6_-H); 6.88 (dd, *J* = 2, 8 Hz, 1H, phenyl-C_4_-H); 7.17 (t, *J* = 8 Hz, 1H, phenyl-C_5_-H); 7.46–7.50 (m, 2H, quinoline-C_6_-H and phenyl-C_2_-H); 7.60 (d, *J* = 7.6 Hz, 1H, quinoline-C_5_-H); 7.74 (t, *J* = 7.6 Hz, 1H, quinoline-C_7_-H); 7.82 (d, *J* = 8.4 Hz, 1H, phenyl-C_6_-H); 7.92 (d, 1H, quinoline-C_8_-H); 8.24 (s, 1H, pyridine-C_5_-H); 8.40 (s, 1H, quinoline-C_4_-H); 9.65 (s, 1H, OH, D_2_O-exchangeable). Elemental analysis Calcd for C_26_H_22_N_4_OS (438.55): C 71.21, H 5.06, N 12.78. Found: C 71.47, H 5.82, N 13.02.

#### 6-(3-Hydroxyphenyl)-4-(6-methoxy-2-(piperidin-1-yl)quinolin-3-yl)-2-mercaptopyridine-3-carbonitrile (6e)

Yield, 40%; m.*p* > 300 °C; IR (KBr, v cm^−1^): 3403 (OH), 2214 (C≡N), 1602 (C=N). ^1^H-NMR (400 MHz, DMSO-d_6_, δ ppm): 1.15–1.45 (m, 6H, piperidine-C_3,4,5_-H); 2.90–3.15 (m, 4H, piperidine-C_2,6_-H); 3.89 (s, 3H, OCH_3_); 6.88 (d, *J* = 8 Hz, 1H, phenyl-C_4_-H); 7.17 (t, *J* = 8,8 Hz, 1H, phenyl-C_5_-H); 7.34 (s, 1H, quinoline-C_5_-H); 7.40 (d, *J* = 8 Hz, 1H, quinoline-C_7_-H); 7.51 (s, 1H, phenyl-C_2_-H); 7.61 (d, *J* = 8 Hz, 1H, quinoline-C_8_-H); 7.76 (d, *J* = 8 Hz, 1H, phenyl-C_6_-H); 8.24 (s, 1H, pyridine-C_5_-H); 8.31 (s, 1H, quinoline-C_4_-H); 9.68 (s, 1H, OH, D_2_O-exchangeable). ^13^C-NMR (100 MHz, DMSO-d_6_δ ppm): 24.36 (piperdine-C_4_); 25.46 (piperdine-C_3,5_); 50.69 (piperdine-C_2,6_); 55.98 (OCH_3_); 104.68 (pyridine-C_5_); 107.07 (quinoline-C_5_); 114.55 (pyridine-C_3_); 115.54 (CN); 116.66 (phenyl-C_2_); 118.55 (phenyl-C_4_); 118.93 (phenyl-C_6_); 123.14 (quinoline-C_3_); 124.05 (quinoline-C_7_); 125.47 (quinoline-C_4a_); 128.98 (quinoline-C_8_); 130.40 (phenyl-C_5_); 137.93 (quinoline-C_4_); 139.60 (phenyl-C_1_); 143.20 (quinoline-C_8a_); 153.92 (quinoline-C_6_); 156.49 (phenyl-C_3_); 157.49 (C=S); 158.44 (pyridine-C_4_); 158.55 (quinoline-C_2_); 159.14 (pyridine-C_6_). EI-MS m/z (% relative abundance): 468 [M^+•^] (22.75); 291 (73.83); 275 (100), 124 (79.94). Elemental analysis Calcd for C_27_H_24_N_4_O_2_S (468.58): C 69.21, H 5.16, N 11.96. Found: C 69.43, H 5.34, N 12.13.

### General procedure for synthesis of 3-(5-Cyano-4-(2-morpholin-4-yl-6-subsitituedquinolin-3-yl)-6-oxo-1,6-dihydropyridin-2-yl)phenyl acetate; (7a–c)

To a suspension of pyridone derivatives **5a–c** (1 mmol) in acetic acid (10 ml), acetic anhydride (0.141 ml, 1.5 mmol) was added. The reaction mixture was refluxed for 3–5 h, then was quenched with ice cold water. The precipitate formed was collected by filtration, air dried and crystallised from ethanol to afford the desired compounds.

#### 3-(5-Cyano-4-(2-morpholin-4-yl-quinolin-3-yl)-6-oxo-1,6-dihydropyridin-2-yl)phenyl acetate (7a)

Yield, 93%; m.*p* > 300 °C; IR (KBr, v cm^−1^): 3400 (NH), 2220 (C≡N), 1762 (COCH_3_), 1643 (C=O), 1615 (C=N). ^1^H-NMR (400 MHz, DMSO-d_6_, δ ppm): 2.31 (s, 3H, CH_3_); 3.20–3.25 (m, 4H, morpholine-C_2,6_-H); 3.58–3.63 (m, 4H, morpholine-C_3,5_-H); 7.10 (brs, 1H, pyridine-C_5_-H); 7.35 (d, *J* = 8, 1H, phenyl-C_4_-H); 7.47 (t, *J* = 4,7.6 Hz, 1H, quinoline-C_6_-H); 7.60 (t, *J* = 4,7.6 Hz, 1H, quinoline-C_7_-H); 7.74 (t, *J* = 4,7.6 Hz,1H, phenyl-C_5_-H); 7.79–7.81 (m, 3H, phenyl-C_2,6_-H and quinoline-C_8_-H); 7.91 (d, *J* = 7.6 Hz, 1H, quinoline-C_5_-H); 8.39 (s, 1H, quinoline-C_4_-H); 12.87 (s, 1H, NH, D_2_O-exchangeable). Elemental analysis Calcd for C_27_H_22_N_4_O_4_ (466.50): C 69.52, H 4.75, N 12.01. Found: C 69.30, H 4.94, N 12.29.

#### 3-(4-(6-Chloro-2-morpholin-4-yl-quinolin-3-yl)-5-cyano-6-oxo-1,6-dihydropyridin-2-yl)phenyl acetate (7b)

Yield, 92%; m.p 189–190 °C; IR (KBr, v cm^−1^): 3448 (NH), 2221(C≡N), 1763 (COCH_3_), 1644 (C=O), 1600 (C=N). ^1^H-NMR (400 MHz, DMSO-d_6_, δ ppm): 2.30 (s, 3H, CH_3_); 3.20–3.31 (m, 4H, morpholine-C_2,6_-H); 3.55–3.70 (m, 4H, morpholine-C_3,5_-H); 7.14 (brs, 1H, pyridine-C_5_-H); 7.36 (d, *J* = 7.6 Hz, 1H, phenyl-C_4_-H); 7.61 (t, *J* = 8 Hz, 1H, phenyl-C_5_-H); 7.72 (dd, *J* = 2,8.8 Hz, 1H, quinoline-C_7_-H); 7.75–7.82 (m, 2H, phenyl-C_2,6_-H); 7.86 (d, *J* = 8 Hz, 1H, quinoline-C_8_-H); 8.02 (d, *J* = 2 Hz, 1H, quinoline-C_5_-H); 8.37 (s, 1H, quinoline-C_4_-H); 12.88 (s, 1H, NH, D_2_O-exchangeable). Elemental analysis Calcd for C_27_H_21_ClN_4_O_4_ (500.94): C 64.74, H 4.23, N 11.18. Found: C 64.58, H 4.59, N 11.34.

#### 3-(5-Cyano-4-(6-methoxy-2-morpholin-4-yl-quinolin-3-yl)-6-oxo-1,6-dihydropyridin-2-yl)phenyl acetate (7c)

Yield, 91%; m.*p* > 300 °C; IR (KBr, v cm^−1^): 3450 (NH), 2218 (C≡N), 1760 (COCH_3_), 1646 (C=O), 1599 (C=N). ^1^H-NMR (400 MHz, DMSO-d_6_, δ ppm): 2.30 (s, 3H, CH_3_); 3.10–3.20 (m, 4H, morpholine-C_2,6_-H); 3.57–3.64 (m, 4H, morpholine-C_3,5_-H); 3.87 (s, 3H, OCH_3_); 7.04 (brs, 1H, pyridine-C_5_-H); 7.35–7.36 (m, 2H, quinoline-C_5_-H and phenyl-C_4_-H); 7.39 (dd, *J* = 2.8,9 Hz, 1H, quinoline-C_7_-H); 7.60 (t, *J* = 8 Hz, 1H, phenyl-C_5_-H); 7.73–7.83 (m, 3H, phenyl-C_2,6_-H and quinoline-C_8_-H); 8.30 (s, 1H, quinoline-C_4_-H); 12.88 (s, 1H, NH, D_2_O-exchangeable). ^13^C-NMR (100 MHz, DMSO-d_6_δ ppm): 21.29, 49.93, 55.96, 66.38, 100.00, 106.98, 115.45, 115.93, 121.22, 122.43, 123.32, 125.21, 127.50, 128.35, 128.89, 130.67, 135.83, 139.57, 142.98, 145.00, 151.32, 152.51, 156.52, 163.97, 169.65, 174.50. Elemental analysis Calcd for C_28_H_24_N_4_O_5_ (496.52): C 67.73, H 4.87, N 11.28. Found: C 67.54, H 5.01, N 11.49.

### General procedure for synthesis of ethyl 2-((6-(3-acetoxyphenyl)-4-(2-morpholin-4-yl-6-substituted quinolin-3-yl)-3-cyanopyridin-2-yl)oxy)acetate (8a–c)

A mixture of phenylacetate derivatives **7a–c** (1 mmol), ethylbromoacetate (0.110 ml, 1 mmol) and anhydrous potassium carbonate (0.207 g, 1.5 mmol) in dry acetone (10 ml) was heated under reflux for 4 h then the reaction mixture was cooled and triturated with water till complete precipitation of the product. The green precipitate was filtered, washed with water, air dried and crystallised from ethanol.

#### Ethyl 2-((6-(3-acetoxyphenyl)-3-cyano-4-(2-morpholin-4-yl-quinolin-3-yl)pyridin-2-yl)oxy)acetate (8a)

Yield, 92%; m.p 129–130 °C; IR (KBr, v cm^−1^): 2224 (C≡N), 1760, 1750 (C=O), 1592 (C=N). ^1^H-NMR (400 MHz, DMSO-d_6_, δ ppm): 1.21 (t, *J* = 7 Hz, 3H, CH_3_); 2.32 (s, 3H, COCH_3_); 3.10–3.19 (m, 4H, morpholine-C_2,6_-H); 3.55–3.62 (m, 4H, morpholine-C_3,5_-H); 4.20 (q, *J* = 7 Hz, 2H, OCH_2_); 5.22 (s, 2H, CH_2_); 7.34 (dd, *J* = 1.2,8 Hz, 1H, phenyl-C_4_-H); 7.49 (t, *J* = 7.1,7.8 Hz, 1H, quinoline-C_6_-H); 7.61 (t, *J* = 8 Hz, 1H, phenyl-C_5_-H); 7.76 (t, *J* = 7.1,8.4 Hz, 1H, quinoline-C_7_-H); 7.85 (d, *J* = 8.4 Hz, 1H, quinoline-C_8_-H); 7.93–7.95 (m, 2H, quinoline-C_5_-H and pyridine-C_5_-H); 8.13 (d, *J* = 8 Hz, 1H, phenyl-C_6_-H); 8.20 (s, 1H, quinoline-C_4_-H); 8.45 (s, 1H, phenyl-C_2_-H) .

#### Ethyl 2-((6-(3-acetoxyphenyl)-4-(6-chloro-2-morpholin-4-yl-quinolin-3-yl)-3-cyanopyridin-2-yl)oxy)acetate (8b)

Yield, 89%; m.p 228–230 °C; IR (KBr, v cm^−1^): 2224 (C≡N), 1756 (C=O); 1593 (C=N).

#### Ethyl 2-((6-(3-acetoxyphenyl)-3-cyano-4-(6-methoxy-2-morpholin-4-yl-quinolin-3-yl)pyridin-2-yl)oxy)acetate (8c)

Yield, 90%; m.p 204–206 °C; IR (KBr, v cm^−1^): 2224 (C≡N), 1780, 1758 (C=O), 1595 (C=N).

### General procedure for synthesis of 2-((3-Cyano-6-(3-hydroxyphenyl)-4-(2-morpholin-4-yl-6-substituted quinolin-3-yl)pyridin-2-yl)oxy)acetic acid (9a–c)

A mixture of compounds **8a–c** (1 mmol), and sodium bicarbonate (0.756 g, 9 mmol) in methanol\water (1:1) was refluxed for 1 h. The reaction mixture was then cooled, triturated with water and neutralised with acetic acid (PH = 7). The precipitate of the product was collected, filtered, washed with water, air dried and crystallised from ethanol.

#### 2-((3-Cyano-6-(3-hydroxyphenyl)-4-(2-morpholin-4-yl-quinolin-3-yl)pyridin-2-yl)oxy)acetic acid (9a)

Yield, 90%; m.p 179–180 °C; IR (KBr, v cm^−1^): 3403 (OH), 3350–3050 (COOH), 2224 (C≡N), 1732 (C=O), 1643 (C=N). ^1^H-NMR (400 MHz, DMSO-d_6_, δ ppm): 3.09–3.19 (m, 4H, morpholine-C_2,6_-H); 3.55–3.62 (m, 4H, morpholine-C_3,5_-H); 5.15 (s, 2H, CH_2_); 6.95 (d, *J* = 7.6 Hz, 1H, phenyl-C_4_-H); 7.34 (t, *J* = 7.6,8 Hz, 1H, phenyl-C_5_-H); 7.48 (t, *J* = 7.2,7.6 Hz, 1H, quinoline-C_6_-H); 7.60 (s, 1H, phenyl-C_2_-H) ; 7.65 (d, *J* = 7.6 Hz, 1H, quinoline-C_8_-H); 7.75 (t, *J* = 7.2, 7.6 Hz, 1H, quinoline-C_7_-H); 7.84 (d, *J* = 8 Hz, 1H, phenyl-C_6_-H); 7.93 (d, *J* = 7.6 Hz, 1H, quinoline-C_5_-H); 8.02 (s, 1H, pyridine-C_5_-H); 8.43 (s, 1H, quinoline-C_4_-H); 9.77, 13.22 (2brs, each for1H, 2OH, D_2_O-exchangeable). ^13^C-NMR (100 MHz, DMSO-d_6_δ ppm): 49.69, 63.77, 66.15, 93.33, 114.52, 114.78, 115.30, 118.44, 118.67, 123.70, 124.71, 125.21, 127.42, 128.71, 130.47, 131.43, 138.06, 140.78, 147.29, 155.38, 157.79, 158.08, 158.37, 163.38, 170.09. Elemental analysis Calcd for C_27_H_22_N_4_O_5_ (482.50): C 67.21, H 4.60, N 11.61. Found: C 67.49, H 4.83, N 11.88.

#### 2-((4-(6-Chloro-2-morpholin-4-yl-quinolin-3-yl)-3-cyano-6-(3-hydroxyphenyl)pyridin-2-yl)oxy)acetic acid (9b)

Yield, 89%; m.p 279–280 °C; IR (KBr, v cm^−1^): 3448 (OH), 3600–3000 (COOH), 2225 (C≡N), 1626 (C=O), 1597 (C=N). ^1^H-NMR (400 MHz, DMSO-d_6_, δ ppm): 3.02–3.30 (m, 4H, morpholine-C_2,6_-H); 3.50–3.70 (4H, under DMSO, morpholine-C_3,5_-H); 4.96 (s, 2H, CH_2_); 6.89 (d, *J* = 7.6 Hz, 1H, phenyl-C_4_-H); 7.26 (t, *J* = 7.6,8 Hz, 1H, phenyl-C_5_-H); 7.58–7.60 (m, 2H, quinoline-C_5_-H and phenyl-C_6_-H); 7.73 (d, *J* = 9 Hz, 1H, quinoline-C_7_-H) ; 7.82 (d, *J* = 9 Hz, 1H, quinoline-C_8_-H); 7.88 (s, 1H, phenyl-C_2_-H); 8.03 (s, 1H, pyridine-C_5_-H); 8.32 (s, 1H, quinoline-C_4_-H); 10.09 (brs, 1H, OH, D_2_O-exchangeable). EI-MS m/z (% relative abundance): 518 [M^+•^+2] (21.67); 516 [M^+•^] (32.78); 413 (100); 391 (86.16). Elemental analysis Calcd for C_27_H_21_ClN_4_O_5_ (516.94): C 62.73, H 4.09, N 10.84. Found: C 62.96, H 4.13, N 11.06.

#### 2-((3-Cyano-6-(3-hydroxyphenyl)-4-(6-methoxy-2-morpholin-4-yl-quinolin-3-yl)pyridin-2-yl)oxy)acetic acid (9c)

Yield, 92%; m.p 274–275 °C; IR (KBr, v cm^−1^): 3422 (OH), 3600–3050 (COOH), 2224 (C≡N), 1598 (C=O), 1549 (C=N). ^1^H-NMR (400 MHz, DMSO-d_6_, δ ppm): 3.02–3.12 (m, 4H, morpholine-C_2,6_-H); 3.55–3.60 (m, 4H, morpholine-C_3,5_-H); 3.87 (s, 3H, OCH_3_); 4.89 (s, 2H, -CH_2_); 6.92 (d, *J* = 8.4 Hz, 1H, phenyl-C_4_-H); 7.31 (t, *J* = 8,8.4 Hz, 1H, phenyl-C_5_-H); 7.38–7.41 (m, 2H, quinoline-C_5,7_-H); 7.60–7.67 (m, 2H, phenyl-C_2_-H and quinoline-C_8_-H); 7.77 (d, *J* = 8 Hz, 1H, phenyl-C_6_-H); 7.90 (s, 1H, pyridine-C_5_-H); 8.30 (s, 1H, quinoline-C_4_-H); 9.84, 13.10 (2brs, each for 1H, 2OH, D_2_O-exchangeable). Elemental analysis Calcd for C_28_H_24_N_4_O_6_ (512.52): C 65.62, H 4.72, N 10.93. Found: C 65.90, H 4.89, N 11.24.

### General procedure for synthesis of 3-(5-cyano-4-(2-morpholin-4-yl-6-substitiutedquinolin-3-yl)-6-(2-((4-substitutedphenyl)amino)-2-oxoethoxy)pyridin-2-yl)phenyl acetate; (10a–i)

To a mixture of compounds **7a–c** (1 mmol), anhydrous potassium carbonate (0.207 g 1.5 mmol) in dry acetone (10 ml) the appropriate chloroacetanilide derivatives (1 mmol) were added and the reaction mixture was heated under reflux for 4 h. The reaction mixture was then cooled and treated with ice-cold water. The obtained product was collected by filtration and crystallised from ethanol.

#### 3-(5-Cyano-4-(2-morpholin-4-yl-quinolin-3-yl)-6-(2-oxo-2-(phenylamino)ethoxy)pyridin-2-yl)phenyl acetate (10a)

Yield, 90%; m.p 178–180 °C; IR (KBr, v cm^−1^): 3447 (NH), 2220 (C≡N), 1761 (C=O), 1677 (CONH), 1651 (C=N).

#### 3-(6-(2-((4-Chlorophenyl)amino)-2-oxoethoxy)-5-cyano-4-(2-morpholin-4-yl-quinolin-3-yl)pyridin-2-yl)phenyl acetate (10b)

Yield, 89%; m.p 209–210 °C; IR (KBr, v cm^−1^): 3407 (NH), 2223 (C≡N), 1763 (C=O), 1679 (CONH), 1590 (C=N). ^1^H-NMR (400 MHz, DMSO-d_6_, δ ppm): 2.21 (s, 3H, COCH_3_); 3.10–3.20 (m, 4H, morpholine-C_2,6_-H); 3.50–3.65 (m, 4H, morpholine-C_3,5_-H); 5.21 (s, 2H, CH_2_); 7.26 (d, *J* = 8 Hz, 1H, phenyl-C_4_-H); 7.39 (d, *J* = 8.4 Hz, 2H, p-chlorophenyl-C_3,5_-H); 7.47–7.51 (m, 2H, quinoline-C_6_-H and phenyl-C_5_-H); 7.65 (d, *J* = 8.4 Hz, 2H, p-chlorophenyl-C_2,6_-H); 7.76 (t, *J* = 6.8,8 Hz, 1H, quinoline-C_7_-H); 7.85 (d, *J* = 8 Hz, 1H, quinoline-C_8_-H); 7.93–7.95 (m, 2H, quinoline-C_5_-H and pyridine-C_5_-H); 8.12 (d, *J* = 7.6 Hz, 1H, phenyl-C_6_-H); 8.16 (s, 1H, quinoline-C_4_-H); 8.43 (s, 1H, phenyl-C_2_-H); 10.61 (s, 1H, NH, D_2_O-exchangeable).

#### 3-(5-Cyano-6-(2-((4-methoxyphenyl)amino)-2-oxoethoxy)-4-(2-morpholin-4-yl-quinolin-3-yl)pyridin-2-yl)phenyl acetate (10c)

Yield, 92%; m.p 268–270 °C; IR (KBr, v cm^−1^): 3414 (NH), 2225 (C≡N), 1764 (C=O), 1671 (CONH), 1591 (C=N).

#### 3-(4-(6-Chloro-2-morpholin-4-yl-quinolin-3-yl)-5-cyano-6-(2-oxo-2-(phenylamino)ethoxy)pyridin-2-yl)phenyl acetate (10d)

Yield, 91%; m.p 197–198 °C; IR (KBr, v cm^−1^): 3416 (NH), 2224 (C≡N), 1744 (COCH_3_), 1680 (CONH), 1543 (C=N).

#### 3-(4-(6-Chloro-2-morpholin-4-yl-quinolin-3-yl)-6-(2-((4-chlorophenyl)amino)-2-oxoethoxy)-5-cyanopyridin-2-yl)phenyl acetate (10e)

Yield, 89%; m.p 188–190 °C; IR (KBr, v cm^−1^): 3420 (NH), 2220 (C≡N), 1743 (OCOCH_3_), 1680 (CONH).

#### 3-(4-(6-Chloro-2-morpholin-4-yl-quinolin-3-yl)-5-cyano-6-(2-((4-methoxyphenyl)amino)-2-oxoethoxy)pyridin-2-yl)phenyl acetate (10f)

Yield, 93%; m.p 188–190 °C; IR (KBr, v cm^−1^): 3414 (NH), 2223 (C≡N), 1761 (C=O), 1667 (CONH), 1592 (C=N). ^1^H-NMR (400 MHz, DMSO-d_6_, δ ppm): 2.23 (s, 3H, COCH_3_); 3.15–3.18 (m, 4H, morpholine-C_2,6_-H); 3.58–3.60 (m, 4H, morpholine-C_3,5_-H); 3.73 (s, 3H, OCH_3_); 5.20 (s, 2H, CH_2_); 6.91 (d, *J* = 8 Hz, 2H, p-methoxyphenyl-C_3,5_-H); 7.28 (d, *J* = 8 Hz, 1H, phenyl-C_4_-H); 7.50–7.54 (m, 3H, p-methoxyphenyl-C_2,6_-H and phenyl-C_5_-H); 7.76 (d, *J* = 8 Hz, 1H, quinoline-C_7_-H); 7.86 (d, *J* = 8 Hz, 1H, quinoline-C_8_-H); 7.97 (s, 1H, quinoline-C_5_-H); 8.05 (s, 1H, pyridine-C_5_-H); 8.14 (d, *J* = 8 Hz, 1H, phenyl-C_6_-H); 8.17 (s, 1H, quinoline-C_4_-H); 8.41 (s, 1H, phenyl-C_2_-H); 10.33 (s, 1H, NH, D_2_O-exchangeable).

#### 3-(5-Cyano-4-(6-methoxy-2-morpholin-4-yl-quinolin-3-yl)-6-(2-oxo-2-(phenylamino)ethoxy)pyridin-2-yl)phenyl acetate (10g)

Yield, 90%; m.p 284–285 °C; IR (KBr, v cm^−1^): 3420 (NH), 2223 (C≡N), 1759 (C=O), 1678 (CONH), 1597 (C=N). ^1^H-NMR (400 MHz, DMSO-d_6_, δ ppm): 2.20 (s, 3H, COCH_3_); 3.07–3.09 (m, 4H, morpholine-C_2,6_-H); 3.57–3.60 (m, 4H, morpholine-C_3,5_-H); 3.89 (s, 3H, OCH_3_); 5.23 (s, 2H, CH_2_); 7.09 (s, 1H, quinoline-C_5_-H); 7.25–7.49 (m, 5H, phenyl-C_3,4,5_-H, quinoline-C_7_-H and phenylacetate-C_4_-H); 7.61–7.64 (m, 2H, phenyl-C_2,6_-H); 7.77–7.81 (m, 2H, phenylacetate-C_5_-H and quinoline-C_8_-H); 7.98 (s, 1H, pyridine-C_5_-H); 8.12–8.16 (m, 2H, quinoline-C_5_-H and phenylacetate-C_6_-H); 8.36 (s, 1H, phenylacetae-C_2_-H); 10.48 (s, 1H, NH, D_2_O-exchangeable).

#### 3-(6-(2-((4-Chlorophenyl)amino)-2-oxoethoxy)-5-cyano-4-(6-methoxy-2-morpholin-4-yl-quinolin-3-yl)pyridin-2-yl)phenyl acetate (10h)

Yield, 92%; m.p 264–265 °C; IR (KBr, v cm^−1^): 3421 (NH), 2225 (C≡N), 1748 (C=O), 1680 (CONH), 1651 (C=N).

#### 3-(5-Cyano-4-(6-methoxy-2-morpholin-4-yl-quinolin-3-yl)-6-(2-((4-methoxyphenyl)amino)-2-oxoethoxy)pyridin-2-yl)phenyl acetate (10i)

Yield, 93%; m.p 270–271 °C; IR (KBr, v cm^−1^): 3419 (NH), 2222 (C≡N), 1762 (C=O), 1670 (CONH), 1599 (C=N).

### General procedure for synthesis of 2-((3-Cyano-6-(3-hydroxyphenyl)4-(2-morpholin-4-yl-6-substituted quinolin-3-yl)pyridin-2-yl)oxy)-N-(4-substituted phenyl)acetamide (11a–i)

A mixture of compounds **10a–i** (1 mmol), and sodium bicarbonate (0.756 g, 9 mmol) in equal amount of methanol\water (1:1) was refluxed for 1 h. The reaction mixture was then cooled, treated with water and neutralised with acetic acid (PH = 7). The obtained product was filtered, washed with water, air dried and crystallised from ethanol.

#### 2-((3-Cyano-6-(3-hydroxyphenyl)-4-(2-morpholin-4-yl-quinolin-3-yl)pyridin-2-yl)oxy)-N-phenylacetamide (11a)

Yield, 92%; m.p 243–245 °C; IR (KBr, v cm^−1^): 3550 (OH), 3392 (NH), 2220 (C≡N), 1670 (CONH), 1585 (C=N). ^1^H-NMR (400 MHz, DMSO-d_6_, δ ppm): 3.10–3.20 (m, 4H, morpholine-C_2,6_-H); 3.50–3.65 (m, 4H, morpholine-C_3,5_-H); 5.23 (s, 2H, CH_2_); 6.87 (d, *J* = 7.6 Hz, 1H, 3-hydroxyphenyl-C_4_-H); 7.06 (t, *J* = 7.2,7.6 Hz, 1H, 3-hydroxyphenyl-C_5_-H); 7.17 (t, *J* = 7.6,8 Hz, 1H, quinoline-C_6_-H); 7.31 (t, *J* = 7.2,7.6 Hz, 2H, phenyl-C_3,5_-H); 7.48 (t, *J* = 7.2 Hz, 1H, phenyl-C_4_-H); 7.58–7.63 (m, 4H, 3-hydroxyphenyl-C_2,6_-H and phenyl-C_2,6_-H); 7.74 (t, *J* = 7.6,7.2 Hz, 1H, quinoline-C_7_-H); 7.83 (d, *J* = 8 Hz, 1H, quinoline-C_8_-H); 7.93 (d, *J* = 8 Hz, 1H, quinoline-C_5_-H); 7.97 (s, 1H, pyridine-C_5_-H); 8.40 (s, 1H, quinoline-C_4_-H); 10.49 (s, 1H, NH, D_2_O-exchangeable). ^13^C-NMR (100 MHz, DMSO-d_6_δ ppm): 49.68, 65.82, 66.17, 93.23, 114.53, 114.77, 115.43, 118.19, 118.58, 120.01, 123.71, 123.97, 124.69, 125.18, 127.40, 128.71, 128.71, 130.26, 131.42, 137.92, 139.05, 140.77, 147.28, 155.31, 157.79, 158.18, 159.05, 163.46, 166.47. Elemental analysis Calcd for C_33_H_27_N_5_O_4_ (557.61): C 71.08, H 4.88, N 12.56. Found: C 70.89, H 4.96, N 12.31.

#### N-(4-Chlorophenyl)-2-((3-cyano-6-(3-hydroxyphenyl)-4-(2-morpholin-4-yl-quinolin-3-yl)pyridin-2-yl)oxy)acetamide (11b)

Yield, 89%; m.p 279–280 °C; IR (KBr, v cm^−1^): 3500 (OH), 3396 (NH), 2224 (C≡N), 1659 (CONH), 1584 (C=N). ^1^H-NMR (400 MHz, DMSO-d_6_, δ ppm): 3.10–3.25 (m, 4H, morpholine-C_2,6_-H); 3.50–3.70 (m, 4H, morpholine-C_3,5_-H); 5.23 (s, 2H, CH_2_); 6.80–6.95 (m, 1H, phenyl-C_4_-H); 7.15–7.25 (m, 1H, phenyl-C_5_); 7.31–8.15 (m, 10H, p-chlorophenyl-C_2,3,5,6_-H, quinoline-C_5,6,7,8_-H and phenyl-C_2,5_-H); 8.00 (s, 1H, pyridine-C_5_-H); 8.42 (s, 1H, quinoline-C_4_-H); 9.66 (brs, 1H, OH, D_2_O-exchangeable); 10.59 (s, 1H, NH, D_2_O-exchangeable). Elemental analysis Calcd for C_33_H_26_ClN_5_O_4_ (592.05): C 66.95, H 4.43, N 11.83. Found: C 67.13, H 4.60, N 12.06.

#### 2-((3-Cyano-(3-hydroxyphenyl)-4-(2-morpholin-4-yl-quinolin-3-yl)pyridin-2-yl)oxy)-N-(4-methoxyphenyl)acetamide (11c)

Yield, 90%; m.p 268–270 °C; IR (KBr, v cm^−1^): 3550 (OH), 3401 (NH), 2223 (C≡N), 1649 (CONH), 1583 (C=N). ^1^H-NMR (400 MHz, DMSO-d_6_, δ ppm): 3.05–3.20 (m, 4H, morpholine-C_2,6_-H); 3.50–3.60 (m, 4H, morpholine-C_3,5_-H); 3.72 (s, 3H, OCH_3_); 5.14 (s, 2H, CH_2_); 6.87 (d, *J* = 8 Hz, 2H, p-methoxyphenyl-C_3,5_-H); 6.90 (d, *J* = 8 Hz, 1H, phenyl-C_4_-H); 7.24 (t, *J* = 8 Hz, 1H, phenyl-C_5_-H); 7.44–7.49 (m, 3H, p-methoxyphenyl-C_2,6_-H and quinoline-C_6_); 7.55 (s, 1H, phenyl-C_2_-H); 7.64 (d, *J* = 8 Hz,1H, quinoline-C_8_-H); 7.74 (t, *J* = 8 Hz, 1H, quinoline-C_7_-H); 7.83 (d, *J* = 8 Hz, 1H, phenyl-C_6_-H); 7.92 (d, *J* = 8 Hz, 1H, quinoline-C_5_-H); 7.95 (s, 1H, pyridine-C_5_-H); 8.33 (s, 1H, quinoline-C_4_-H); 9.71 (s, 1H, OH, D_2_O-exchangeable); 10.26 (s, 1H, NH, D_2_O-exchangeable). ^13^C-NMR (100 MHz, DMSO-d_6_δ ppm): 49.69; 55.62, 65.82, 66.17, 93.39, 100.00, 114.37, 114.58, 118.77, 119.02, 121.64, 122.48, 123.71, 124.69, 127.41, 128.71, 130.37, 131.15, 131.45, 132.04, 139.05, 140.79, 147.29, 155.35, 155.90, 157.80, 158.03, 158.32, 163.52, 165.99. Elemental analysis Calcd for C_34_H_29_N_5_O_5_ (587.64): C 69.49, H 4.97, N 11.92. Found: C 69.32, H 5.13, N 12.18.

#### 2-((4-(6-Chloro-2-morpholin-4-yl-quinolin-3-yl)-3-cyano-6-(3-hydroxyphenyl)pyridin-2-yl)oxy)-N-phenylacetamide (11d)

Yield, 88%; m.p 277–280 °C; IR (KBr, v cm^−1^): 3550 (OH), 3437 (NH), 2225 (C≡N), 1657 (CONH), 1580 (C=N). ^1^H-NMR (400 MHz, DMSO-d_6_, δ ppm): 3.10–3.20 (m, 4H, morpholine-C_2,6_-H); 3.50–3.65 (m, 4H, morpholine-C_3,5_-H); 5.18 (s, 2H, -CH_2_); 6.89 (d, *J* = 8 Hz, 1H, 3-hydroxyphenyl-C_4_-H); 7.07 (t, *J* = 8 Hz, 1H, phenyl-C_4_-H); 7.20 (t, *J* = 8 Hz, 1H, 3-hydroxyphenyl-C_5_-H); 7.30 (t, *J* = 8 Hz, 2H, phenyl-C_3,5_-H); 7.56 (d, *J* = 8 Hz, 2H, phenyl-C_2,6_-H); 7.61 (d, *J* = 8 Hz, 1H, 3-hydroxyphenyl-C_6_-H); 7.72 (d, *J* = 8 Hz, 1H, quinoline-C_5_-H); 7.78–7.84 (m, 2H, 3-hydroxyphenyl-C_2_-H and quinoline-C_7_-H); 7.95 (s, 1H, pyridine-C_5_-H); 7.98 (d, *J* = 8 Hz, 1H, quinoline-C_8_-H); 8.31 (s, 1H, quinoline-C_4_-H); 9.88 (s, 1H, OH, D_2_O-exchangeable); 10.62 (s, 1H, NH, D_2_O-exchangeable). Elemental analysis Calcd for C_33_H_26_ClN_5_O_4_ (592.05): C 66.95, H 4.43, N 11.83. Found: C 66.81, H 4.62, N 11.94.

#### 2-((4-(6-Chloro-2-morpholin-4-yl-quinolin-3-yl)-3-cyano-6-(3-hydroxyphenyl)pyridin-2-yl)oxy)-N-(4-chlorophenyl)acetamide (11e)

Yield, 87%; m.*p* > 300 °C; IR (KBr, v cm^−1^): 3500 (OH), 3413 (NH), 2218 (C≡N), 1654 (CONH), 1595 (C=N). ^1^H-NMR (400 MHz, DMSO-d_6_, δ ppm): 3.10–3.20 (m, 4H, morpholine-C_2,6_-H); 3.50–3.65 (m, 4H, morpholine-C_3,5_-H); 5.23 (s, 2H, CH_2_); 6.91 (d, *J* = 8 Hz, 1H, phenyl-C_4_-H); 7.22 (t, *J* = 7.6,8 Hz, 1H, phenyl-C_5_-H); 7.38 (d, *J* = 8.8 Hz, 2H, p-chlorophenyl-C_3,5_-H); 7.58–7.66 (m, 1H, phenyl-C_6_-H); 7.65 (d, *J* = 8.8 Hz, 2H, p-chlorophenyl-C_2,6_-H); 7.75 (d, *J* = 8.8 Hz, 1H, quinoline-C_7_-H); 7.84 (d, *J* = 8.8 Hz, 1H, quinoline-C_8_-H); 8.00 (s, 1H, phenyl-C_2_-H); 8.04 (s, 1H, quinoline-C_5_-H); 8.32 (s, 1H, pyridine-C_5_-H); 8.39 (s, 1H, quinoline-C_4_-H); 9.75 (brs, 1H, OH, D_2_O-exchangeable); 10.59 (s, 1H, NH, D_2_O-exchangeable). EI-MS m/z (% relative abundance): 630 [M^+•^+4] (35.76); 626 [M^+•^] (40.95); 267 (92.07); 127 (100). Elemental analysis Calcd for C_33_H_25_Cl_2_N_5_O_4_ (626.49): C 63.27, H 4.02, N 11.18. Found: C 63.44, H 4.21, N 11.39.

#### 2-((4-(6-Chloro-2-morpholin-4-yl-quinolin-3-yl)-3-cyano-6-(3-hydroxyphenyl)pyridin-2-yl)oxy)-N-(4-methoxyphenyl)acetamide (11f)

Yield, 89%; m.p 228–230 °C; IR (KBr, v cm^−1^): 3450 (OH), 3403 (NH), 2224 (C≡N), 1658 (CONH), 1593 (C=N). ^1^H-NMR (400 MHz, DMSO-d_6_, δ ppm): 3.11–3.21 (m, 4H, morpholine-C_2,6_-H); 3.53–3.58 (m, 4H, morpholine-C_3,5_-H); 3.68 (s, 3H, OCH_3_); 5.14 (s, 2H, -CH_2_); 6.85–6.91 (m, 3H, phenyl-C_4_-H and p-methoxyphenyl-C_3,5_-H); 7.23 (t, *J* = 8 Hz, 1H, phenyl-C_5_-H); 7.46 (d, *J* = 8 Hz, 2H, p-methoxyphenyl-C_2,6_-H); 7.54 (s, 1H, quinoline-C_5_-H); 7.62 (d, *J* = 4 Hz, 1H, phenyl-C_2_-H); 7.71 (d, *J* = 8 Hz, 1H, quinoline-C_7_-H); 7.81 (d, *J* = 8 Hz, 1H, phenyl-C_6_-H); 7.93–7.98 (m, 2H, pyridine-C_5_-H and quinoline-C_8_-H); 8.28 (s, 1H, quinoline-C_4_-H); 9.73 (s, 1H, OH, D_2_O-exchangeable); 10.27 (s, 1H, NH, D_2_O-exchangeable). EI-MS m/z (% relative abundance): 624 [M^+•^+2] (29.04); 622 [M^+•^] (36.76); 366 (84.92); 182 (100). Elemental analysis Calcd for C_34_H_28_ClN_5_O_5_ (622.08): C 65.65, H 4.54, N 11.26. Found: C 65.47, H 4.63, N 11.45.

#### 2-((3-Cyano-6-(3-hydroxyphenyl)-4-(6-methoxy-2-morpholin-4-yl-quinolin-3-yl)pyridin-2-yl)oxy)-N-phenylacetamide (11g)

Yield, 91%; m.p 269–270 °C; IR (KBr, v cm^−1^): 3550 (OH), 3409 (NH), 2220 (C≡N), 1650 (CONH). ^1^H-NMR (400 MHz, DMSO-d_6_, δ ppm): 2.95–3.15 (m, 4H, morpholine-C_2,6_-H); 3.50–3.60 (m, 4H, morpholine-C_3,5_-H); 3.88 (s, 3H, OCH_3_); 5.23 (s, 2H, CH_2_); 6.91 (d, *J* = 8 Hz, 1H, 3-hydroxyphenyl-C_4_-H); 7.02–7.13 (m, 1H, quinoline-C_7_-H); 7.21 (s, 1H, quinoline-C_5_-H); 7.30–7.45 (m, 4H, phenyl-C_3,4,5_-H and 3-hydroxyphenyl-C_5_-H); 7.51–7.70 (m, 3H, phenyl-C_2,6_-H and 3-hydroxyphenyl-C_2_-H); 7.75–7.82 (m, 2H, 3-hydroxyphenyl-C_6_-H and quinoline-C_8_-H); 7.99 (s, 1H, pyridine-C_5_-H); 8.32 (s, 1H, quinoline-C_4_-H); 9.69 (brs, 1H, OH, D_2_O-exchangeable); 10.45 (s, 1H, NH, D_2_O-exchangeable). Elemental analysis Calcd for C_34_H_29_N_5_O_5_ (587.64): C 69.49, H 4.97, N 11.92. Found: C 69.31, H 5.14, N 12.13.

#### N-(4-Chlorophenyl)-2-((3-cyano-6-(3-hydroxyphenyl)-4-(6-methoxy-2-morpholin-4-yl-quinolin-3-yl)pyridin-2-yl)oxy)acetamide (11h)

Yield, 90%; m.p 230–232 °C; IR (KBr, v cm^−1^): 3550 (OH), 3428 (NH), 2220 (C≡N), 1650 (CONH). ^1^H-NMR (400 MHz, DMSO-d_6_, δ ppm): 3.09–3.20 (m, 4H, morpholine-C_2,6_-H); 3.53 (s, 4H, morpholine-C_3,5_-H); 3.87 (s, 3H, OCH_3_); 4.68, 5.54 (2 s, each for 1H, CH_2_); 6.90–7.00 (m, 1H, phenyl-C_4_-H); 7.25–7.50 (m, 4H, p-chlorophenyl-C_3_,_5_-H and quinoline-C_5,7_-H); 7.60–7.70 (m, 2H, phenyl-C_2,5_-H); 7.75–7.90 (m, 2H, p-chlrophenyl-C_2,6_-H); 7.90–7.95 (m, 1H, quinoline-C_8_-H); 8.00–8.10 (m, 1H, phenyl-C_6_-H); 8.20–8.35 (m, 2H, quinoline-C_4_-H and pyridine-C_5_-H); 9.87 (brs, 1H, OH, D_2_O-exchangeable); 10.37 (s, 1H, NH, D_2_O-exchangeable). Elemental analysis Calcd for C_34_H_28_ClN_5_O_5_ (622.08): C 65.65, H 4.54, N 11.26. Found: C 65.92, H 4.67, N 11.52.

#### 2-((3-Cyano-6-(3-hydroxyphenyl)-4-(6-methoxy-2-morpholin-4-yl-quinolin-3-yl)pyridin-2-yl)oxy)-N-(4-methoxyphenyl)acetamide (11i)

Yield, 93%; m.*p* > 300 °C; IR (KBr, v cm^−1^): 3500 (OH), 3435 (NH), 2220 (C≡N), 1654 (CONH). ^1^H-NMR (400 MHz, DMSO-d_6_, δ ppm): 3.00–3.15 (m, 4H, morpholine-C_2,6_-H); 3.56 (s, 4H, morpholine-C_3,5_-H); 3.72 (s, 3H, OCH_3_); 3.88 (s, 3H, OCH_3_); 5.21 (s, 2H, -CH_2_); 6.80–6.95 (m, 3H, phenyl-C_4_-H and p-methoxyphenyl-C_3,5_-H); 7.22 (s, 1H, quinoline-C_5_-H); 7.35–7.43 (m, 2H, p-methoxyphenyl-C_2,6_-H); 7.50–7.60 (m, 1H, phenyl-C_5_-H); 7.67–7.79 (m, 3H, phenyl-C_2,6_-H and quinoline-C_7_-H); 7.98–8.00 (m, 2H, pyridine-C_5_-H and quinoline-C_8_-H); 8.33 (s, 1H, quinoline-C_4_-H); 9.77 (brs, 1H, OH, D_2_O-exchangeable); 10.41 (s, 1H, NH, D_2_O-exchangeable). Elemental analysis Calcd for C_35_H_31_N_5_O_6_ (617.66): C 68.06, H 5.06, N 11.34. Found: C 68.34, H 5.24, N 11.49.

Compounds **12a–c** were prepared as reported ^21,^^25^.

### General procedure for synthesis of 6-(3-Hydroxyphenyl)-4-(2-oxo-6-substituted-1,2-dihydroquinolin-3-yl)-2-oxo-1,2-dihydropyridine-3-carbonitriles; (13a–c)

A mixture of 3-hydroxyacetophenone (0.136 g, 1 mmol), substituted aldehyde **12a–c** (1 mmol), ethyl cyanoacetate (0.106 ml, 1 mmol) and ammonium acetate (0.616 g, 8 mmol) in 10 ml absolute ethanol was heated under reflux for 4 h. It was then allowed to cool and the obtained solid was filtered, washed with cold ethanol, air dried, and crystallised from ethanol/DMF (9:1) mixture.

#### 6-(3-Hydroxyphenyl)-2-oxo-4-(2-oxo-1,2-dihydroquinolin-3-yl)-1,2-dihydropyridine-3-carbonitrile (13a)

Yield, 45%; m.*p* > 300 °C; IR (KBr, v cm^−1^): 3411 (OH), 3400 (NH), 2221 (C≡N), 1647 (C=O). ^1^H-NMR (400 MHz, DMSO-d_6_, δ ppm): 6.82 (brs, 1H, pyridine-C_5_-H); 6.97 (d, *J* = 8 Hz, 1H, phenyl-C_4_-H); 7.20 (s, 1H, phenyl-C_2_-H); 7.25–7.28 (m, 2H, phenyl-C_5,6_ -H); 7.34 (t, *J* = 7.6, 8 Hz, 1H, quinoline-C_6_-H); 7.39 (d, *J* = 8.4 Hz,1H, quinoline- C_5_-H); 7.62 (t, *J* = 7.6, 8 Hz, 1H, quinoline-C_7_-H); 7.78 (d, *J* = 8 Hz, 1H, quinoline-C_8_-H); 8.30 (s, 1H, quinoline-C_4_-H); 9.84 (s, 1H, OH, D_2_O-exchangeable); 12.22, 12.74 (2 s, each for 1H, 2NH, D_2_O-exchangeable). ^13^C-NMR (100 MHz, DMSO-d_6_δ ppm): 107.50, 114.73, 115.66, 116.50, 118.63, 118.75, 118.97, 122.89, 128.84, 129.27, 130.56. 130.63, 132.31, 134.16, 139.73, 141.11, 156.74, 158.21, 158.27, 159.74, 162.09. Elemental analysis Calcd for C_21_H_13_N_3_O_3_ (355.35): C 70.98, H 3.69, N 11.83. Found: C 70.69, H 3.84, N 11.97.

#### 4-(6-Chloro-2-oxo-1,2-dihydroquinolin-3-yl)-6-(3-hydroxyphenyl)-2-oxo-1,2-dihydropyridine-3-carbonitrile (13b)

Yield, 30%; m.*p* > 300 °C; IR (KBr, v cm^−1^): 3415 (OH), 3304 (NH), 2219 (C≡N), 1647 (C=O). ^1^H-NMR (400 MHz, DMSO-d_6_, δ ppm): 6.85 (s, 1H, pyridine-C_5_-H); 6.98 (t, J = 4,8 Hz, 1H, phenyl-C_4_-H); 7.18–7.28 (m, 2H, phenyl-C_2,6_-H); 7.35 (t, J = 8 Hz, 1H, phenyl-C_5_-H); 7.40 (d, J = 8 Hz,1H, quinoline-C_7_-H); 7.67 (d, J = 8 Hz, 1H, quinoline-C_8_-H); 7.91 (s, 1H, quinoline-C_5_-H); 8.28 (s, 1H, quinoline-C_4_-H); 9.87 (s, 1H, OH, D_2_O-exchangeable); 11.52, 12.37 (2 s, each for 1H, 2NH, D_2_O-exchangeable). Elemental analysis Calcd for C_21_H_12_ClN_3_O_3_ (389.80): C 64.71, H 3.10, N 10.78. Found: C 64.89, H 3.32, N 10.85.

#### 6-(3-Hydroxyphenyl)-4-(6-methoxy-2-oxo-1,2-dihydroquinolin-3-yl)-2-oxo-1,2-dihydropyridine-3-carbonitrile (13c)

Yield, 42%; m.*p* > 300 °C; IR (KBr, v cm^−1^): 3427 (OH), 3320 (NH), 2217 (C≡N), 1645 (C=O). ^1^H-NMR (400 MHz, DMSO-d_6_, δ ppm): 3.81(s,3H, OCH_3_); 6.76 (brs, 1H, pyridine-C_5_-H); 6.96 (d, *J* = 8.4 Hz, 1H, phenyl-C_4_-H); 7.15–7.16 (m, 1H, phenyl-C_6_-H); 7.20–7.24 (m, 1H, phenyl-C_5_-H); 7.28 (dd, *J* = 2.8,8.8 Hz,1H, quinoline- C_7_-H); 7.32 (s, 1H, phenyl-C_2_-H); 7.35 (d, *J* = 8 Hz, 1H, quinoline-C_8_-H); 7.95 (s, 1H, quinoline-C_5_-H); 8.22 (s, 1H, quinoline-C_4_-H); 9.87 (s, 1H, OH, D_2_O-exchangeable); 11.30, 12.15 (2 s, each for 1H, 2NH, D_2_O-exchangeable). EI-MS m/z (% relative abundance): 386 [M^+•^+1] (27.16); 385 [M^+•^] (100); 188 (76.41). Elemental analysis Calcd for C_22_H_15_N_3_O_4_ (385.38): C 68.57, H 3.92, N 10.90. Found: C 68.80, H 3.85, N 10.79.

### General procedure for synthesis of 6-(3-Hydroxyphenyl)-2-mercapto-4-(2-oxo-6-substituted-1,2-dihydroquinolin-3-yl)pyridine-3-carbonitriles; (14a–c)

A mixture of 3-hydroxyacetophenone (0.136 g, 1 mmol), substituted aldehyde **12a–c** (1 mmol), cyanothioacetamide (0.100 g, 1 mmol) and anhydrous ammonium acetate (0.616 g, 8 mmol) in ethanol was stirred and heated under reflux for 4 h. The reaction mixture allowed to cool, and the obtained precipitate was filtered, washed with cold ethanol, air dried and crystallised from ethanol/DMF (9:1) mixture.

#### 6-(3-Hydroxyphenyl)-2-mercapto-4-(2-oxo-1,2-dihydroquinolin-3-yl)pyridine-3-carbonitrile (14a)

Yield, 49%; m.p 278–280 °C; IR (KBr, v cm^−1^): 3500 (OH), 3396 (NH), 2600 (SH), 2219 (C≡N), 1659 (C=O). ^1^H-NMR (400 MHz, DMSO-d_6_, δ ppm): 6.92 (d, *J* = 6.8 Hz, 1H, phenyl-C_4_-H); 7.10 (s, 1H, phenyl-C_2_-H); 7.23–7.28 (m, 2H, phenyl-C_5_,_6_-H); 7.29–7.33 (m, 2H, pyridine-C_5_-H and quinoline-C_6_-H); 7.37 (d, *J* = 8.4 Hz,1H, quinoline- C_5_-H); 7.60 (t, *J* = 7.2, 8 Hz, 1H, quinoline-C_7_-H); 7.76 (d, *J* = 7.6 Hz, 1H, quinoline-C_8_-H); 8.22 (s, 1H, quinoline-C_4_-H); 9.77 (s, 1H, OH, D_2_O-exchangeable); 12.18 (s, 1H, NH, D_2_O-exchangeable); 14.25 (brs, 1H, SH, D_2_O-exchangeable). EI-MS m/z (% relative abundance): 371 [M^+•^] (33.99); 165 (100), 143 (97.90); 97 (93.44). Elemental analysis Calcd for C_21_H_13_N_3_O_2_S (371.41): C 67.91, H 3.53, N 11.31. Found: C 67.69, H 3.75, N 11.48.

#### 4-(6-Chloro-2-oxo-1,2-dihydroquinolin-3-yl)-6-(3-hydroxyphenyl)-2-mercaptopyridine-3-carbonitrile (14b)

Yield, 45%; m.p 227–230 °C; IR (KBr, v cm^−1^): 3550 (OH), 3421 (NH), 2540 (SH), 2211 (C≡N), 1658 (C=O). ^1^H-NMR (400 MHz, DMSO-d_6_, δ ppm): 6.87–6.97 (m, 1H, phenyl-C_4_-H); 7.14–7.50 (m, 4H, phenyl-C_2,5_-H, pyridine-C_5_-H and quinoline-C_5_-H); 7.65–7.76 (m, 1H, quinoline-C_7_-H); 7.90 (d, *J* = 9 Hz, 1H, phenyl-C_6_-H); 8.11–8.36 (m, 2H, quinoline-C_4_-H and quinoline-C_8_-H); 9.69 (s, 1H, OH, D_2_O-exchangeable); 12.39 (s, 1H, NH, D_2_O-exchangeable); 14.23 (s, 1H, SH, D_2_O-exchangeable). Elemental analysis Calcd for C_21_H_12_ClN_3_O_2_S (405.86): C 62.15, H 2.98, N 10.35. Found: C 62.41, H 3.16, N 10.44.

#### 6-(3-Hydroxyphenyl)-2-mercapto-4-(6-methoxy-2-oxo-1,2-dihydroquinolin-3-yl)pyridine-3-carbonitrile (14c)

Yield, 50%; m.p 278–280 °C; IR (KBr, v cm^−1^): 3550 (OH), 3421 (NH), 2590 (SH), 2219 (C≡N), 1663 (C=O). ^1^H-NMR (400 MHz, DMSO-d_6_, δ ppm): 3.81 (s, 3H, OCH_3_); 6.99 (d, *J* = 7.6 Hz, 1H, phenyl-C_4_-H); 7.16–7.18 (m, 2H, phenyl-C_2,6_-H); 7.23 (d, *J* = 8.4 Hz, 1H, quinoline-C_7_-H); 7.27–7.37 (m, 4H, pyridine-C_5_-H, phenyl-C_5_-H and quinoline-C_5,8_-H); 8.25 (s, 1H, quinoline-C_4_-H); 9.91 (s, 1H, OH, D_2_O-exchangeable); 12.18 (s, 1H, NH, D_2_O-exchangeable); 14.17 (brs, 1H, SH, D_2_O-exchangeable). ^13^C-NMR (100 MHz, DMSO-d_6_δ ppm): 56.00, 110.11, 114.16, 115.09, 115.45, 116.93, 117.08, 118.94, 119.44, 119.52, 121.92, 128.97, 130.52, 133.20, 134.39, 140.80, 152.70, 153.21, 154.93, 158.02, 159.24, 179.45. Elemental analysis Calcd for C_22_H_15_N_3_O_3_S (401.44): C 65.82, H 3.77, N 10.47. Found: C 65.70, H 3.98, N 10.60.

### General procedure for synthesis of 2-Amino-6-(3-hydroxyphenyl)-4-(2-oxo-6-substitiuted-1,2-dihydroquinolin-3-yl)pyridine-3-carbonitriles; (15a–c)

An equimolar amount of 3-hydroxyacetophenone (0.136 g, 1 mmol), substituted aldehyde **12a–c** (1 mmol), malononitrile (0.066 g, 1 mmol) and ammonium acetate (0.616 g, 8 mmol) in 10 ml absolute ethanol was heated under reflux for 6 h during which the product was separated out. Then the reaction mixture was cooled, and the yellow precipitate was filtered, washed with cold ethanol, air dried and crystallised from ethanol/DMF (9:1).

#### 2-Amino-6-(3-hydroxyphenyl)-4-(2-oxo-1,2-dihydroquinolin-3-yl)pyridine-3-carbonitrile (15a)

Yield, 50%; m.*p* > 300 °C; IR (KBr, v cm^−1^): 3459 (OH), 3400 (*ν_as_*N-H), 3356 (*ν_s_*N-H), 3244 (NH), 2215 (C≡N), 1651 (C=O). ^1^H-NMR (400 MHz, DMSO-d_6_, δ ppm): 6.88 (d, *J* = 7.6 Hz, 1H, phenyl-C_4_-H); 6.94 (s, 2H, NH_2_, D_2_O-exchangeable); 7.24 (s, 1H, pyridine-C_5_-H); 7.25–7.30 (m, 2H, phenyl-C_5_-H and quinoline-C_6_-H); 7.38 (d, *J* = 8.4 Hz,1H, quinoline- C_5_-H); 7.48–7.52 (m, 2H, phenyl-C_2_-H and quinoline-C_8_-H); 7.59 (t, *J* = 7.6, 8 Hz, 1H, quinoline-C_7_-H); 7.76 (d, *J* = 8 Hz, 1H, phenyl-C_6_-H); 8.20 (s, 1H, quinoline-C_4_-H); 9.61 (s, 1H, OH, D_2_O-exchangeable); 12.16 (s, 1H, NH, D_2_O-exchangeable). ^13^C-NMR (100 MHz, DMSO-d_6_δ ppm): 89.02, 110.64, 114.36, 115.57, 117.13, 117.59, 118.43, 119.21, 122.74, 129.05, 129.99, 130.16, 131.85, 134.49, 139.55, 140.59, 151.32, 158.16, 158.99, 160.21, 160.56. Elemental analysis Calcd for C_21_H_14_N_4_O_2_ (354.37): C 71.18; H 3.98; N 15.81. Found: C 71.49, H 4.06, N 15.95.

#### 2-Amino-4-(6-chloro-2-oxo-1,2-dihydroquinolin-3-yl)-6-(3-hydroxyphenyl)pyridine-3-carbonitrile (15b)

Yield, 45%; m.*p* > 300 °C; IR (KBr, v cm^−1^): 3530(OH), 3418 (*ν_as_*N-H), 3355 (*ν_s_*N-H), 3250 (NH), 2215 (C≡N), 1662 (C=O). ^1^H-NMR (400 MHz, DMSO-d_6_, δ ppm): 6.81 (s, 2H, NH_2_, D_2_O-exchangeable); 6.84 (s, 1H, phenyl-C_2_-H); 6.92 (d, *J* = 9 Hz, 1H, phenyl-C_4_-H); 6.98–7.01 (m, 1H, phenyl-C_5_-H); 7.24–7.50 (m, 3H, pyridine-C_5_-H and quinoline-C_4,5_-H); 7.63 (d, *J* = 8 Hz, 1H, quinoline-C_7_-H); 7.87 (d, *J* = 9 Hz, 1H, phenyl-C_6_-H); 8.18 (d, *J* = 8 Hz, 1H, quinoline-C_8_-H); 9.79 (s, 1H, OH, D_2_O-exchangeable); 12.30 (s, 1H, NH, D_2_O-exchangeable). MS m/z (% relative abundance): 390 [M^+•^+2] (24.76); 388 [M^+•^] (42.93); 315 (100); 63 (76.44). Elemental analysis Calcd for C_21_H_13_ClN_4_O_2_ (388.81): C 64.87, H 3.37, N 14.41. Found: C 64.71, H 3.19, N 14.62.

#### 2-Amino-6-(3-hydroxyphenyl)-4-(6-methoxy-2-oxo-1,2-dihydroquinolin-3-yl)pyridine-3-carbonitrile (15c)

Yield, 53%; m.p 259–260 °C; IR (KBr, v cm^−1^): 3450 (OH), 3380 (*ν_as_*N-H), 3290 (*ν_s_*N-H), 3176 (NH), 2189 (C≡N), 1651 (C=O). ^1^H-NMR (400 MHz, DMSO-d_6_, δ ppm): 3.81 (s, 3H, OCH_3_); 6.88 (d, *J* = 7.6 Hz, 1H, phenyl-C_4_-H); 6.93 (s, 2H, NH_2_, D_2_O-exchangeable); 7.22 (s, 1H, pyridine-C_5_-H); 7.24–7.38 (m, 4H, quinoline-C_5,7_-H and phenyl-C_5,6_-H); 7.48–7.51 (m, 2H, phenyl-C_2_-H and quinoline-C_8_-H); 8.13 (s, 1H, quinoline-C_4_-H); 9.61 (s, 1H, OH, D_2_O-exchangeable); 12.05 (s, 1H, NH, D_2_O-exchangeable). Elemental analysis Calcd for C_22_H_16_N_4_O_3_ (384.40): C 68.74, H 4.20, N 14.58. Found: C 68.95, H 4.32, N 14.67.

## Biological evaluation

All chemicals, solvents, media and kits were purchased from commercial suppliers. Biological evaluation procedures were performed in Medical Biotechnology Department, Genetic Engineering and Biotechnology Research Institute, City of Scientific Research and Technological Applications (SRTA-City), Egypt.

### Cytotoxicity screening

Cytotoxicity of the tested compounds was evaluated using normal human lung fibroblast Wi-38 cell line compared to currently used anticancer drug (doxorubicin) using MTT method was used for assaying cell viability^26^ (Supplementary Page S55).

### Determination of the anticancer activity

Anticancer effect of the above-mentioned compounds was evaluated using four human cancer cell lines. Colon cancer cells (Caco-2), myeloid leukaemia cell line (NFS-60), liver cancer cell line (HepG-2) and prostate cancer cell line (PC-3) using MTT method was used for assaying cell viability^26^ (Supplementary Page S55).

### Flow cytometric analysis of apoptosis

The apoptosis-dependent c active compounds was determined by quantification of annexin-stained apoptotic cells using the FITC signal detector (FL1) against the phycoerythrin emission signal detector (FL2) (Supplementary Page S55).

### Caspase 3/7 activation assay

The percentage of caspase 3/7 activation was quantified using the Apo-ONE ® caspase 3/7 kit following the manufacturer’s instructions^27^ (Supplementary Page S56).

#### Statistical analysis

Data were expressed as mean ± standard error of the mean (SEM). Statistical significance was estimated by the multiple comparisons Tukey post-hoc analysis of variance (ANOVA) using the SPSS16 program^28^ (Supplementary Page S56).

### PIM-1 and PIM-2 kinase inhibitory activity

The most active anticancer compounds were tested for their ability to *in-vitro* inhibit PIM-1 and PIM-2 kinase utilising PIM-1 and PIM-2 Kinase Assay Kit – Promega Corporation catalogue #V4032, following the manufacturer’s instructions^29^.

### Molecular modelling

#### Molecular docking study

The X-ray structures of PIM-1 and PIM-2 enzymes were retrieved from the Protein Data Bank (PDB) with PDB IDs: 2OBJ^18^ and 4X7Q^30^, respectively. The PDB structures were prepared using OpenEye’s MakeReceptor GUI of OEDOCKING 3.5.0.4 of OpenEye Scientific Software^31^^,^^32^ at default levels. Redundant chains, non-essential ions, water molecules and ligands were discarded. The structural water molecule of PIM-1 kinase was kept. The search box around the X-ray co-crystal ligand was set 15.33 Å x 17.00 Å x 19.33 Å (5039 Å^3^) as box dimensions (box volume) for PIM-1 kinase, and 19.00 Å x 16.67 Å x 16.00 Å (5066 Å^3^) for PIM-2 kinase. Site shape potentials were calculated via setting the binding site contours in a balance between the protein and the solvent. The protein files were saved as OEDU format for FRED docking.

The studied structures were built via Marvin Sketch 17.2.6.0 ChemAxon (http://www.chemaxon.com), the lowest energy conformer was generated using MMFF94 Force Field, with normal optimisation limit at default settings. Then different conformations were generated for FRED docking via OpenEye OMEGA conformer generation software^33^^,^^34^. The docking was carried out using OpenEye’s FRED docking software at default levels^31^^,^^32^.

The 3 D and 2 D depictions of the docking poses in PIM-1 and PIM-2 binding sites were generated via Molecular Operating Environment (MOE)^35^.

#### *In-silico* prediction of the pharmacokinetics properties

The *in-silico* predictions of the pharmacokinetics properties were performed using SwissADME^3^,^6^.

## Results and discussion

### Chemistry

**Scheme 1.**
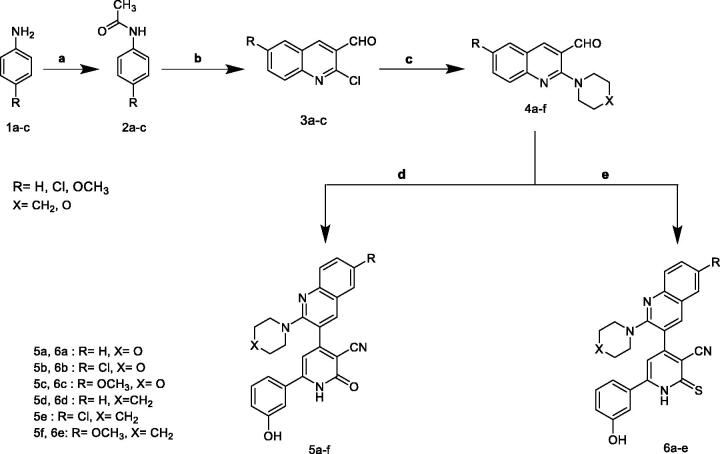
Synthesis of 2-pyridone/2-thioxopyridine – quinoline hybrids. (a) Ac_2_O; (b) POCI_3_/DMF; (c) morpholine or piperdiine/ K_2_CO_3_/DMF; (d) 3-hydroxyacetophenone / ammonium acetate/ CNCH_2_COOC_2_H_5_/ ethanol, reflux; (e) 3-hydroxyacetophenone /ammonium acetate / CNCH_2_CSNH_2_/ ethanol, reflux.

In our concernment for the synthesis of biologically active compounds, the synthetic strategies used for the preparation of the target compounds were outlined in three schemes. We illustrated herein the study for the synthesis of two new series of 4,6-diaryl −2-oxo or thioxo-1,2-dihydropyridine-3-carbonitriles **5a–f** and **6a–f** outlined in Scheme 1. The first step in this work was acetylation of aromatic amines **1a–c** with acetic anhydride gave the corresponding substituted N-phenylacetamides **2a–c**, which after treatment with Vilsmeier’s reagent afforded the substituted 2-chloro-3-formylquinolines **3a–c.** After that, 2-chloro-3-formylquinolines **3a–c** were reacted with morpholine or piperdine to give 2-(morpholine/piperdine-4-yl)quinoline-3-carbaldehyde **4a–f** according to reported reaction conditions^22–24^. 2-(morpholine/piperdine-4-yl)quinoline-3-carbaldehyde **4a–f** was then subjected to one pot reaction with the 3-hydroxyacetophenone and ethyl cyanoacetate or cyanothioacetamide in the presence of excess ammonium acetate to give the corresponding 3-cyanopyridones **5a–f** or 3-cyanothiopyridines **6a–e**, respectively. Structures of the synthesised compounds **5a–f** and **6a–e** were confirmed by elemental microanalysis, IR, ^1^H NMR for all compounds, beside EI/MS for compounds **5b**,**e** and **6b,e** and ^13^C NMR spectra for compound **5b,d** and **6c,e**. The IR spectra of all synthesised compounds showed bands at a stretching frequency (ν) around 3400 cm^−1^ corresponding to the N–H group and around 2200 cm^−1^ corresponding to CN group. The ^1^H-NMR spectra of the pyridone derivatives showed a methine proton as a deshielded singlet peak at the downfield region δ (6.9 − 7.3) ppm while for thiopyridine derivatives appeared around 8.25 ppm. ^13^C-NMR spectra revealed signals for C≡N and a highly deshielded signal corresponding to C=O or C=S. EI-MS of **5b**, **5e,** and **6e** showed [M^+•^] peak at 458, 456, and 468, respectively in addition to [M^+•^+2] peak for **5b** at 460 and for **5e** at 458.

Scheme 1, the obtained results declared that, compounds **5a**, **5b, 5c**, **5d,** and **6e** had the most potent activity against Caco-2 cell line (Figure 5) even superior to doxorubicin. While compounds **5e**, **5f,** and **6b** illustrated nearly equipotent activity. Also, compounds **5b** and **5c** exhibited high sensitivity against PC-3 cell line compared to doxorubicin (Figure 8). Moreover, compounds **5b** and **5c** exerted higher anticancer activity than doxorubicin against HepG-2 cell line. While compound **6e** displayed promising anticancer activity nearly equal to doxorubicin (Figure 6). Furthermore, compound **5b** represented high potency against NFS-60 cell line rather than doxorubicin. Whereas compound **5c** showed nearly equipotent activity (Figure 7). Consequently, compounds **5b** and **5c** exerted the highest anticancer activity against all tested human cancer cell lines, correlated to other compounds and doxorubicin. Indeed, compound **6e** demonstrated higher anticancer activity against Caco-2 and HepG-2 compared to doxorubicin and nearly equipotent activity against NFS-60 and PC-3 cell lines. The selectivity index (SI) was calculated for the tested compounds as shown (Table 1 in Supplementary Material**).** The results indicated that, compounds **5b, 5c,** and **6e** showed the highest SI values. Compound **5c** was found to have the highest SI values against all cancer lines. Consequently, it was the most selective compound towards all cancer cell lines as well as the most non-toxic towards Wi-38. Additionally, morphological changes on the four cancer cell lines (HepG-2, Caco-2, NFS-60, and PC-3) were investigated before and after treatment with the most active and safe compounds **5b, 5c,** and **6e** in comparison with cells treated with the reference doxorubicin. As shown, these compounds caused severe shrinking and collapsing in the normal shape of four studied cancer cell lines as shown in (Figure 9).

**Scheme 2.**
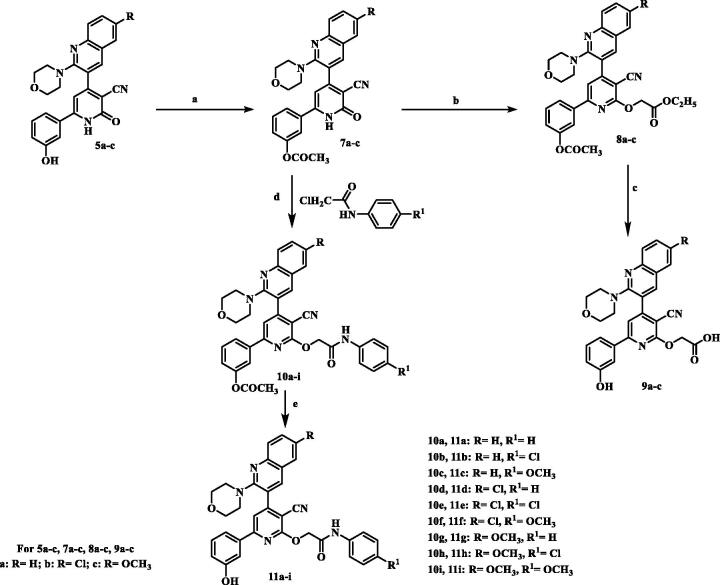
Synthesis of 2-O-substitutedpyridine – quinoline hybrids. (a) Ac_2_O, reflux; (b) BrCH_2_COOC_2_H_5_/K_2_CO_3_, reflux; (c) NaHCO_3_ / reflux; (d) K_2_CO_3_, reflux; (e) NaHCO_3_/ reflux.

The synthesis of 2-O-substitutedpyridine derivatives is very straightforward and demonstrated in Scheme 2. The key intermediates, phenyl acetate derivatives **7a–c**, were synthesised via heating compounds **5a–c** with excess acetic anhydride. IR spectra of newly synthesised compounds **7a–c** displayed absence of the band characteristic to OH function and presence of the absorption band for carbonyl moiety of the acetyl group (COOCH_3_) at 1760–1763 cm^−1^. ^1^H-NMR spectra for compounds **13a–c** revealed presence of shielded singlet signal attributed to CH_3_ protons at a range of δ 2.30–2.31 ppm and in addition, absence of D_2_O exchangeable signal corresponding to OH. ^13^C-NMR spectrum for compound **13c** showed signals assigned for a highly shielded signal corresponding to acetyl CH_3_ at δ 21.29 ppm and highly deshielded signal corresponding to carbonyl group of the ester at δ 174.50 ppm. First alkylation takes place by treatment of intermediated **7a–c** with ethyl bromoacetate in presence of potassium carbonate to give compounds **8a–c**. IR spectra of compounds **8a–c** displayed a characteristic two absorbing bands for two esters C=O at 1750–1780 cm^−1^. ^1^H-NMR spectrum for compound **8a** revealed disappearance of D_2_O exchangeable signal corresponding to NH. In addition, it showed shielded triplet signal integrated for three protons assigned for CH_3_ at δ 1.21 ppm, a singlet signal for two protons attributed to CH_2_ at δ 2.32 ppm and a quartette band integrated for two protons corresponding to CH_2_ at δ 4.20 ppm. Compounds **8a–c** were hydrolysed using sodium bicarbonate in methanol\water (1:1) to produce the acid derivatives **9a–c**. IR spectra of compounds **9a–c** lacked the absorption bands of the carbonyl groups of the starting compounds. Moreover, a broad band characteristic to carboxylic (OH) appeared at 3000–3600 cm^−1^ in addition to an absorption band assigned for acidic carbonyl function observed at 1598–1732 cm^−1^ and an absorbing band for OH function was obtained at 3403–3448 cm^−1^. ^1^H-NMR spectra for compounds **9a–c** revealed absence of triplet and quartette signals corresponding to C_2_H_5_ of ester and singlet attributed to CH_3_ of acetyl group. Instead, spectra showed two D_2_O exchangeable signal resonated at downfield corresponding to 2OH protons at range of δ 9.77–9.84 and 13.10–13.22 ppm. ^13^C-NMR spectrum for compound **9a** showed appearance of highly deshielded signal corresponding to C=O for acid at δ 170.09 ppm. EI/MS for **9b** showed [M^+•^+2] at m/z 518, molecular ion peak [M^+•^] at m/z 516 and base peak at m/z 413. Furthermore, the intermediates **7a–c** were alkylated with N-aryl-2-chloroacetamides in the presence of anhydrous potassium carbonate in dry acetone to yield the intermediates **10a–i** which were further deprotected by sodium bicarbonate in aqueous methanol (1:1) to prepare the final compounds **11a–i**. IR spectra of the target compounds **10a–i** displayed stretching absorption bands for amide NH and C=O functions at 3407–3447 and 1667–1680 cm^−1^, respectively. ^1^H-NMR spectra for compounds **10b, 10f,** and **10g** demonstrated upfield singlet signal corresponding to CH_2_ at a range of δ 5.20–5.23 ppm, and D_2_O exchangeable signal corresponding to NH for amide linkage at a range of δ 10.33–10.61 ppm. While, IR spectra of compounds **11a–i** showed disappearance of a characteristic absorption band due to acetyl C=O function and appearance of a characteristic absorption band due to OH function at 3450–3550 cm^−1^. ^1^H-NMR spectra for compounds **11a–i** demonstrated presence of upfield singlet signal corresponding to CH_3_ of acetyl group. Furthermore, the spectra displayed two D_2_O exchangeable signals corresponding to OH at a range of δ 9.66–9.88 ppm and amide NH at a range of δ 10.26–10.62 ppm. ^13^C-NMR spectra for compounds **11a,c** displayed shielded signal corresponding to OCH_2_ at δ 65.82 ppm and a highly deshielded signal corresponding to C=O at δ 166.47 and 165.99 ppm, respectively. EI/MS for **11e** showed [M^+•^+4] at m/z 630, the molecular ion peak [M^+•^] at m/z 626 and the base peak at m/z 127. EI/MS for **11f** showed [M^+•^+2] at m/z 624, the molecular ion peak [M^+•^] at m/z 622 and the base peak at m/z 182.

**Scheme 3.**
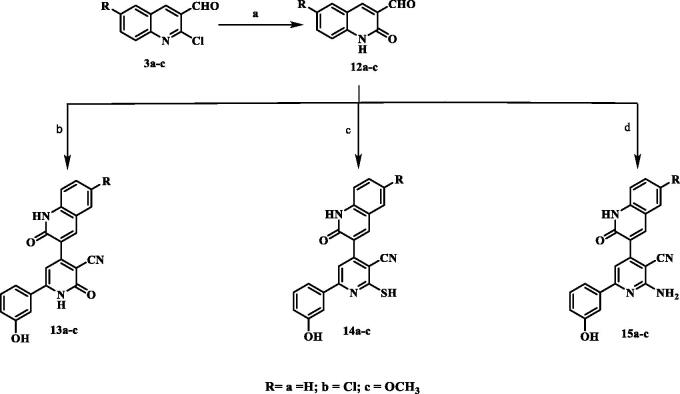
Synthesis of 2-pyridone/2-thiopyridine/2-aminopyridine – quinolone hybrids. (a) CH_3_COOH, reflux; (b) 3-hydroxyacetophenone / ammonium acetate/ CNCH_2_COOC_2_H_5_/ ethanol, reflux; (c) 3-hydroxyacetophenone /ammonium acetate / CNCH_2_CSNH_2_/ ethanol, reflux; (d) 3-hydroxyacetophenone /ammonium acetate / CNCH_2_CN/ ethanol, reflux.

The synthesis of the target pyridine-quinolone hybrids was outlined in Scheme 3. This scheme discussed the chemical pathway used to prepare quinolone aldehydes **12a–c** via hydrolysis of 2-chloro-6-substitutedquinoline-3-carbaldehydes **3a–c** which were prepared by the action of Vilsmeier’s reagent on acetanilide derivatives **2a–c**^25^. Those target quinolone aldehydes **12a–c** were reacted with 3-hydroxyacetophenone, ammonium acetate and ethyl cyanoacetate or cyanothioacetamide or malononitrile to give the corresponding 3-cyanopyridones **13a–c** or 3-cyanothiopyridines **14a–c**, or 3-cyano-2-aminopyridines **15a–c,** respectively. The IR spectra of compounds **13a–c** showed absorption bands at 3304–3400 cm^−1^ attributed to NH, in addition to absorption bands at 2217–2221 cm^−1^ and 1645–1647 cm^−1^ assigned to C≡N and C=O functions, respectively. Moreover, IR of compounds **14a–c** showed characteristic SH band at 2540–2600 cm^−1^. Compounds **15a–c** displayed the presence of two strong bands of asymmetrical and symmetrical stretching frequency of NH_2_ function at 3380–3418 and 3290– 3356 cm-1, respectively. The ^1^H-NMR spectra of the all-synthesised compounds showed a methine proton as a deshielded singlet peak at the downfield region δ 6.76–7.50 ppm. In addition, ^1^H-NMR spectra of compounds **14a–c** showed highly deshielded D_2_O exchangeable SH proton appeared at a range δ 14.17–14.25 ppm which confirm the presence of all derivatives in thiol tautomer, while compounds **15a–c** showed D_2_O singlet for two protons corresponding to NH_2_ protons at δ 6.81–6.94 ppm. ^13^C-NMR spectra showed signals for C≡N and highly deshielded signal corresponding to C=O, C=S or C-NH_2_ determined to be in accordance with the recommended molecule structures. EI-MS of **13c** and **14a** showed [M^+•^] peak at 385 and 371, respectively.

## Biological evaluation

### Cytotoxicity screening

Cytotoxicity test is one of the biological evaluations and screening tests that use human normal and cancer cells *in-vitro* to observe the effect of the studied compounds on their growth using MTT-based cytotoxicity. Microculture MTT method^26^ was used to determine cytotoxic activity of the newly synthesised compounds against normal human lung fibroblast (Wi-38) using doxorubicin (Dox) as standard anticancer drug. The highest IC_50_ and EC_100_ values illustrate that the compounds were safe on the proliferation of normal human cells. All the tested compounds showed significant noncytotoxic effect on human lung fibroblasts (Wi-38) with IC_50_ ranging from 0.0255 to 0.759 µM compared to doxorubicin (IC_50_ = 0.0266 µM). Compounds **5b, 5c, 6a–e, 7a–c, 9a–c, 11a–d, 11f–i, 14a–c,** and **15a–c** showed the highest IC_50_ values > 0.099 µM which revealed their safety on normal human cells (Table 1 in Supplementary Material, Figure 4).

**Figure 4.**
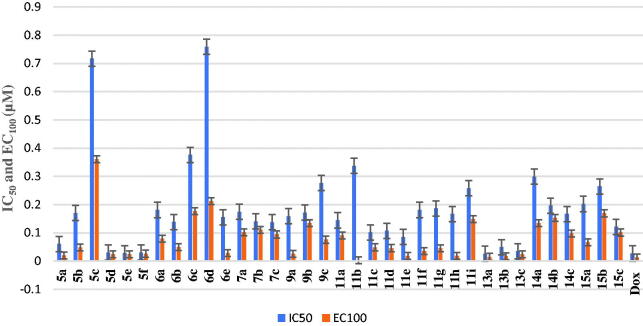
IC_50_ and EC_100_ (μM) of the tested compounds against normal human cells (wi-38).

### *In-vitro* anticancer screening

All final compounds were evaluated against four human cancer cell lines namely; liver cancer (HepG2) (ATCC® HB-8065™), colon cancer (Caco-2) (ATCC^®^ HTB-37™), leukaemia cancer (NFS-60) (ATCC^®^ CRL-1838™), and prostate cancer (PC-3) (ATCC^®^ CRL-1435™) using doxorubicin (Dox) as the reference drug being widely used in tumour management utilising microculture MTT assay, declared by Mosmann^26^ (Table 1 in Supplementary Material, Figures 5–8). The provided data revealed that most of the tested compounds exerted remarkable broad antitumor activity against all tested cancer cell lines.

**Figure 5.**
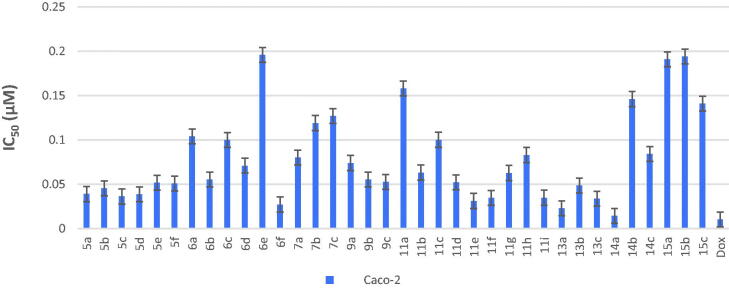
*In vitro* anticancer activity, IC_50_ (μM), of the tested compounds against Caco-2.

**Figure 6.**
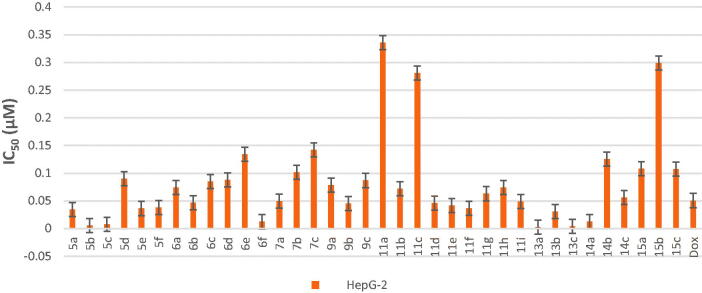
*In vitro* anticancer activity, IC_50_ (μM), of the tested compounds against HepG-2.

**Figure 7.**
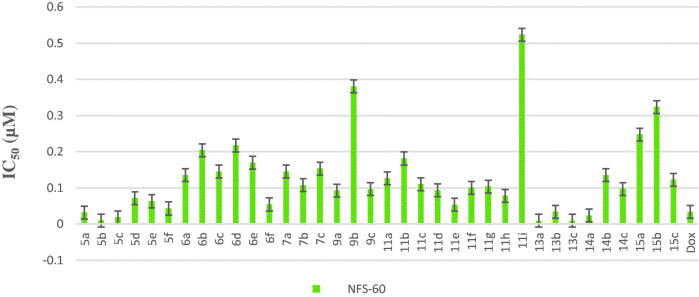
*In vitro* anticancer activity, IC_50_ (μM), of the tested compounds against NFS-60.

**Figure 8.**
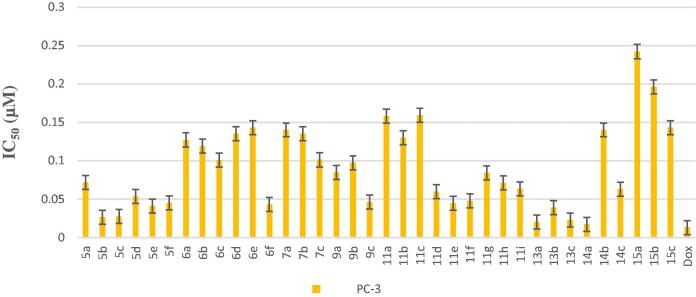
*In vitro* anticancer activity, IC_50_ (μM), of the tested compounds against PC-3.

As a result, it can be concluded that pyridone moiety is important for high anticancer activity Moreover, compounds bearing 4-morpholinyl moiety are more crucial than that having 1-piperdinyl moiety. Therefore, new compounds were designed to keep morpholinyl − 2-oxopyridine scaffold basic structure with various substitutions (Scheme 3) aiming to potentiate the anticancer activity and hence their PIM kinase inhibitory activity. Compounds **7a–c** were designed to acetylate 3-hydroxyphenyl group to study the effect of free OH on the activity. Such structural modification was found to abolish the anticancer activity compared with the hydroxyl derivatives **5a–c** which confirmed the importance of the free hydroxyl group for maintaining high anticancer activity. Our study was excreted to include carboxylic acid moiety on 2-oxopyridine aiming to increase its solubility and hence its bioavailability. Such structural modification doesn’t possess superior anticancer activity. On the other hand, compound **9b** showed low anticancer activity but with equipotent with the reference drug against HepG-2 cell line with IC_50_= 0.0454 µM. The O-(N-aryl acetamide) derivatives **11d**, **11e,** and **11f** showed potent anticancer activity even more potent than doxorubicin against HepG-2 cell line with IC_50_= 0.0458, 0.0416 and 0.0363 µM, respectively (Figure 6). Those compounds showed reasonable anticancer activity against NFS-60, PC-3 and Caco-2 cell lines with IC_50_ < 0.1 µM (Figures 5, 7, and 8). This activity may be due to an increased lipophilicity (6-chloroquinoline) that might enhance the binding pattern to protein hydrophobic moieties. Moreover, compound **11i** showed nearly equipotent activity to the reference drug against HepG-2 cell line with IC_50_= 0.0486 µM. Finally, compounds **11b, 11g,** and **11h** displayed moderate anticancer activity against the four cancer cell lines.

**Figure 9.**
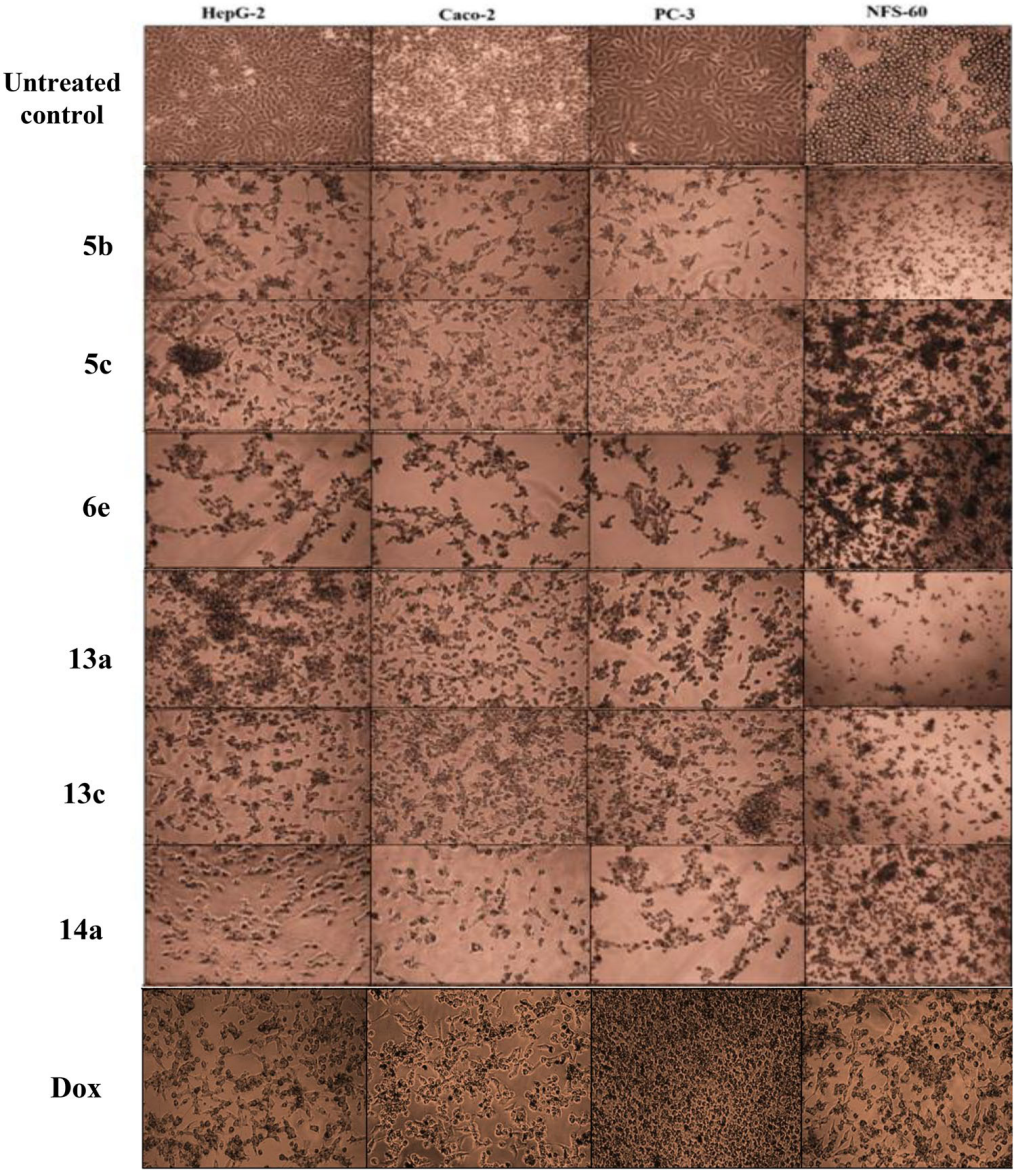
Morphological alterations of the most active compounds-treated cancer cells lines in comparison with the untreated cancer cells.

Another modification was performed through tethering the pyridine moiety with quinolone to study their effect on anticancer activity (Scheme 3). The results indicated that most of the tested compounds **13a–c, 14a–c,** and **15a–c** exerted potent broad antitumor activity against all tested cancer cell lines. Especially, the unsubstituted pyridone derivative **13a** that displayed extraordinary increase in anticancer activity. It was twenty-one-fold more active against HepG-2 cell line (IC_50_=0.00238 μM) and with three-fold against NFS-60 cell line (IC50 = 0.009 μM) compared to the reference (IC_50_=0.0504 and 0.0332 μM, respectively) (Figures 5–8). In addition, 6-methoxy derivative **13c** exhibited thirteen-fold high in the activity against HepG-2 cell line and with three-fold high against NFS-60 cell line. Furthermore, the 6- chloro derivative **13b** was equipotent activity with the reference drug against HepG-2 and NFS-60 cell lines with IC_50_ = 0.0309, 0.03373 μM, respectively, and with pronounced anticancer activity against PC-3 and Caco-2 cell lines. In addition, replacing of 2-oxo group on pyridine ring with 2-mercapto function **14a–c** resulted in slightly decreasing in the anticancer activity. It was found that, the unsubstituted derivative **14a** was the most active one against HepG-2 and NFS-60 cell lines with IC_50_= 0.0127and 0.0232 μM, respectively, and showed moderate activity against PC-3 and Caco-2 cell lines. Moreover, the 6-methoxy derivative **14c** possessed anticancer activity against HepG-2 with IC_50_=0.0557 μM, in a manner that is equipotent to doxorubicin. On the other hand, a dramatic decline in the anticancer activity was recorded with replacement of 2-mercapto group with 2-amino group against the four cancer cell lines. Furthermore, the SI results (Table 1 in Supplementary Material) revealed that, compounds **13a, 13c,** and **14a** showed the highest SI values than the reference drug doxorubicin. They were the least cytotoxic and highest anticancer activity with high SI value compared to the Dox. These results indicated a successful discovery of new anticancer drug candidates and confirmed that they could be safe drugs against tumours. Additionally, morphological changes on the four cancer cell lines (HepG-2, Caco-2, NFS-60 and PC-3) were demonstrated before and after treatment with the most active and safe compounds **13a, 13b,** and **14a** in comparison with cells treated with the reference doxorubicin. those compounds caused severe shrinking and collapsing in the normal shape of four studied cancer cell lines as shown in Figure 9.

### Flow cytometric analysis of apoptosis

**Figure 10.**
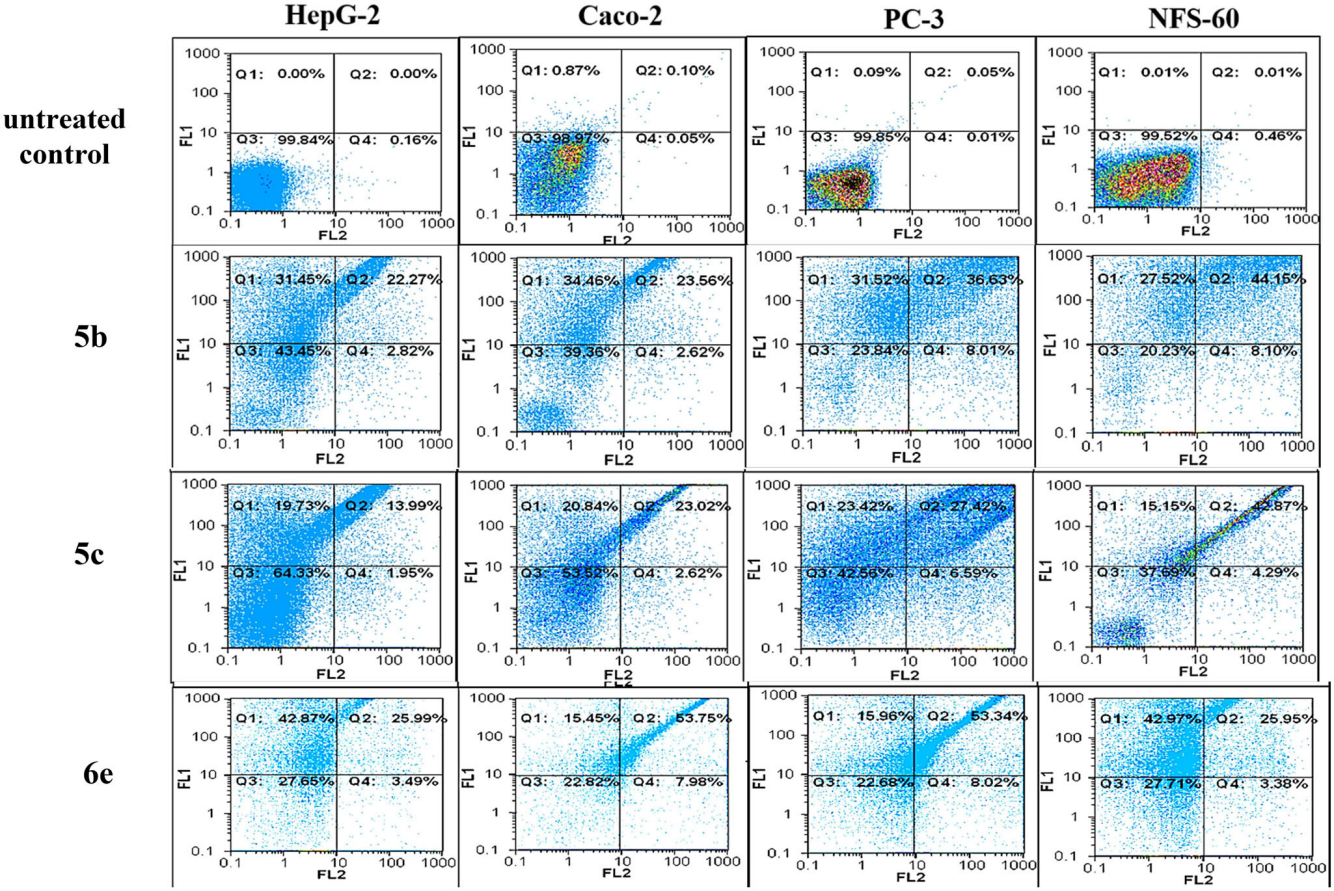
Flow charts of Annexin-PI analysis of **5b**, **5c,** and **6e** – treated cancer cell lines in comparison with the untreated cancer cells.

**Figure 11.**
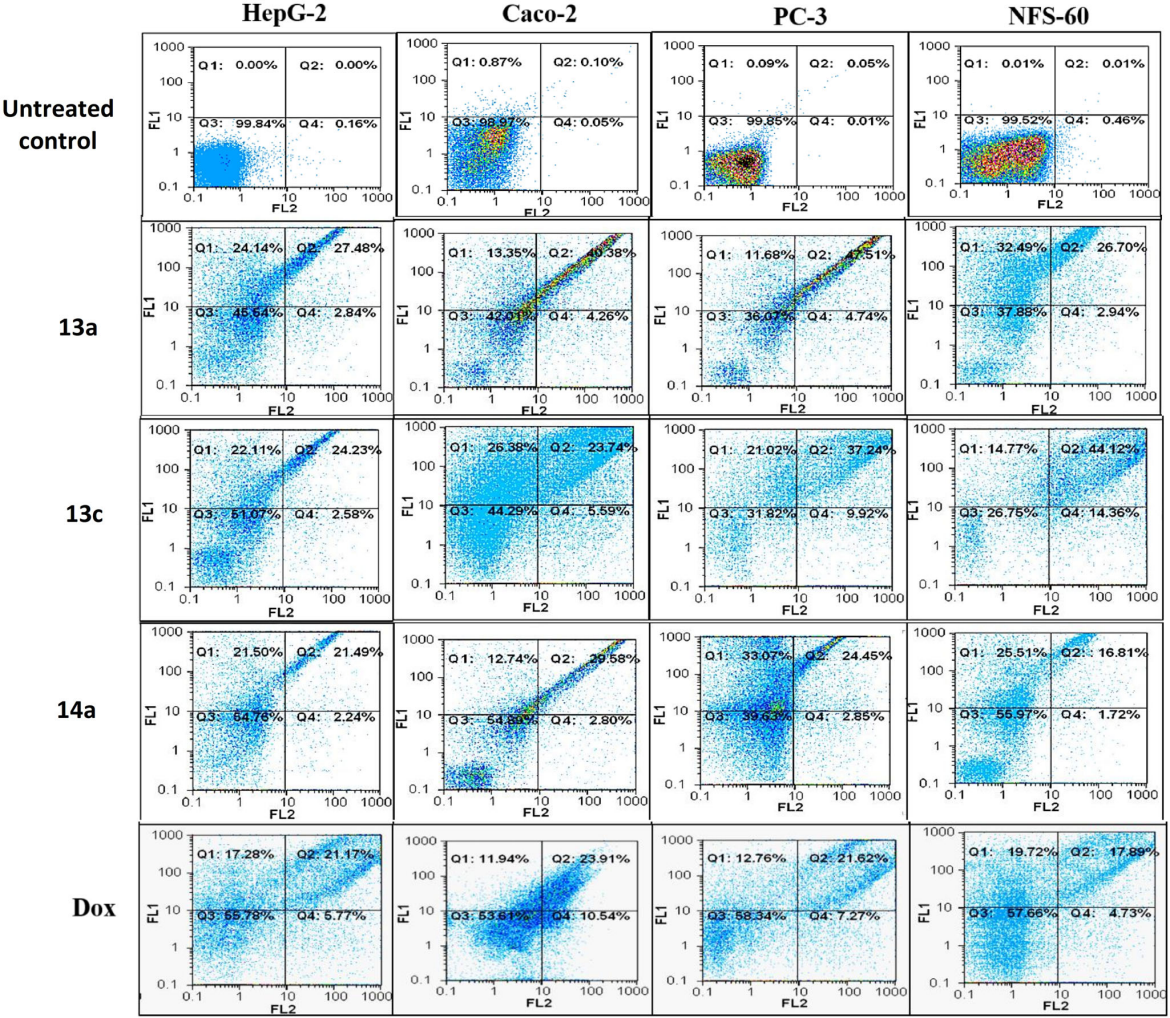
Flow charts of Annexin-PI analysis of **13a**, **13c,** and **14a** – treated cancer cell lines in comparison with the untreated cancer cells.

Flow cytometric annexin V/propidium iodide analysis^37^ was used for detecting the apoptotic effects of the most active and non-toxic anticancer compounds **5b, 5c, 6e, 13a, 13c,** and **14a**. Results (Figures 10 and 11 and Table 1) showed that all tested compounds except **5c** possessed apoptosis-dependent death by over 42% in the treated HepG-2, Caco-2, NFS-60, and PC-3 cancer cell lines higher than doxorubicin which showed less than 39% apoptotic cell. It is worth mentioning that, compounds **6e, 13a,** and **13c** exerted the highest percentage of annexin-stained population cells (>66%) in comparison with the other most effective studied compounds.

**Figure 12.**
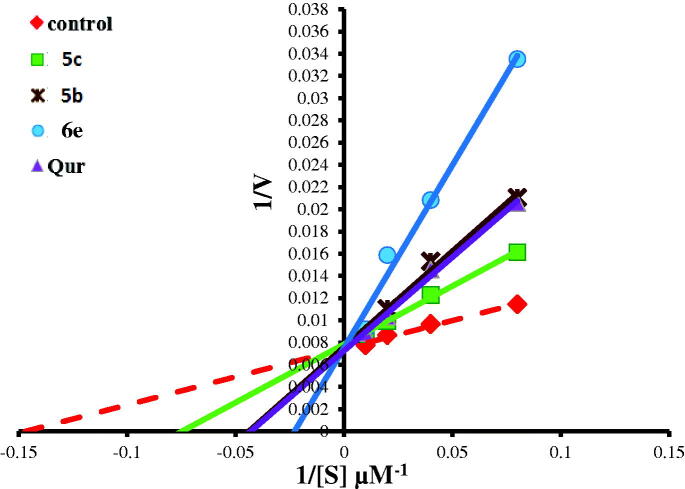
Lineweaver–Burk double-reciprocal plot for PIM kinase inhibition by **5c**, **5b**, and **6e** in comparison with quercetin as reference inhibitor.

**Table 1.** The total percentage of the apoptotic cell population in the most effective compounds-treated cancer cells lines.

Code	HepG-2	Caco-2	PC-3	NFS-60
**Untreated**	0.169 ± 0.02	0.005 ± 0.005	0.505 ± 0.015	0.495 ± 0.005
**5b**	58.9 ± 0.88	44.045 ± 0.185	50.7 ± 0.58	54.2 ± 0.47
**5c**	54.14 ± 0.42	33.89 ± 0.175	47.26 ± 0.92	51.53 ± 0.09
**6e**	69.02 ± 0.18	67.715 ± 0.1.15	68.81 ± 0.49	68.385 ± 0.54
**13a**	70.59 ± 1.07	58.28 ± 0.26	59.71 ± 0.82	59.23 ± 0.04
**13c**	66.83 ± 1.32	51.17 ± 0.33	57.91 ± 0.34	58.83 ± 0.36
**14a**	42.155 ± 0.16	42.17 ± 0.82	56.545 ± 0.98	42.005 ± 0.32
**Dox**	39.265 ± 0.81	36.08 ± 0.23	34.59 ± 0.21	37.89 ± 0.28

*Note:* All values were expressed as mean ± SEM.

### Caspase 3/7 activation assay

It’s a fluorescence assay that measures caspase-3 and −7 activities. Caspase 3/7 activators are well known as apoptotic inducers. In view of this, the apoptotic induction mechanism displayed by the most active compounds **5b, 5c, 6e, 13a, 13c,** and **14a** was explained by estimating the fold increase in caspase 3/7 activation in the treated cancer cell line relative to the untreated HepG-2 cells. Results (Table 2) revealed that compounds **6e** and **13a** exhibited the highest caspase 3/7 up regulation by more than 3 folds. In addition, compounds **5b, 5c**, and **13c** significantly induced caspase 3/7 activation by more than two folds in HepG-2 cancer cell lines.

**Table 2.** Illustrates relative fold increase in caspase activity by the most effective compounds relative to untreated HepG2 cancer cells.

Code	Fold increase in caspase activity
**5b**	2.60 ± 0.031
**5c**	2.21 ± 0.04
**6e**	3.54 ± 0.24
**13a**	3.39 ± 0.03
**13c**	2.90 ± 0.07
**14a**	1.86 ± 0.11
**Dox**	1.61 ± 0.088

*Note:* All values were expressed as mean ± SEM.

### *In-vitro* PIM-1 and PIM-2 kinase inhibitory activity

The active synthesised compounds **5b, 5c,** and **6e**, were evaluated for their PIM-1 and PIM-2 kinase inhibitory activities using Quercetin, as reference drug (Table 3). All compounds showed potent PIM-1 kinase inhibitory activity comparable to the reference quercetin. The PIM-2 kinase inhibitory activity results showed that, compound **5b** showed the highest inhibitory activity among other compounds compared to quercetin. While compound **5c** and **6e** excreted slightly less activity than the reference. Therefore, PIM-1 kinase inhibitory activities were tested for the other active synthesised compounds **13a, 13c,** and **14a** using Quercetin, as reference drug (Table 3). Compounds **13a** and **14a** showed potent PIM-1 kinase inhibitory activity.

**Table 3.** *In-vitro* PIM-1 and PIM-2 kinase inhibition data of the most active compounds.

Code	PIM-1 IC_50_ (µM)	PIM-2 IC_50_ (µM)
**5b**	1.31	0.67
**5c**	1	1.66
**6e**	1.10	1.49
**13a**	1.27	–
**13c**	7.78	–
**14a**	0.385	–
**Quercetin**	1.17	0.61

### Mechanism of PIM-1 kinase inhibition

**Figure 13.**
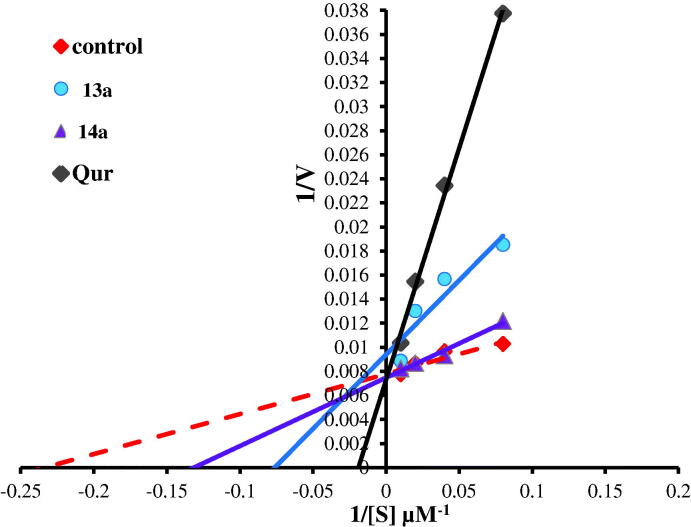
Lineweaver–Burk double-reciprocal plot for PIM kinase inhibition by **13a** and **14a** in comparison with quercetin as reference inhibitor.

To investigate the mechanism of inhibition of the most active compounds **5b, 5c**, **6e, 13a,** and **14a** on PIM-1 kinase activity, enzyme kinetic parameters (maximum activity “V_max_” and substrate concentration at half V_max_ “K_m_”) were assessed by Lineweaver–Burk double-reciprocal plot. As it was shown in this Figures 12 and 13. Lineweaver–Burk double-reciprocal plot for compounds **5b, 5c, 6e**, and **14a** showed the same Y-intercept with the control and quercetin (the same V_max_ in comparison with control while the X-intercept has the negative sign (right to the control) which indicated an increasing in K_m_ value from 7.24 µM (in case of control) to 14.16, 24.69, 27.84, 8.92, and 19.59 µM, for **5b, 5c, 6e, 14a,** and quercetin. respectively. These results indicated that, those compounds are competitive inhibitors for PIM-1 kinase enzyme. In addition, the highest K was recorded in **6e** and **5b** comparing to **5c, 14a** and reference PIM kinase inhibitor (quercetin) indicates the strongest inhibition effect of **6e** and **5b** via lowering the binding affinity of PIM kinase to its substrate by more than three folds. On the other hand, compound **13a** revealed V_max_ at a higher position on the axis (reduced in V_max_ value) and also has the negative sign on the X-intercept (right to control) which indicated increase in K_m_ value therefore, this compound is both competitive and non-competitive inhibitor for PIM-1 kinase enzyme.

**Figure 14.**
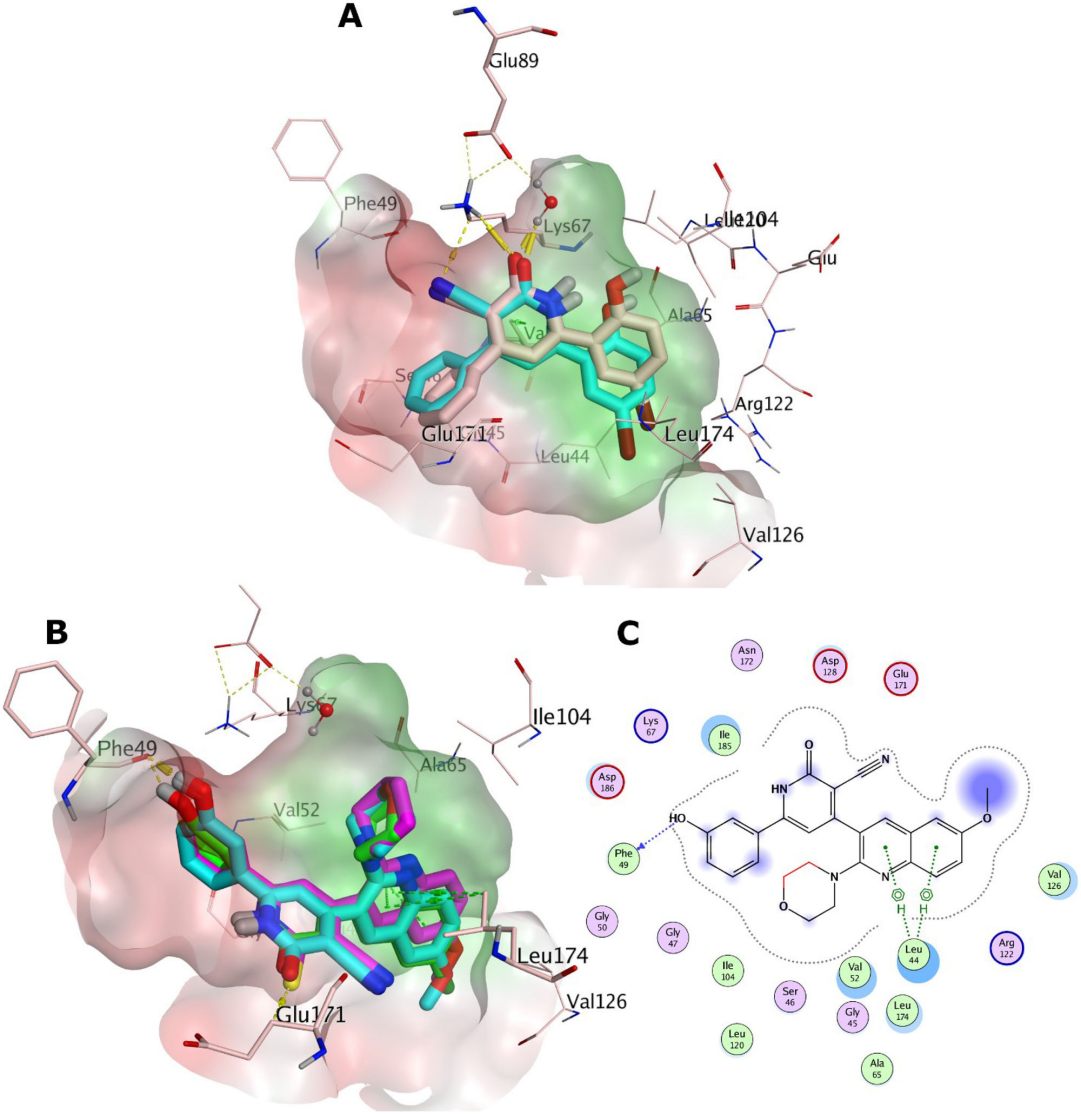
(A) Overlay of the X-ray co-crystal (simon sticks) on its docked pose (cyan sticks) in the binding site of PIM-1 kinase (PDB ID: 2OBJ). (B) Overlay of the docking poses of **5b, 5c,** and **6e** as green, cyan, and magenta sticks, respectively, in the binding site of PIM-1 kinase. (C) Interaction pattern of **5c** with PIM-1 residues in 2D depictions. Polar and non-polar regions of the binding site were presented by red and green coloured molecular surface, respectively. Dashed lines indicate favourable interactions. Non-polar hydrogen atoms were omitted for clarity.

### Structure activity relationship

The obtained results explained that most of the tested compounds exerted remarkable broad antitumor activity against all tested cancer cell lines. Compounds **5a**, **5b, 5c,** and **5d** that contain pyridone moiety hybridised with quinoline derivatives and compound **6e** which comprises thiopyridine moiety showed the most potent activity against Caco-2 cell line superior to doxorubicin which confirm the importance of the presence of 2-oxo or 2-thiopyridine core in the structure. Also, compounds **5b** and **5c** exerted the highest anticancer activity against all tested human cancer cell lines, correlated to other compounds and doxorubicin. Therefore, compounds containing morpholino moiety showed higher activity compared to piperdine-1-yl moiety. Except for, compound **6e** which substituted with 2-piperidine and 6-methoxy group on quinoline ring demonstrated also higher anticancer activity against Caco-2 and HepG-2 than doxorubicin and nearly equipotent activity against NFS-60 and PC-3 cell lines. Finally, substitution at 6-position of quinoline with either lipophilic chloro group or hydrophilic methoxy group showed higher activity rather than unsubstituted one. On the other hand, acetylation of 3-hydroxyphenyl group was found to abolish the anticancer activity compared with the hydroxyl derivatives **5a–c** which confirmed the importance of the free hydroxyl group for maintaining high anticancer activity. Furthermore, SAR study on different alkyl group on 2-oxopyridine revealed that, insertion of N-aryl acetamide showed potent anticancer activity rather than presence of carboxylic acid group which may be due to an increased lipophilicity that might enhance the binding pattern to the enzyme active site by hydrophobic interaction. In addition, linking pyridine to the electron deficient quinolone moiety achieved the goal and exhibited broad *in-vitro* antitumor activity against all tested cancer cell lines. Moreover, pyridone derivatives (**13a** and **13c)** and thiopyridine derivative **14a** showed the highest *in-vitro* anticancer activity against HepG-2 and NFS-60 cell lines superior to reference doxorubicin. Also, compounds **13a** and **14a** revealed potent PIM-1 kinase competitive inhibitory activity. These findings confirmed the importance of substitution at position 2 of pyridine with either oxo or thioxo rather than amino group in anticancer and PIM-1 kinase inhibitory activity. Besides, substitution at position 6 of quinolone with hydrophilic methoxy group or unsubstituted derivatives showed the highest activity rather than the hydrophobic chloro derivatives which could be due to increased polar interaction between the quinolone and PIM-1 kinase hinge region.

## Molecular modelling

### Optimisation methods

#### Ligand efficiency (LE)

A correlation between the potency of a compound (IC_50_) and the number of its heavy atoms (non-hydrogen atoms) is called Ligand Efficiency. It is a measurement of the binding energy which related to the potency of the compound (pIC_50_) with consider its molecular size^38^. The suggested LE value for lead-likeness should be in the range of 0.3 while the acceptable LE value for drug-likeness should be higher than 0.3 (Table 4).

**Table 4.** LE and LLE values for the most active anticancer compounds against the four cancer cell lines (HepG-2, Caco-2, PC-3, NFS-60).

Code	HepG-2		Caco-2		PC-3		NFS-60	
	LE	LLE	LE	LLE	LE	LLE	LE	LLE
**5b**	0.34	4.22	0.30	3.28	0.31	3.52	0.33	3.97
**5c**	0.33	4.68	0.30	4.00	0.30	4.12	0.31	4.30
**6e**	0.32	3.07	0.30	2.73	0.30	2.53	0.29	2.43
**13a**	0.44	6.29	0.39	5.31	0.39	5.37	0.41	5.71
**13c**	0.40	6.06	0.35	5.11	0.36	5.29	0.38	5.69
**14a**	0.40	5.23	0.40	5.18	0.39	5.10	0.39	4.96

#### Ligand lipophilic efficiency (LLE)

LLE is a criterion that evaluate the potency in terms of lipophilicity. In other words, it measures the binding of a ligand to a given target in respect to its lipophilicity^38^. LLE value should be ≥ 3 for lead compound to be acceptable and recommended to be LLE value ≥ 5 for drug like candidate (Table 4).

According to the results, compounds **5b and 5c** obeyed acceptable ranges of LE (>0.3) and LLE values (> 3). Therefore, these compounds fulfilled the criteria of lipophilicity and ligand efficacy to be lead-like compounds. On the other hand, compounds **13a**, **13c,** and **14a** showed drug like criteria (LE >0.3, LLE >5). Therefore, these compounds could impart improved potency along with convenient lipophilicity which make these compounds promising drug-like candidates in anticancer drug discovery.

### Docking with PIM-1 and PIM-2 kinases

The aim of this section in our study is to provide *in-silico* insights to rationalise the observed *in-vitro* PIM-1 and PIM-2 inhibition activity based on our design approach.

For this we performed molecular docking experiments utilising the high-resolution X-ray structures for PIM-1 and PIM-2 kinases, namely, PDB IDs: 2OBJ and 4X7Q, respectively. To validate the docking setup, a pose-retrieval docking experiment was conducted for the co-crystal ligand against its respective X-ray coordinates. All pose-retrieval experiments were successful (Figures 14A and 16(A) with RMSD value < 2 Å indicating high docking accuracy for the docking setup for pose prediction purposes.

**Figure 15.**
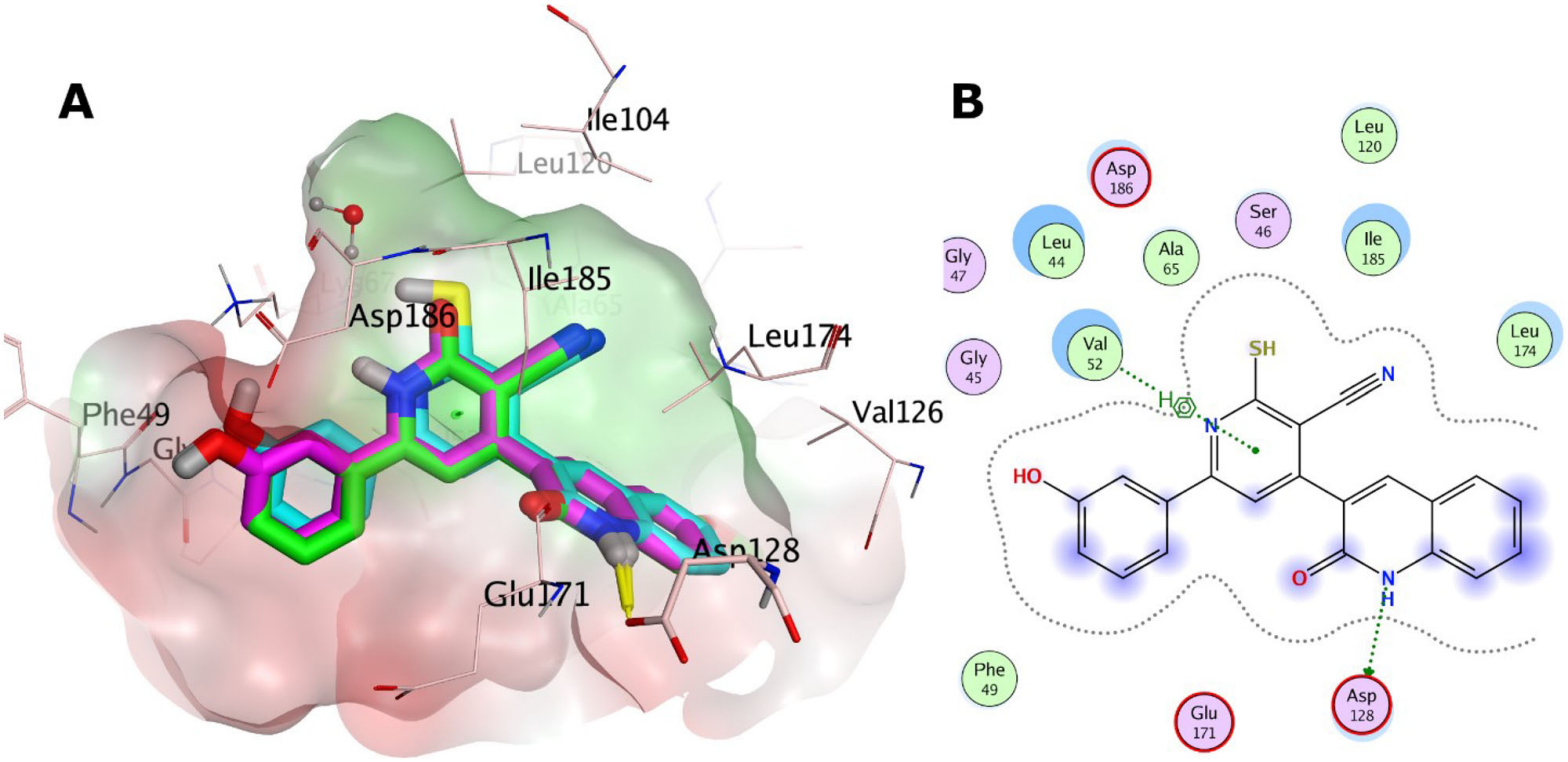
(A) Overlay of the docking poses of **13a, 13c,** and **14a** as magenta, green, and cyan sticks, respectively, in the binding site of PIM-1 kinase. (B) Interaction pattern of **13a, 13c,** and **14a** with PIM-1 residues in 2D depictions. Polar and non-polar regions of the binding site were presented by red and green coloured molecular surface, respectively. Dashed lines indicate favourable interactions. Non-polar hydrogen atoms were omitted for clarity.

Concerning PIM-1 kinase, the docking score distribution (Table 5) exhibits narrow range of scores for **5b**, **5c,** and **6e**. This indicates comparable *in-silico* affinity which came in coherence with the observed *in-vitro* inhibitory activities (IC_50_ range: 1 − 1.31 µM) against PIM-1 kinase. However, the score distribution of the series of **13a**, **13c,** and **14a** displayed superior scores compared to the **5** and **6** candidates, as well as the reference quercetin. One explanation for this is the topological differences between both series that affects pose orientations (as discussed later). It is obvious that **14a** exhibited the best score among all compounds which agrees with the observed *in-vitro* inhibitory activities (**14a** is the best active one).

**Table 5.** The docking score distribution of **5b**, **5c**, **6e**, **13**a, **13c**, **14a** and **Quercetin** against PIM-1 and PIM-2 kinases.

Code	PIM-1	PIM-2
**5b**	−9.43^a^	−6.91
**5c**	−9.43	−5.04
**6e**	−9.85	−5.2
**13a**	−12.73	ND^b^
**13c**	−11.37	ND
**14a**	−13.81	ND
**Quercetin**	−10.33	−11.92

^a^The docking score of FRED.

^b^ND stands for not determined.

Regarding PIM-2 kinase, the docking score of **5b** and **Quercetin** indicate superior *in-silico* binding compared to **5c** and **6e**, as shown in Table 5. Again, this observation is in consistency with the measured *in-vitro* data where both **5b** and **Quercetin** are the most active against PIM-2 kinase with a sub-micromolar range of activity compared to **5c** and **6e** (low micromolar range). It is worth mentioning that **5c**, **6e,** and **Quercetin** (except **5b**) display superior docking score for PIM-1 compared to PIM-2 rationalising their observed *in-vitro* activities. The docking experiments for **13a**, **13c,** and **14a** against PIM-2 were omitted due to the lack of PIM-2 inhibitory activity data.

Based on our analysis on the crystal PIM-1 protein structure, a structural water molecule appears to mediate a H-bonding bonding interaction of the pyridone scaffold of the co-crystal ligand with the side chain of Glu89. Therefore, we kept the structural water molecule in our docking setup. The pose retrieval of the co-crystal ligand was successful implying an acceptable accuracy of the docking setup.

Although both the co-crystal ligand and the selected candidates (**5b**, **5c,** and **6e**) share pyridone/thiopyridine moiety, the docking pose of the core pyridone scaffold of the selected candidates showed a flip towards Glu171. This is attributable to the topology difference between the co-crystal ligand which is 4,6-diaryl pyridone derivative and our quinolinyl – pyridone/thiopyridine hybrids.

Elucidating the docking poses of **5b**, **5c,** and **6e,** they all produce comparable interactions in the binding site of PIM-1 kinase, as seen in Figure 14B. For instance, the core pyridone/thiopyridine demonstrate a favourable hydrophobic interaction with Glu171. Besides, the hydroxyphenyl group shows H-bonding interaction with the backbone of Phe49, e.g. as seen in Figure 14C for **5c**. The methoxy/chloro quinoline moiety is well packed in the hydrophobic area (green area in Figure 14) indicating hydrophobic interactions with Leu174 and Leu44. Interestingly, the morpholine/piperidine moiety appeared to be partially included in a hydrophobic interaction with Ile104 and Ala45, and partially solvent-exposed. Overall, these findings exhibited comparable docking score for **5b**, **5c,** and **6e**, as shown in Table 5. These results are consistent with observed *in-vitro* inhibitory activity against PIM-1 kinase since these compounds exhibited comparable IC_50_ values.

On the other hand, the postulated binding poses of the hybrids of pyridone/thiopyridine with 2-quinolone derivatives, namely **13a**, **13c,** and **14a,** displayed flipped poses compared to **5** and **6** candidates, as exhibited in Figure 15. This is attributable to the differences of topology and substitution pattern between both series. For instance, unlike **5b, 5c,** and **6e** poses, the pyridone/thiopyridine poses of **13a**, **13c,** and **14a** point towards the structural water molecule (Figure 15A). This orientation enables the 2-quinolone moiety to have the advantage of making H-bonding interactions with the side chain of Asp128, and to be packed between the hydrophobic side chains of Val126 and Leu174 implying favourable hydrophobic interactions (Figure 15A,B). Likewise, the pyridone/thiopyridine moiety of **13a**, **13c,** and **14a** is packed between the hydrophobic side chains of Val52 and Ile185. Overall, this postulated orientation produced superior *in-silico* binding particularly for **13a** and **14a**, as observed in Table 5.

**Figure 16.**
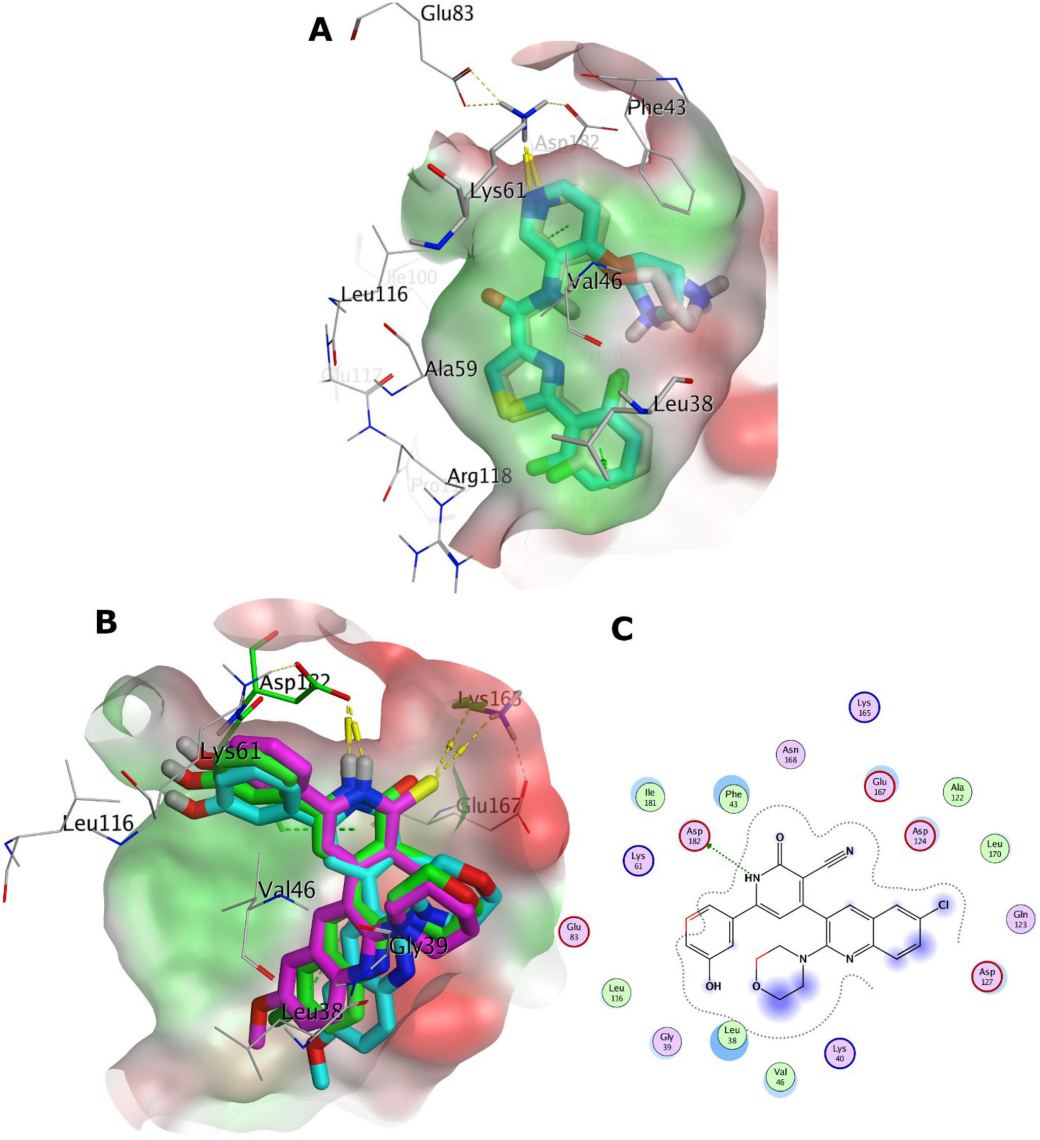
(A) Overlay of the X-ray co-crystal (grey sticks) on its docked pose (cyan sticks) in the binding site of PIM-2 kinase (PDB ID: 4X7Q). (B) Overlay of the docking pose of **5b, 5c,** and **6e** as green, cyan, and magenta sticks, respectively, in the binding site of PIM-2 kinase. (C) Interaction pattern of **5b** with PIM-2 residues in 2D depictions. Polar and non-polar regions of the binding site were presented by red and green coloured molecular surface, respectively. Dashed lines indicate favourable interactions. Non-polar hydrogen atoms were omitted for clarity.

The postulated binding pose of **5b** illustrates favourable interactions in the binding site of PIM-2 kinase as shown in Figure 16(B,C). The pyridone scaffold demonstrates H-bonding interaction with the side chain of Asp182 and hydrophobic interaction with the side chain of Ile181. The chloroquinoline moiety is packed in the hydrophobic area (green area in Figure 16B) formed by Leu38, Ala59, Val46 and Asp124, while the morpholine moiety is directed towards the polar area (red area in Figure 16B) formed by the side chains of Glu167 and Lys40. The hydroxyphenol group appeared to show hydrophobic interactions with the side chains of Val46. Like **5b**, the docking poses of **5c** and **6e** showed similar interactions in the binding site of PIM-2 kinase. Interestingly, the chloroquinoline moiety of **5b** appeared to be well packed in the hydrophobic area avoiding the possible steric bumps of the bigger methoxyquinoline moiety of the congeneric **5c** and **6e.** Globally, these observations contribute to the superior score of **5b** towards the binding site of PIM-2 kinase compared to **5c** and **6e**.

### *In-silico* physicochemical predictions

The purpose of this section is to deliver hints about the drug-likeness and pharmacokinetic properties of the active compounds **5b, 5c,** and **6e.** Explicating these properties can be supportive in the context of medicinal chemistry. Typically, a molecule that fails to fulfil the drug-likeness standards also fail as a clinical candidate due to poor bioavailability, adverse effects or other concerns. We employed the automated SwissADME^36^ for the pharmacokinetics and drug-likeness predictions. The evaluations against passive human gastrointestinal absorption (GIA) and blood-brain barrier (BBB) permeation were extracted from the BOILED-Egg model^36^^,^^39^, which represent some parameters for the pharmacokinetics predictions. The compounds presented high gastrointestinal absorption except **6e** and **14a**, as seen in Table 6. Also, these molecules were suggested to fail to permeate through BBB, and hence, they are expected to show low incidence for central nervous system (CNS) adverse effects. Permeability glycoprotein (P-gp) is proposed to be the most important member among ATP-binding cassette transporters or ABC-transporters^36^ since it is part of the resistance mechanisms against drugs^36^^,^^40^. **5b**, **5c,** and **6e** molecules were foreseen to be substrate for the P-gp which introduce a chance of being resistant against the human biological membranes. However, **13a**, **13c,** and **14a** were predicted to escape the P-gp indicating low incidence of developing resistance against human biological membranes.

**Table 6.** In-silico predictions of the pharmacokinetics and drug-likeness properties for **5b**, **5c**, **6e**, **13a**, **13c** and **14a**.

Code	MW	GIA	BBB	LogP	Lipinski #violations	Veber #violations	PAINS #alerts
**5b**	458.9	High	No	3.57	0	0	0
**5c**	454.5	High	No	2.91	0	0	0
**6e**	468.6	Low	No	4.73	0	0	0
**13a**	355.4	High	No	2.01	0	0	0
**13c**	385.4	High	No	1.98	0	0	0
**14a**	371.4	Low	No	3.41	0	0	0
Code	Pgp substrate	CYP1A2 inhibitor	CYP2C19 inhibitor	CYP2C9 inhibitor	CYP2D6 inhibitor	CYP3A4 inhibitor	
**5b**	Yes	No	Yes	Yes	No	No	
**5c**	Yes	No	No	Yes	No	Yes	
**6e**	Yes	No	Yes	Yes	No	No	
**13a**	No	No	No	No	No	No	
**13c**	No	No	No	Yes	No	No	
**14a**	No	Yes	No	Yes	Yes	Yes	

*Notes:* GIA: human gastrointestinal absorption; BBB: blood-brain barrier permeation; P-gp: permeability glycoprotein; CYP1A2, CYP2C19, CYP2C9, CYP2D6 and CYP3A4 are the five major isoforms of cytochromes P450 (CYP). LogP is calculated as XLOGP3 descriptor^41^. Lipinski #violations counts the number of violations of Lipinski rule summarised as: lipophilicity (logP) ≤ 5, molecular weight ≤ 500, number of hydrogen bond donors ≤ 5 and number of hydrogen bond acceptors ≤ 10. Veber #violations counts the number of violations of Veber rule summarised as: NRB ≤ 10 and TPSA ≤ 140 Å^2^. PAINS #alerts counts the number of pan-assay interference compounds/substructures. All calculations were performed using SwissADME^36^.

Commonly, 50–90% of therapeutic molecules are judged to be a substrate of at least one of the five major isoforms of Cytochrome P (CYP) enzymes (CYP1A2, CYP2C19, CYP2C9, CYP2D6, and CYP3A4)^36^^,^^42^^,^^43^. Inhibition of these isoenzymes is unquestionably one cause of pharmacokinetics-related drug-drug interactions leading to toxic or adverse effects^44^^,^^45^. As shown in Table 6, all molecules were predicted to display inhibition of at least one of the CYP isoforms, with exception of **13a**. This recommends administering these candidates in a sole regime and not in combination with other therapeutic agents. Fortunately, all molecules showed no alert to be a possible PAINS (pan-assay interference compounds)^46^, as shown in Table 6. This stresses that their chemical structures would not interfere in protein assays denoting the *in-vitro* results to be robust with minimum artefacts. Lastly, drug-likeness properties underlined that all molecules do not display violations for the Lipinski and Veber rules^47^ highlighting that these candidates would demonstrate good bioavailability profiles.

## Conclusion

New pyridine-quinoline hybrids were designed and synthesised as PIM-1 kinase inhibitors. They were evaluated against four human cancer cell lines namely; HepG2, Caco-2, NFS-60, and PC-3 using Dox as the reference. Compounds **5b, 5c, 13a, 13c,** and **14a** showed the highest anticancer activity against all tested human cancer cell lines, correlated to other compounds and doxorubicin. Also compound **6e** demonstrated higher anticancer activity against Caco-2 and HepG-2 compared to Dox and nearly equipotent activity against NFS-60 and PC-3 cell lines. On the other hand, flow cytometric annexin V/propidium iodide analysis observed that, compound **6e** revealed the highest percentage of annexin-stained population cells (>67%) in the four tested cancer cell lines in comparison with the other most effective studied compounds. In addition, caspase 3/7 activation assay elicited that, all tested compounds significantly induced caspase 3/7 activation in HepG-2 cancer cell lines. Furthermore, compounds **5b, 5c**, **6e, 13a,** and **14a** showed potent PIM-1 kinase inhibitory activity comparable to the reference quercetin. Besides, kinetic studies using Lineweaver–Burk double-reciprocal plot for the most active compounds on PIM-1 kinase proved that, compounds **5b, 5c**, **6e**, and **14a** and quercetin behaved as competitive inhibitors for PIM-1 kinase. While, compound **13a** was both competitive and non-competitive inhibitor of PIM-1 kinase enzyme. In addition, molecular modelling study indicated that, compounds **5b** and **5c** fulfilled the criteria of lipophilicity and ligand efficacy to be lead-like compounds. Moreover, drug-likeness properties underlined that all molecules do not display violations for the Lipinski and Veber rules highlighting that these candidates would demonstrate good bioavailability profiles. Molecular docking studies indicated that, *in-silico* affinity came in coherence with the observed *in-vitro* inhibitory activities against PIM-1/2 kinases.

## Supplementary Material

Supplemental Material
