# Supplementary material for: Discovery of new pyridine-quinoline hybrids as competitive and non-competitive PIM-1 kinase inhibitors with apoptosis induction and caspase 3/7 activation capabilities
Source: J Enzyme Inhib Med Chem. 2023 Jan 11;38(1):2152810. doi: 10.1080/14756366.2022.2152810 (PMC9848351; doi:10.1080/14756366.2022.2152810)
Supplement: Supplemental Material [file IENZ_A_2152810_SM9547.pdf]

Discovery of New Pyridine-Quinoline Hybrids as Competitive and Non-competitive PIM-1 Kinase Inhibitors with Apoptosis Induction and caspase 3/7 activation capabilities

Mostafa M. M. El-Miligy[1]*, Marwa E. Abdelaziz[1]*, Salwa M. Fahmy[1], Tamer M. Ibrahim[3], Marwa M. Abu-Serie[2], Mona A. Mahran[1], Aly A. Hazzaa[1]

[1]Pharmaceutical Chemistry Department, Faculty of Pharmacy, Alexandria University, Alexandria 21521, Egypt

[2] Medical Biotechnology Department, Genetic Engineering and Biotechnology Research Institute (GEBRI), City of Scientific Research and Technological Applications (SRTA-City), Alexandria 21934, Egypt

[3]Department of Pharmaceutical Chemistry, Faculty of Pharmacy, Kafrelsheikh University, Kafrelsheikh 33516, Egypt

Scheme 1:

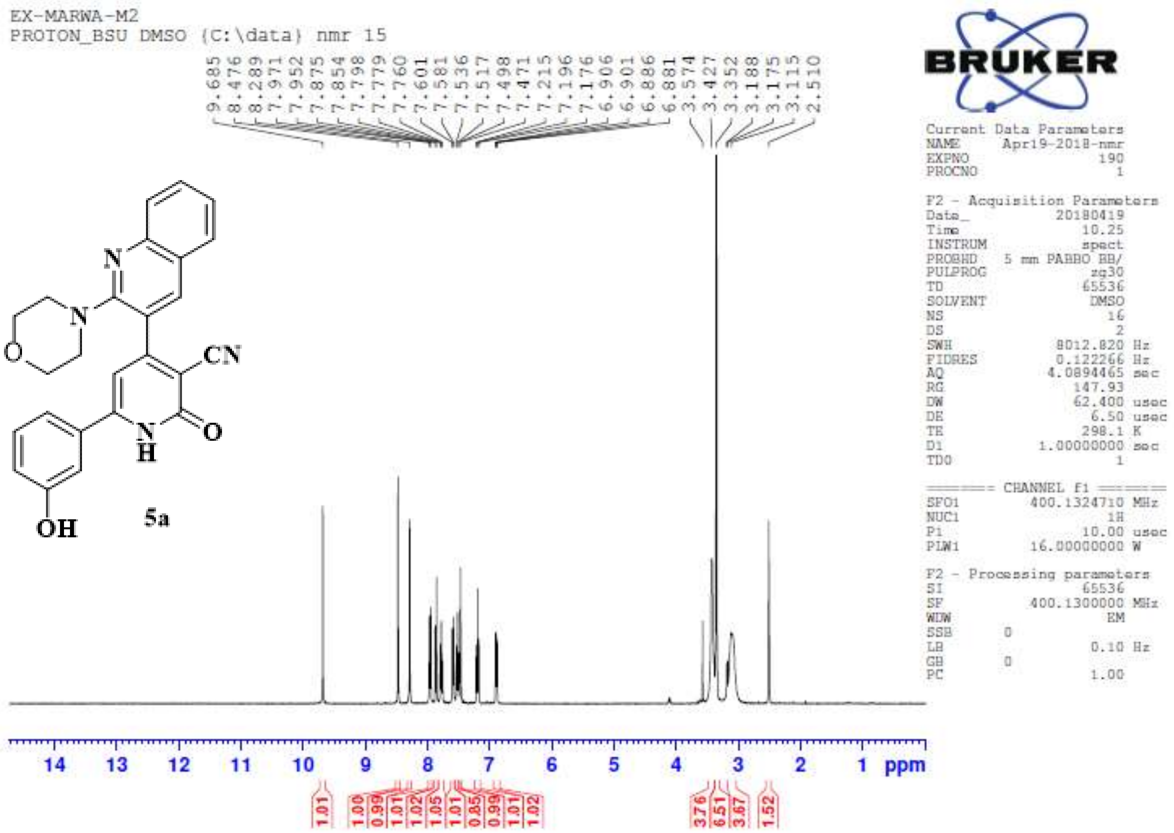

H[1]-NMR spectra of compound 5a

Information Classification: General

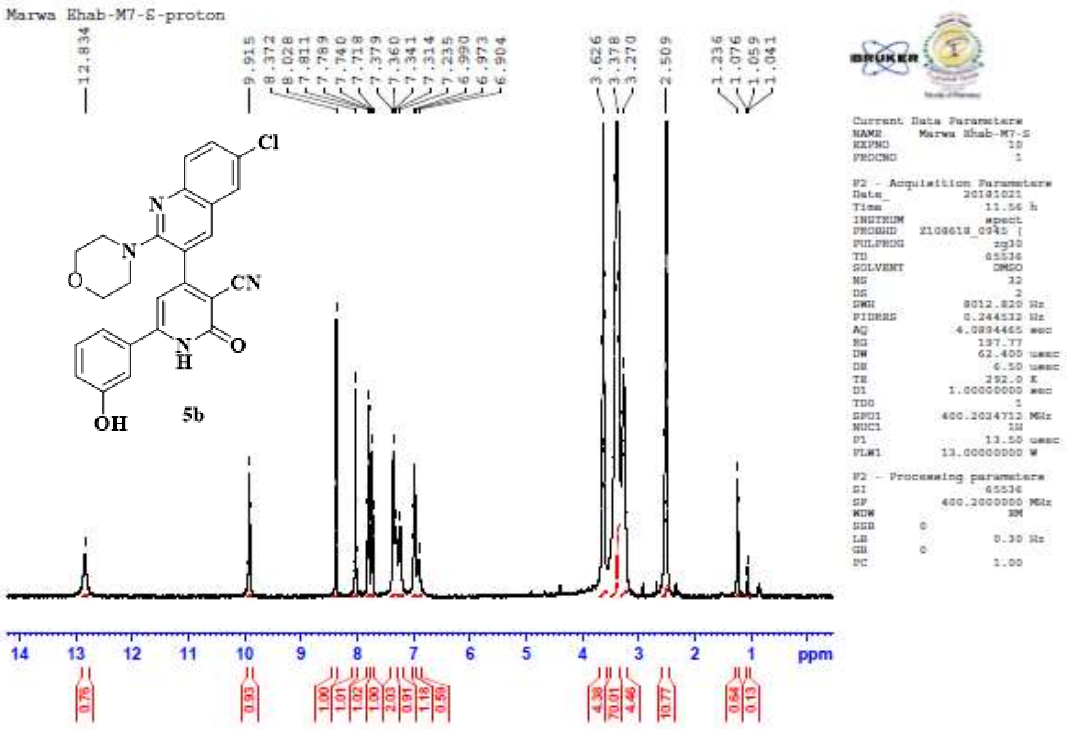

H[1]-NMR spectra of compound 5b

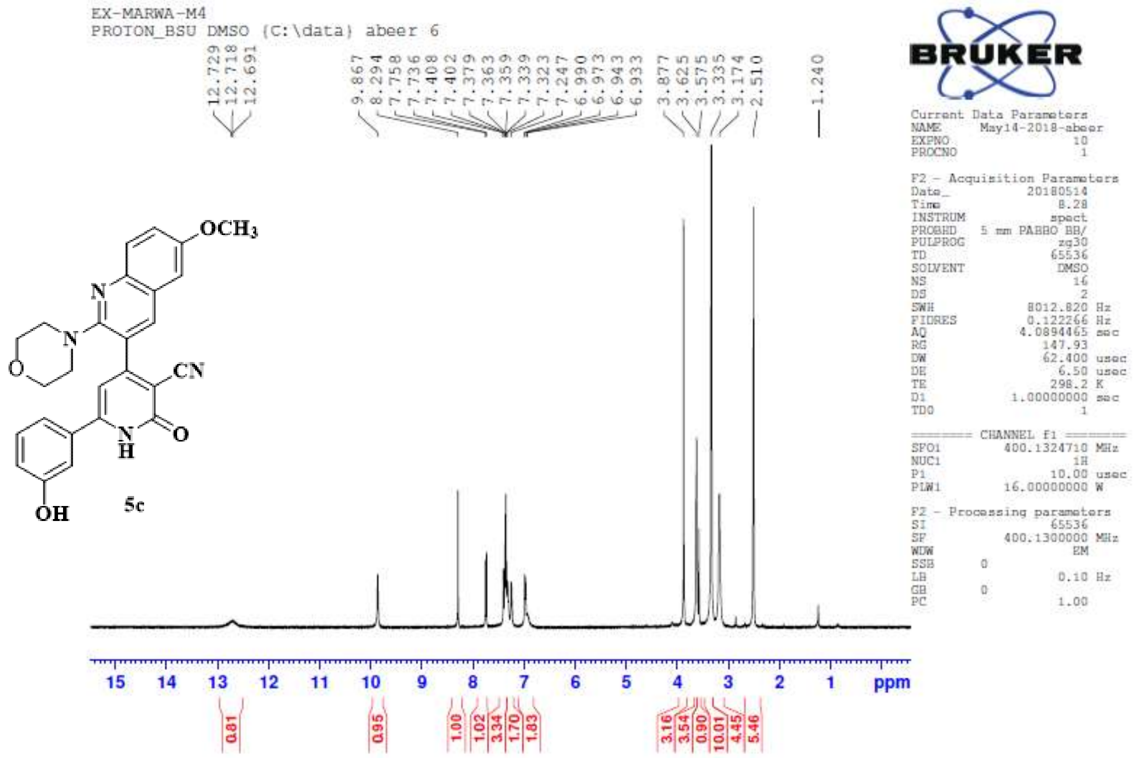

H[1]-NMR spectra of compound 5c

Information Classification: General

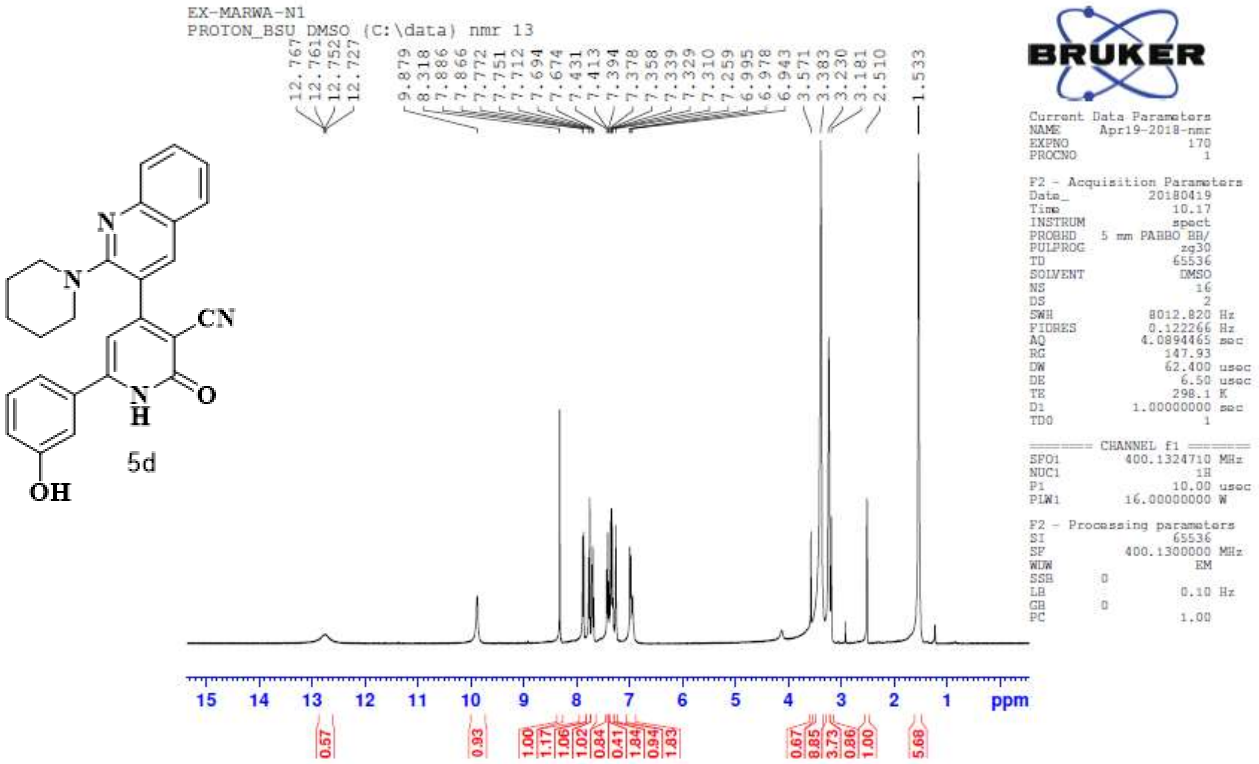

H1-NMR spectra of compound 5d

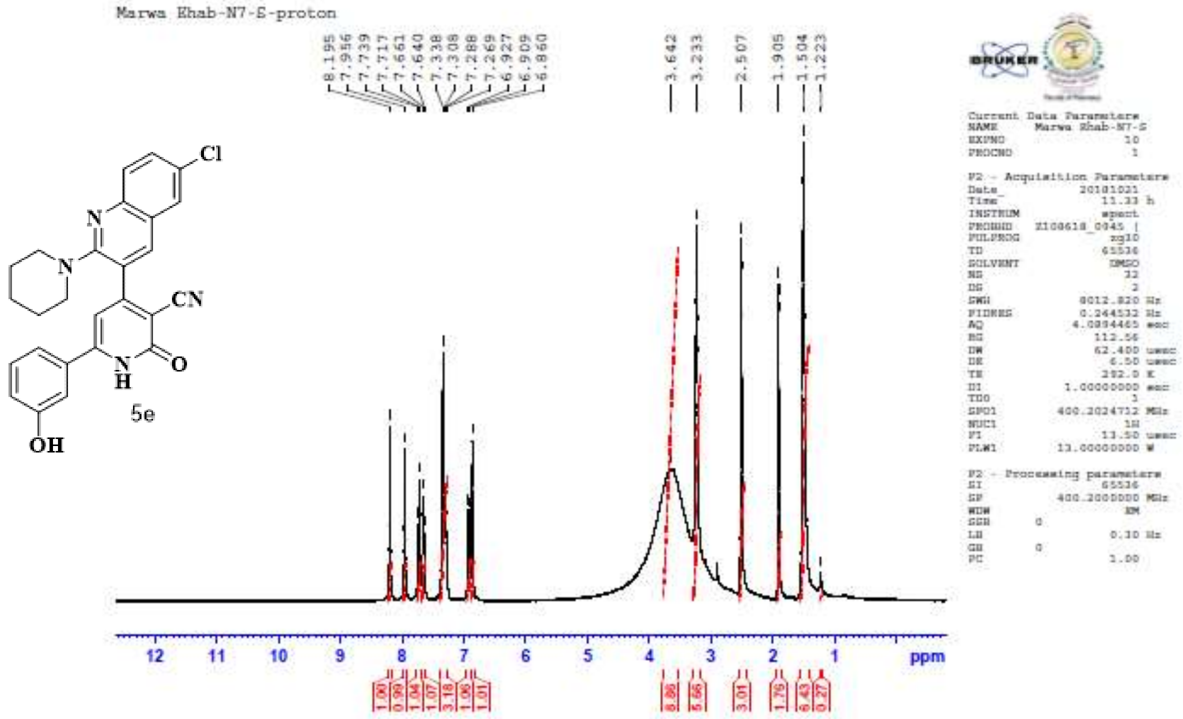

H1-NMR spectra of compound 5e

Information Classification: General

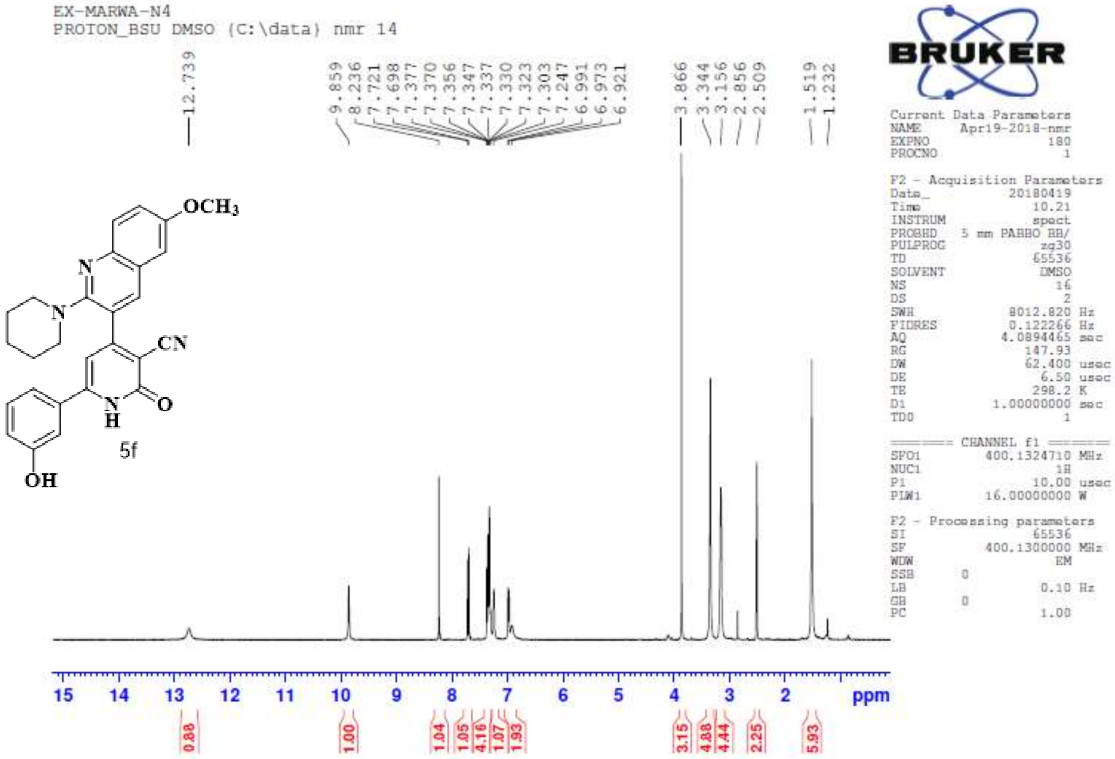

H[1]-NMR spectra of compound 5f

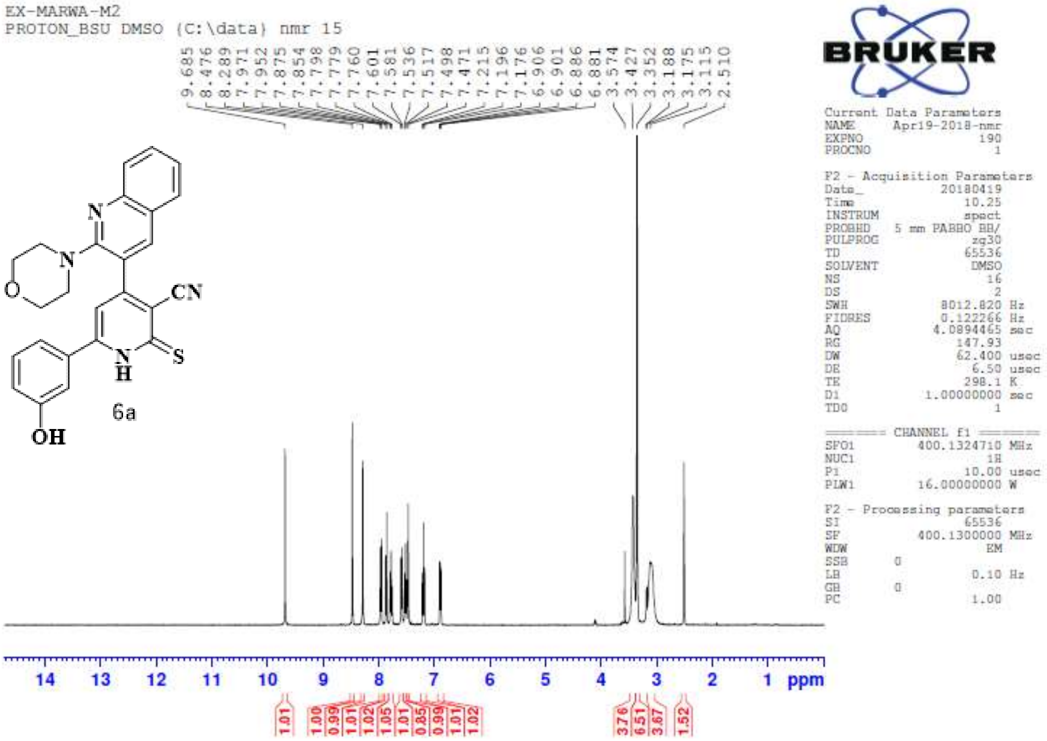

H[1]-NMR spectra of compound 6a

Information Classification: General

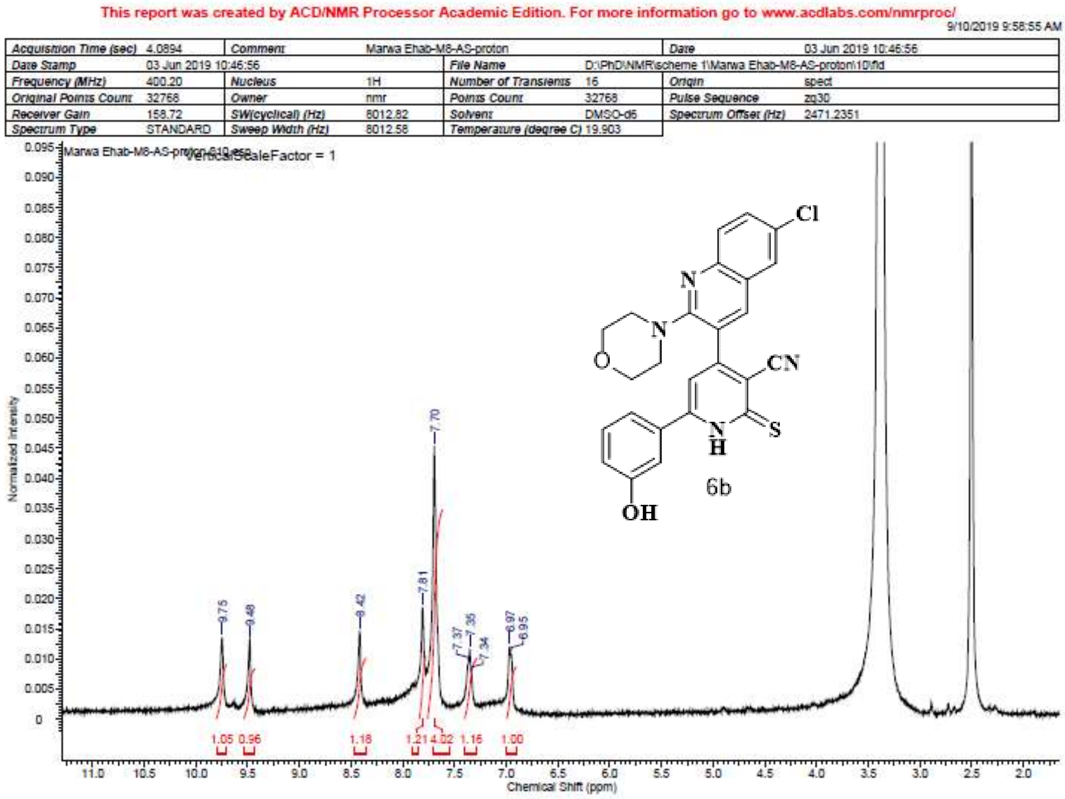

H^1-NMR spectra of compound 6b

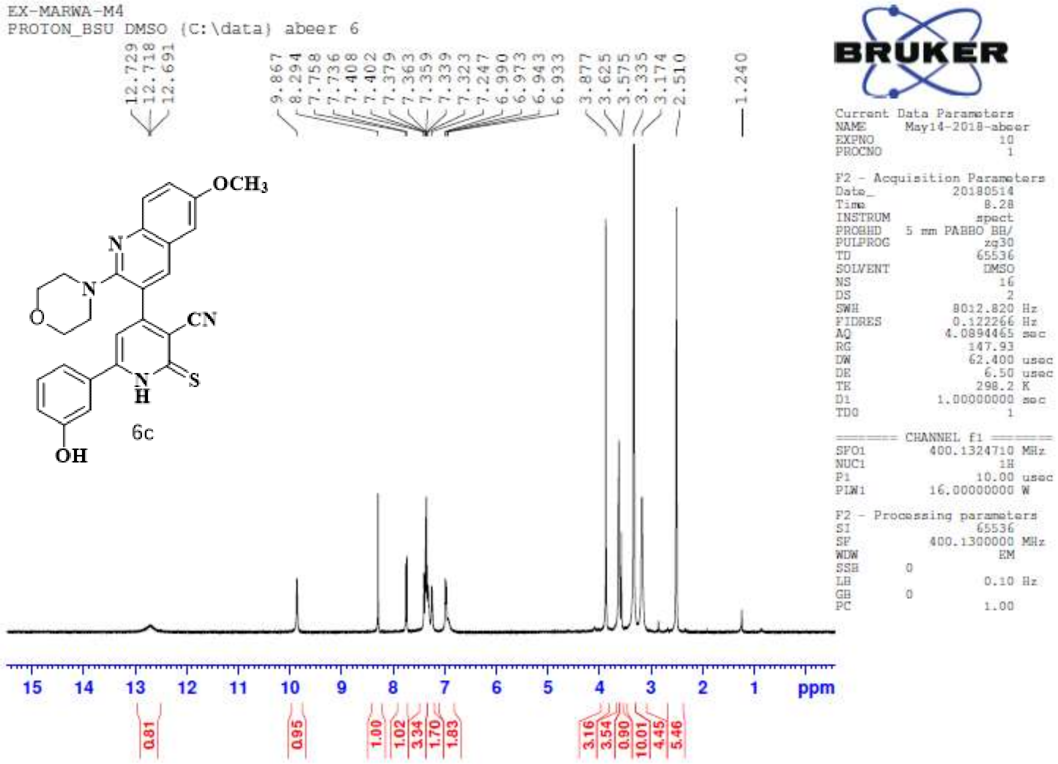

H^1-NMR spectra of compound 6c

Information Classification: General

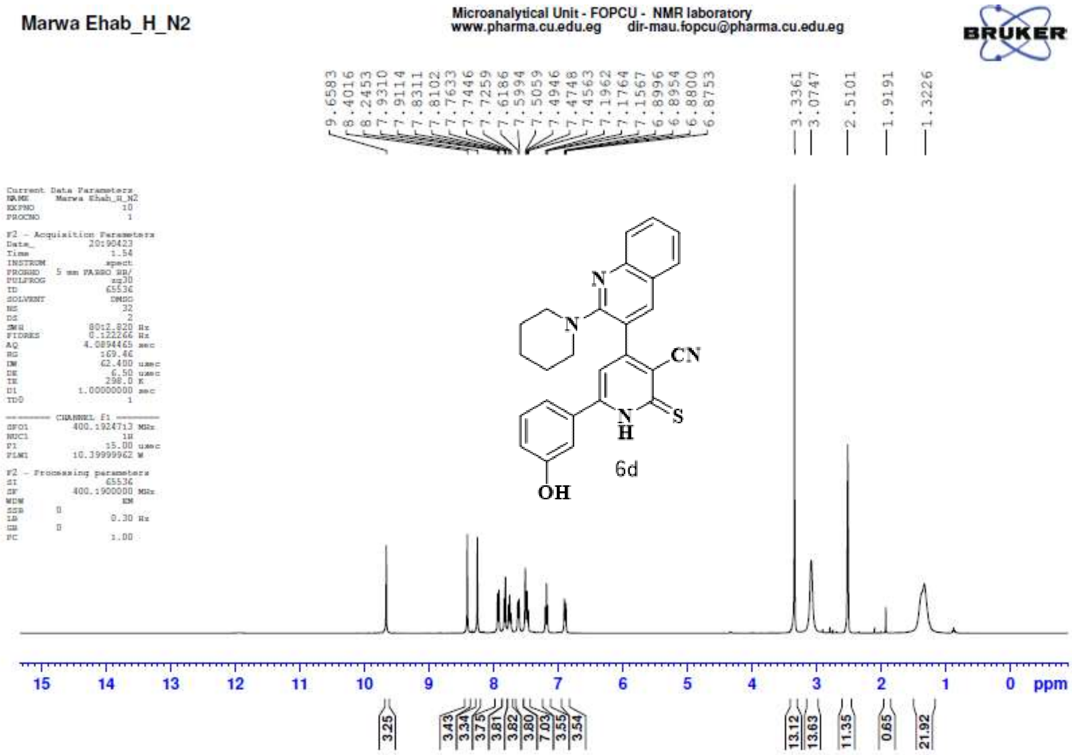

H[1]-NMR spectra of compound 6d

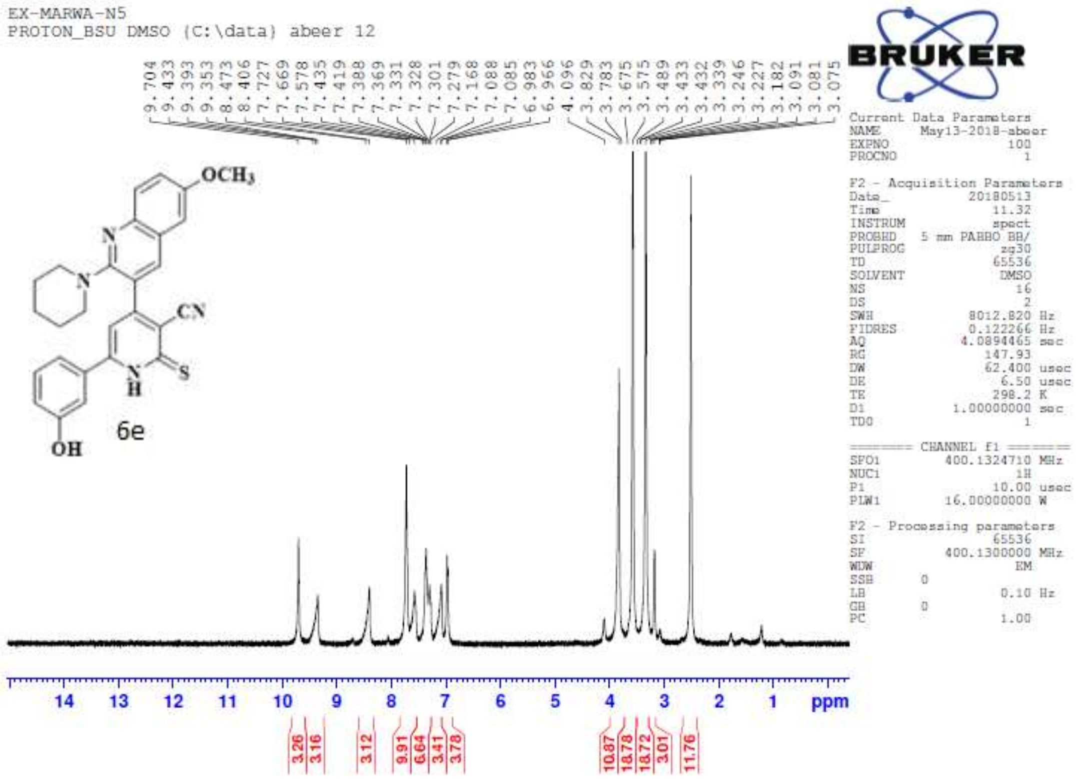

H[1]-NMR spectra of compound 6e

Information Classification: General

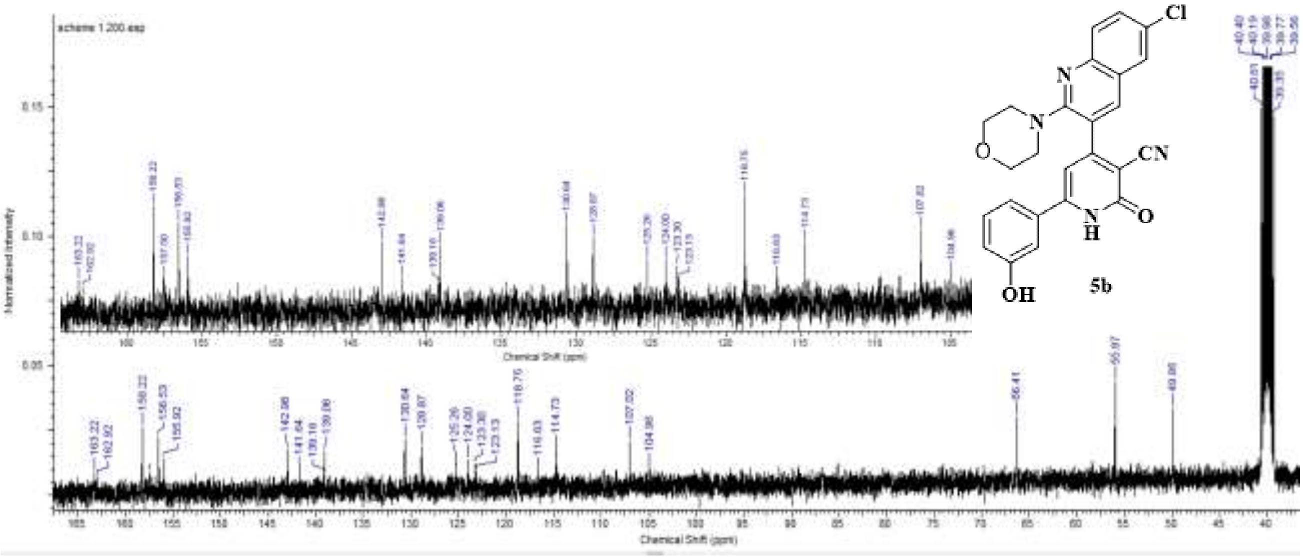

C^{13}- NMR spectra of compound 5b

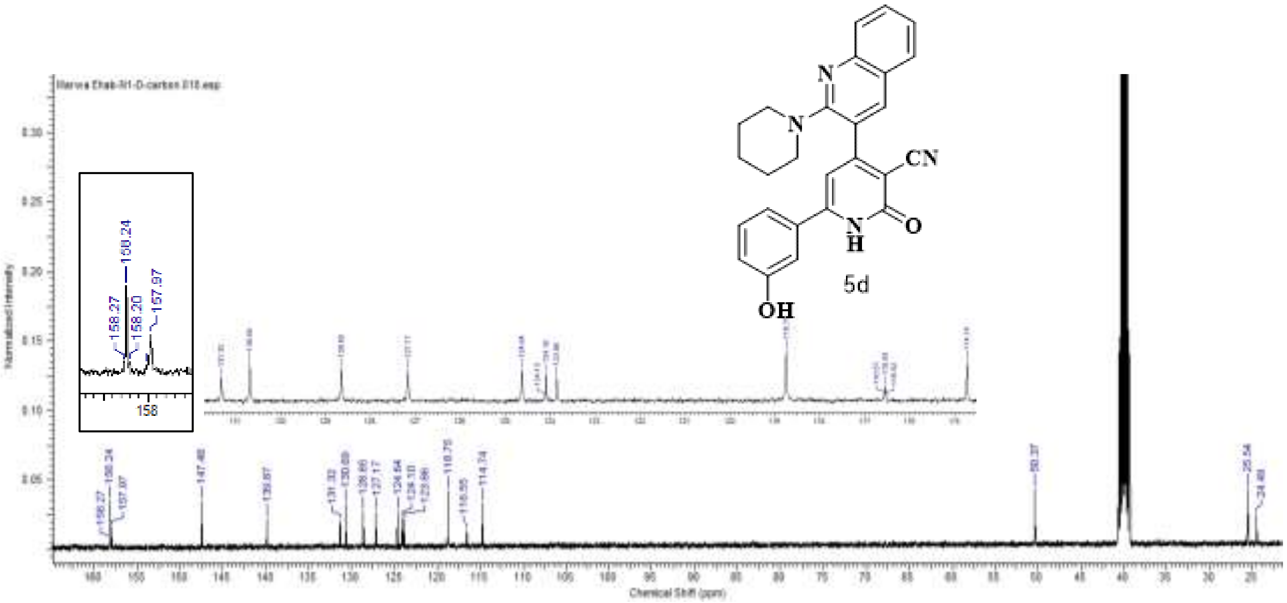

C^{13}- NMR spectra of compound 5d

Information Classification: General

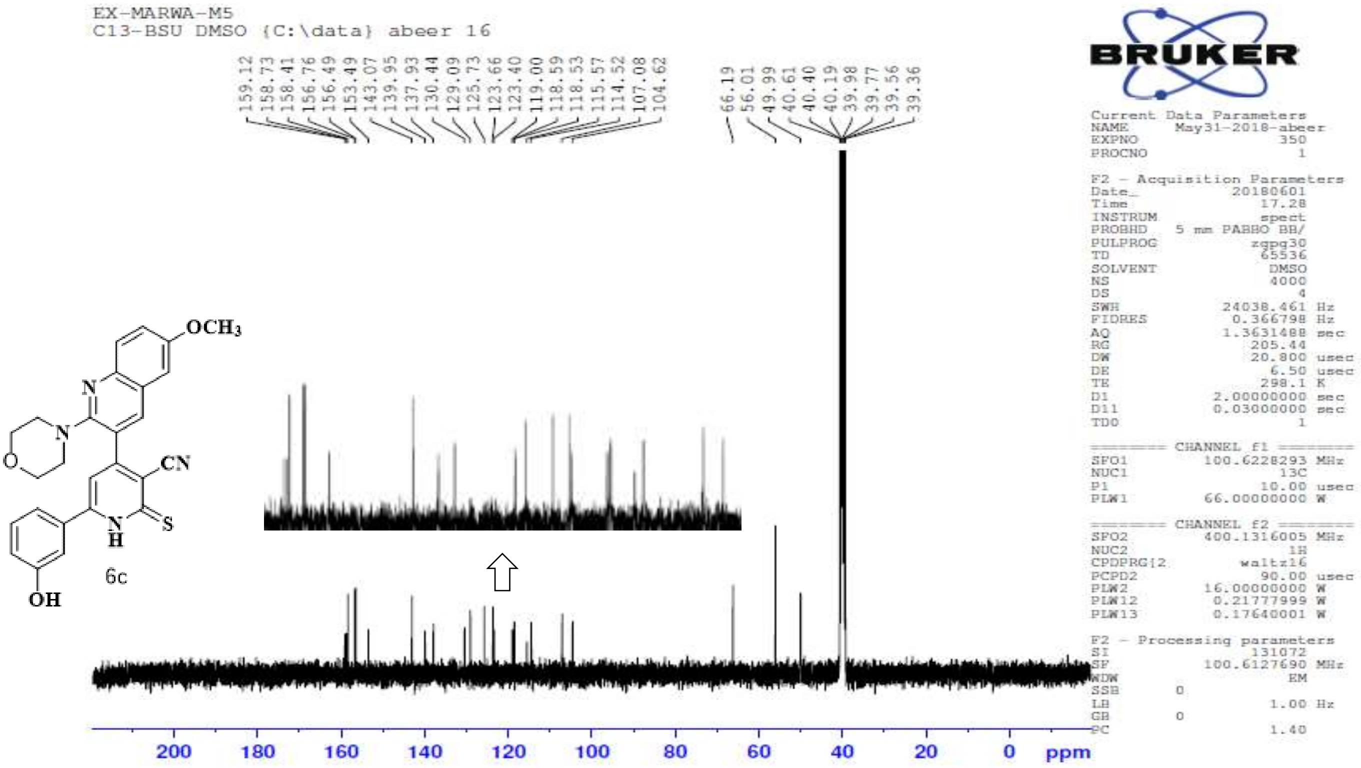

C[13]- NMR spectra of compound 6c

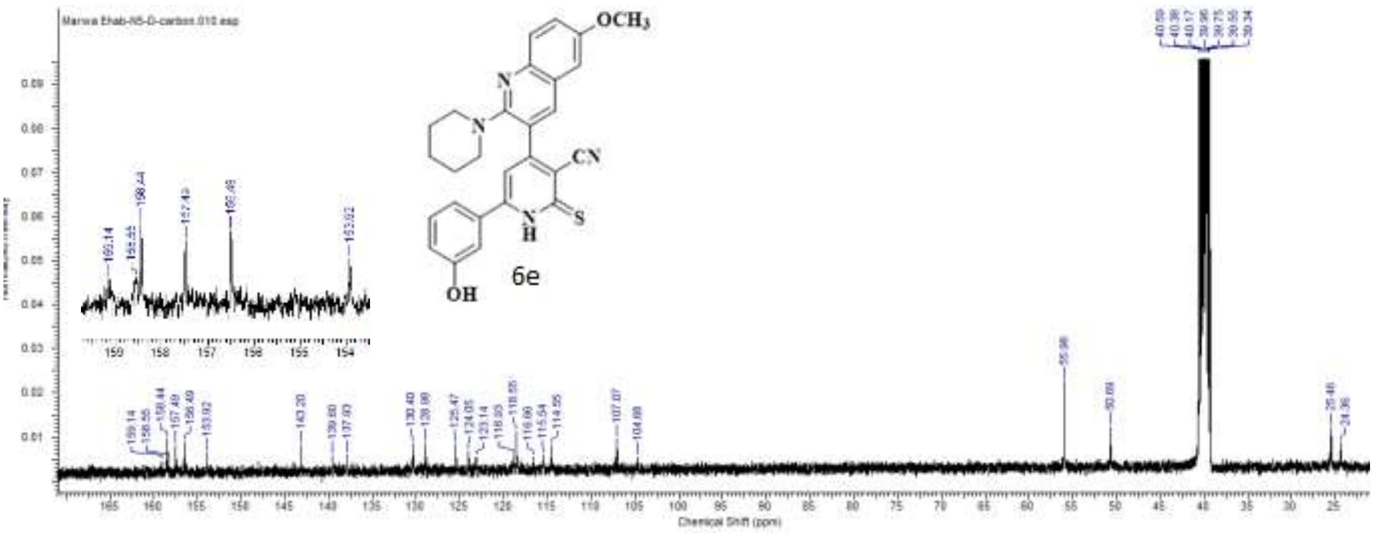

C[13]- NMR spectra of compound 6e

Information Classification: General

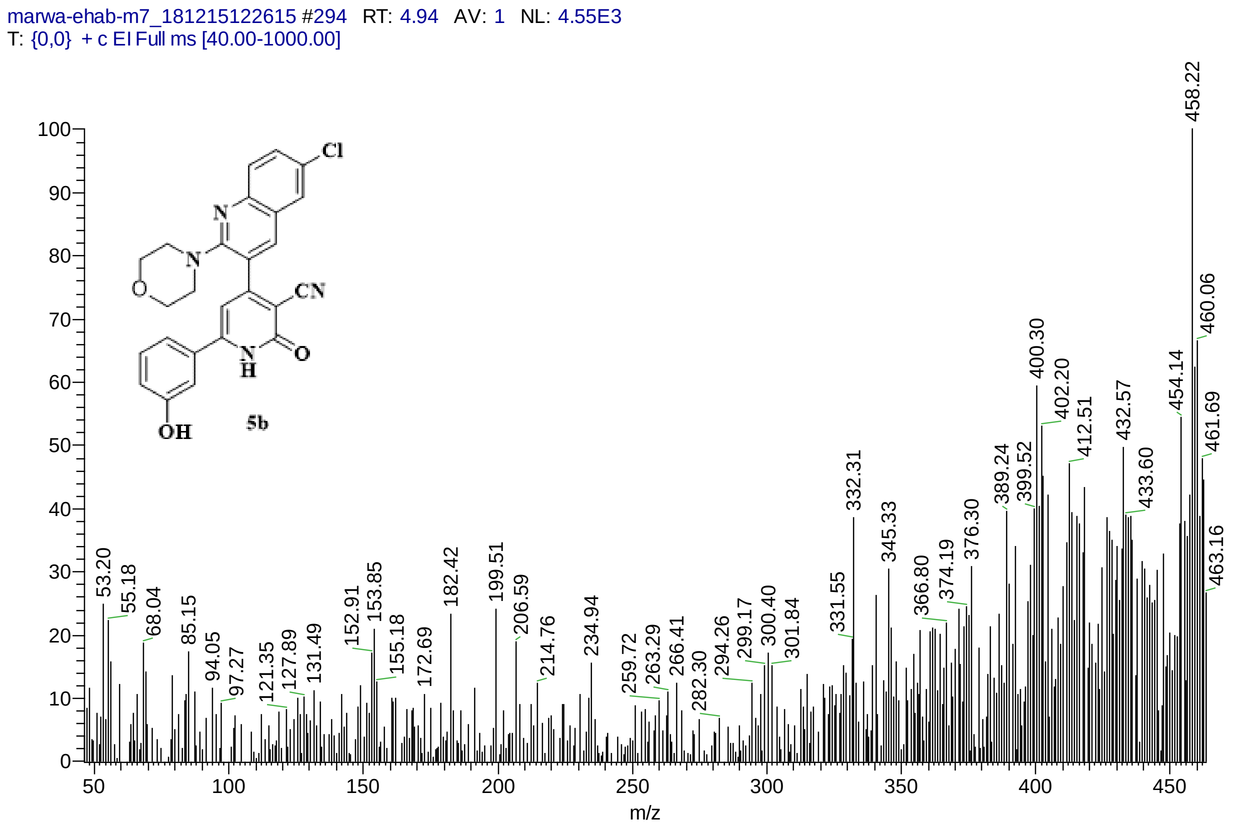

EI/MS spectrum of compound **5b**

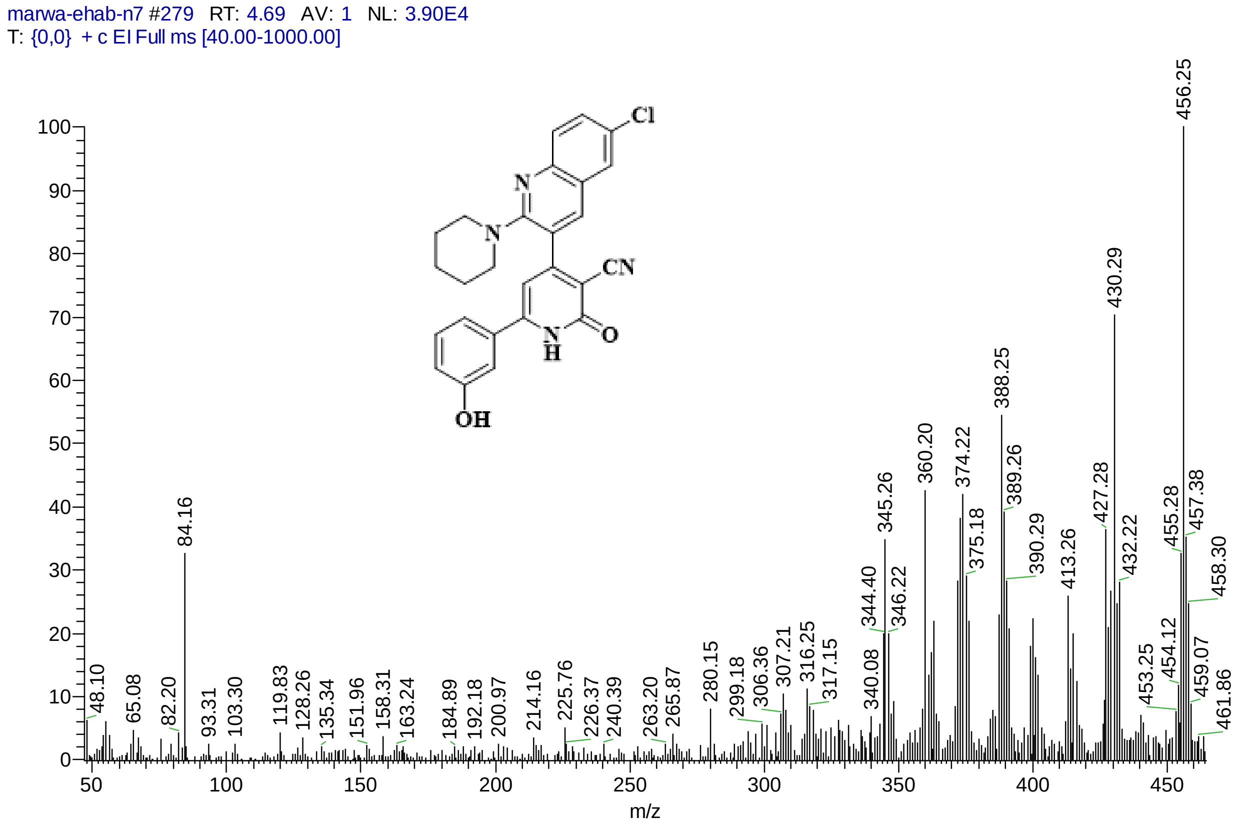

EI/MS spectrum of compound **5e**

Information Classification: General

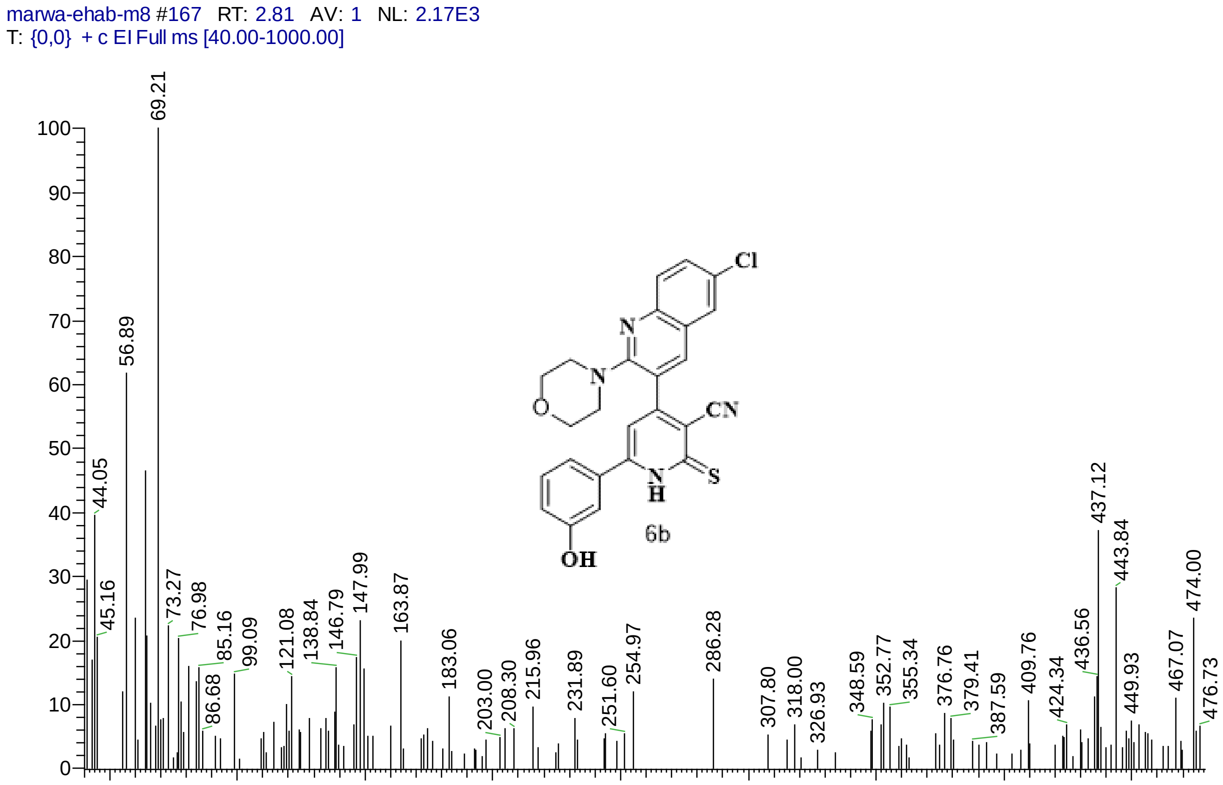

EI/MS spectrum of **compound 6b**

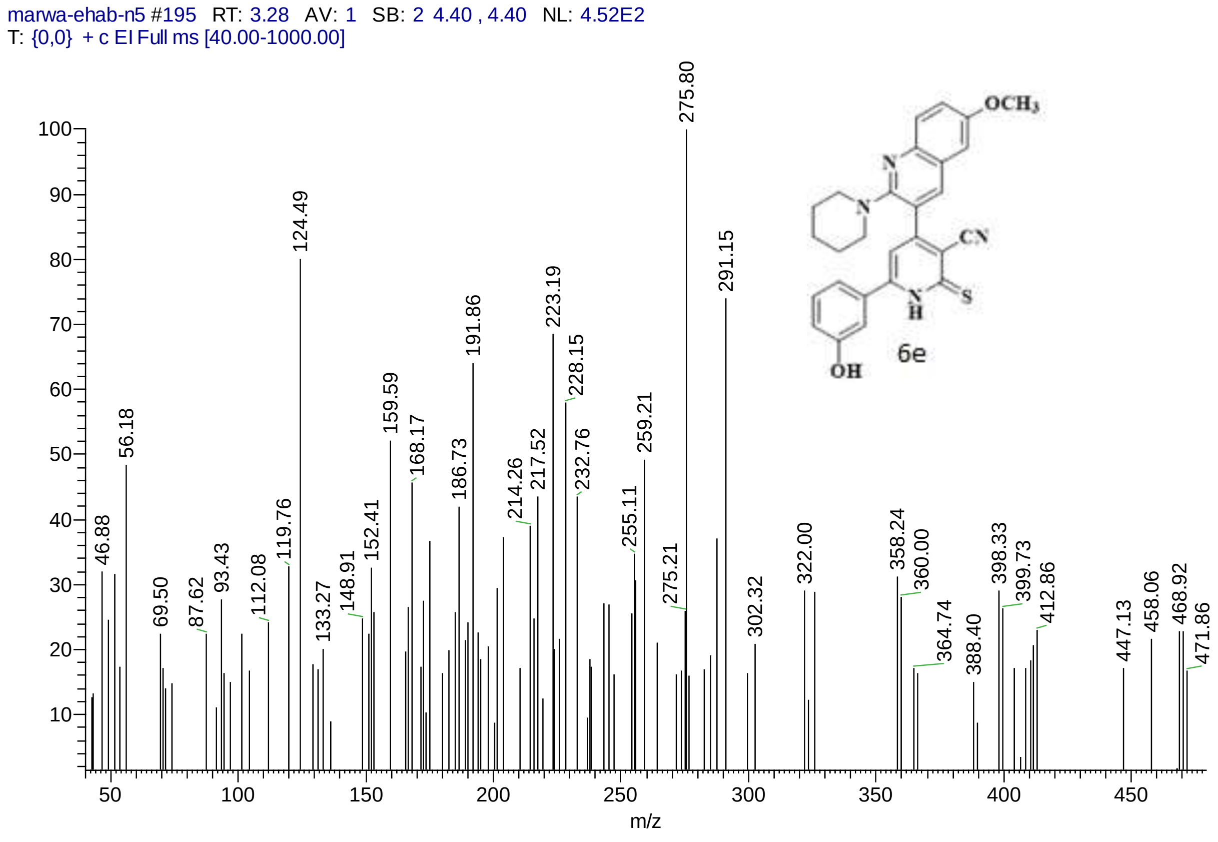

EI/MS spectrum of compound **6e**

Information Classification: General

Al-Azhar University
The Regional Center for Mycology and Biotechnology

Requester Data:

Name: Dr. Marwa Ehab Mohamed
Authority: Faculty of Pharmacy,
Alexandria University

Sample Data:

Eleven samples had been submitted for elemental analysis.

Analysis Report:

Sample Code	C%	H%	N%
CH_2	68.01	3.43	7.79
CH_5	68.56	3.88	7.42
M_1 5a	71.02	4.89	13.43
M_4 5c	68.94	5.12	12.59
M_7 5b	65.27	4.28	12.47
N_1 5d	74.19	5.39	13.53
N_4 5f	71.90	5.26	12.56
N_7 5e	68.60	4.80	12.49
O_2	70.69	3.84	11.97
O_5	68.80	3.85	10.79
O_8	64.89	3.32	10.85

INVESTIGATOR **DIRECTOR**

Information Classification: General

Requester Data:

Name: Dr. Marwa Ehab Mohamed
Authority: Faculty of Pharmacy,
Alexandria University

Sample Data:

Twenty samples had been submitted for elemental analysis.

Analysis Report:

Sample Code	C%	H%	N%
M_1 eN	68.71	5.24	19.38
M_1 J	71.09	5.21	12.96
M_2 6a	68.42	4.75	12.94
M_4 e	70.38	5.84	11.50
M_4 k	69.87	5.68	11.79
M_5 6c	66.59	4.83	11.75
M_7 a	64.58	4.59	11.34
M_7 b	62.96	4.13	11.06
M_7 e	67.40	5.28	11.37
M_7 e N	63.26	4.69	17.58
M_7 f	63.44	4.21	11.39
M_7 h	66.81	4.62	11.94
M_7 J	66.26	4.69	11.72
M_7 k	66.49	4.88	11.69
M_8 6b	63.45	4.11	11.94
N_2 6d	71.47	5.28	13.02
N_5 6f	[illegible]43	5.34	12.13
O_1	6e [illegible]1.49	4.06	15.95
O_4	68.95	4.32	14.67
O_7	64.71	3.19	14.62

INVESTIGATOR **DIRECTOR**

Information Classification: General

Scheme 2:

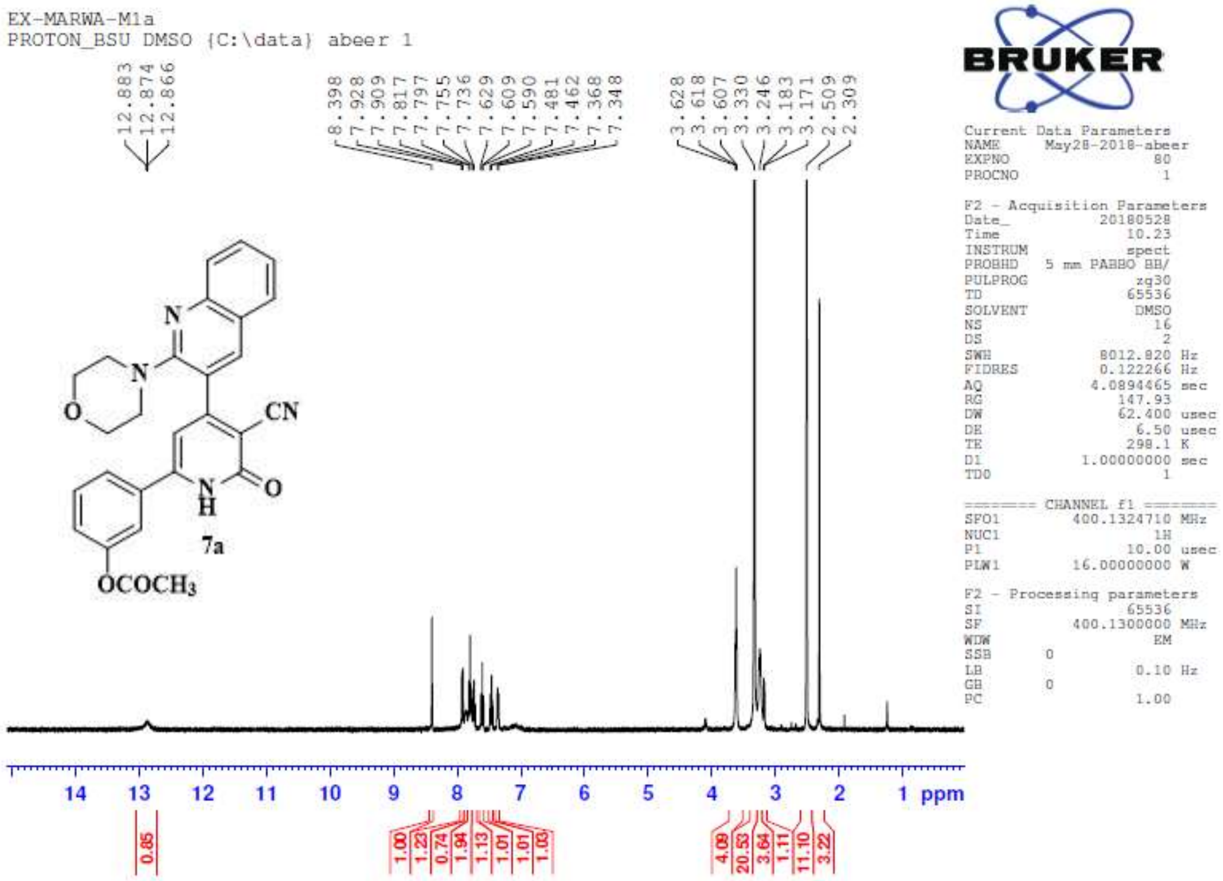

H1-NMR spectra of compound 7a

Information Classification: General

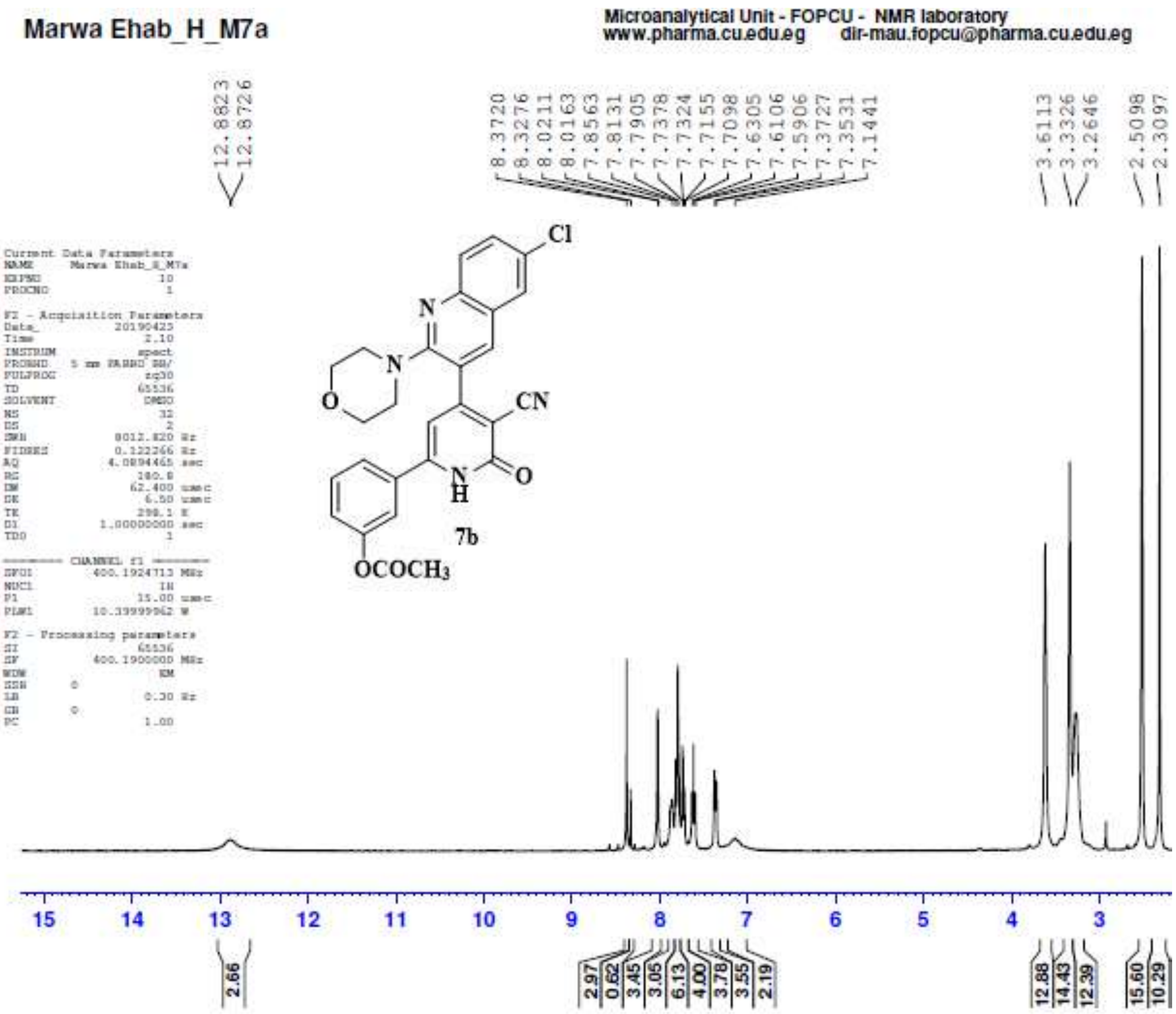

H^1-NMR spectra of compound 7b

Information Classification: General

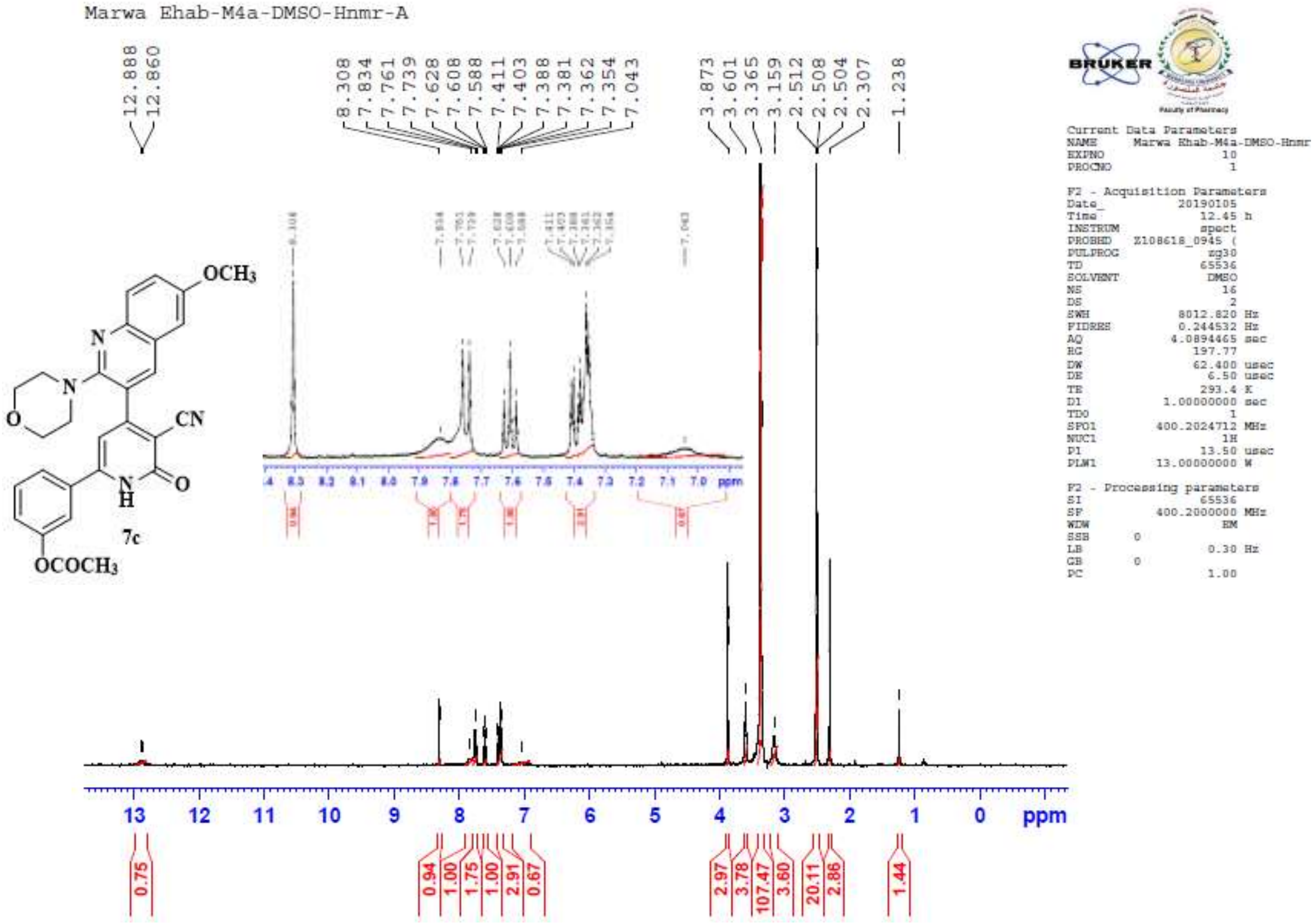

H[1]-NMR spectra of compound 7c

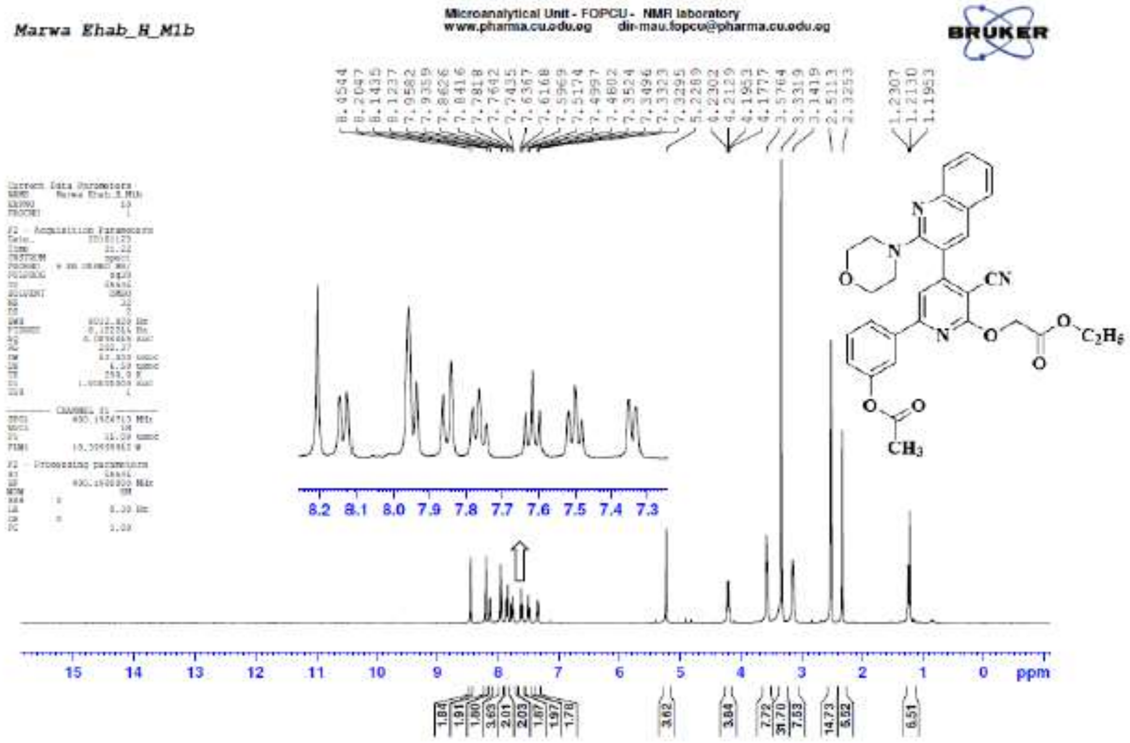

H[1]-NMR spectra of compound 8a

Information Classification: General

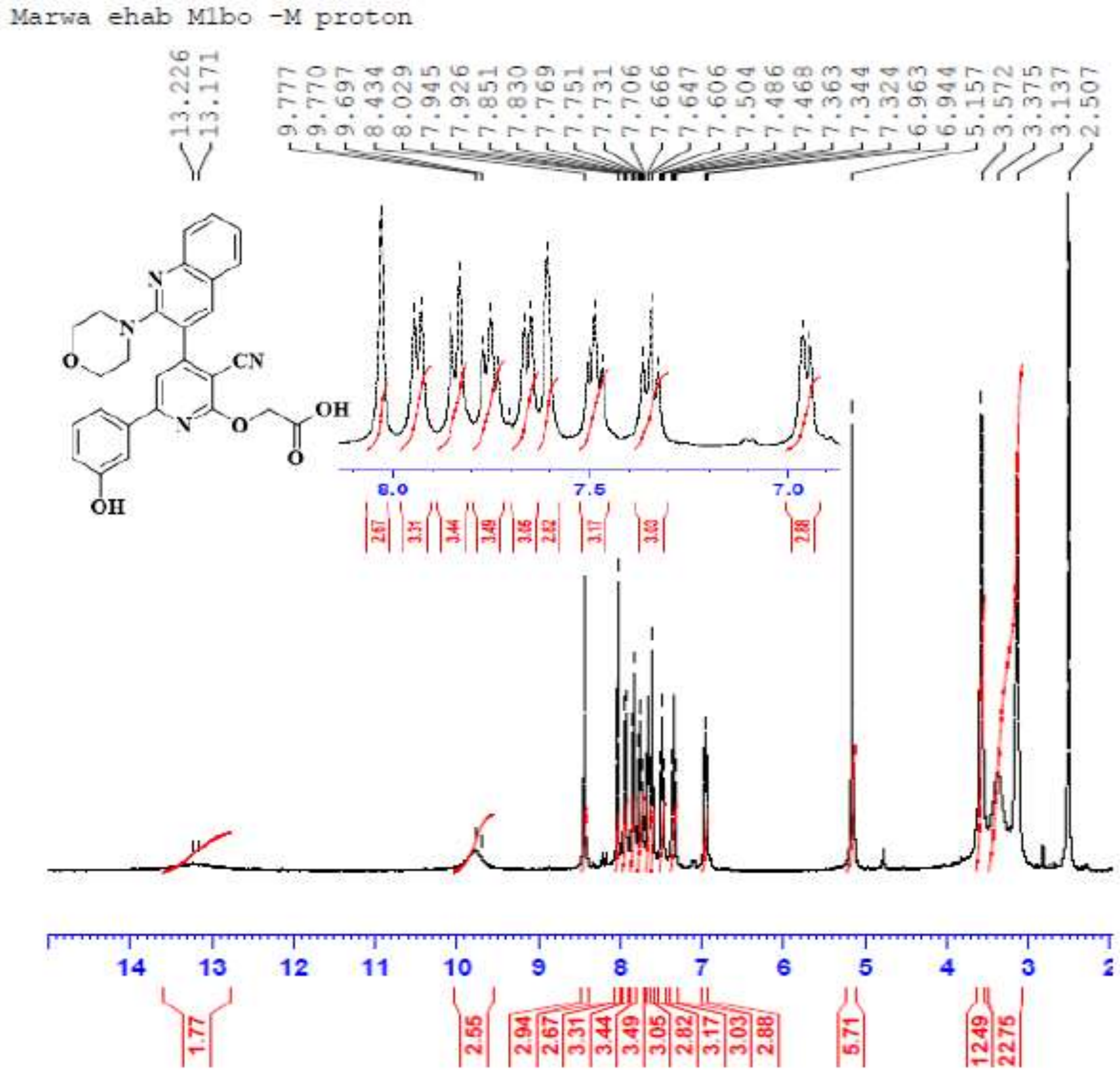

H^1-NMR spectra of compound 9a

Information Classification: General

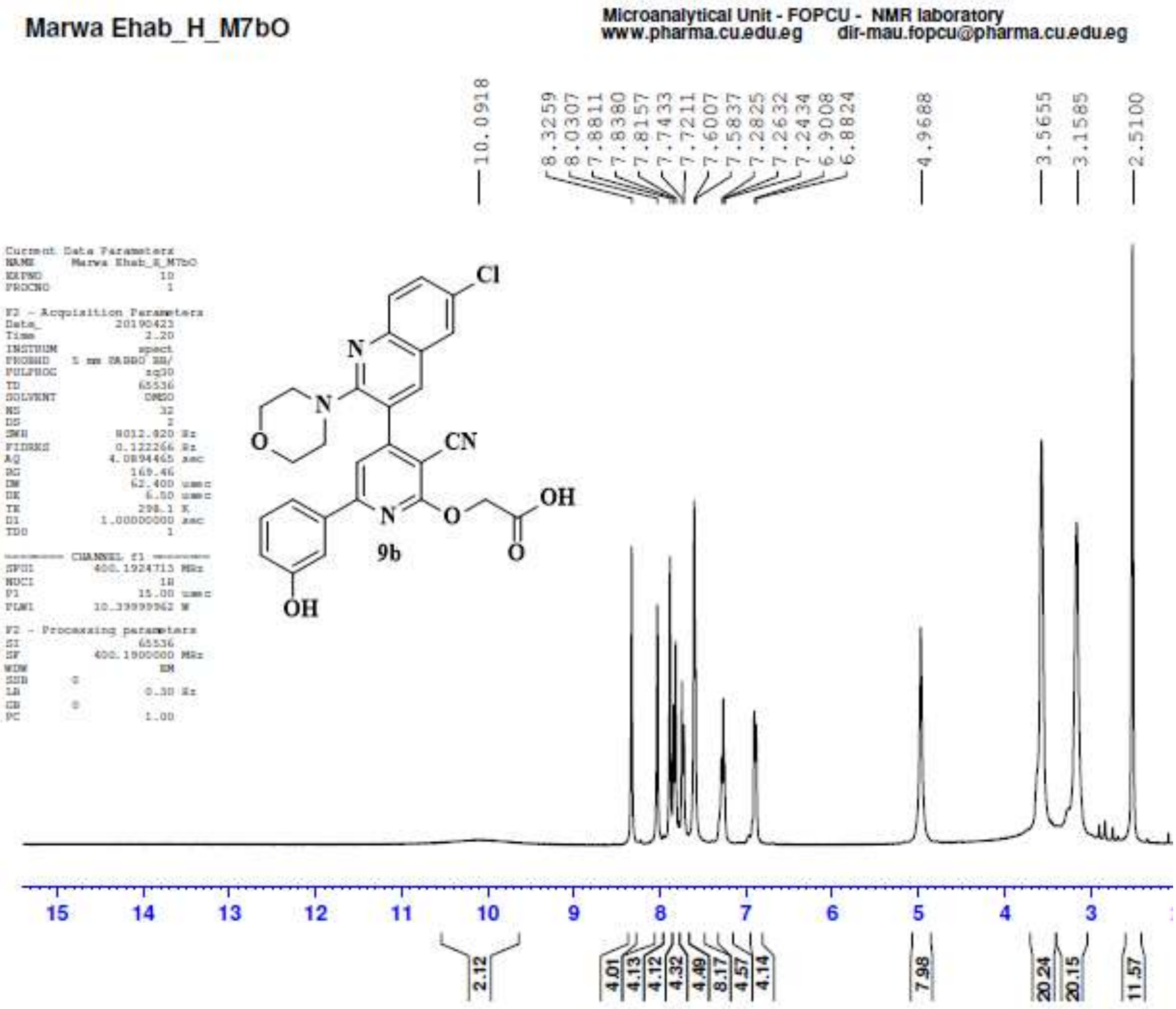

H¹-NMR spectra of compound 9b

Information Classification: General

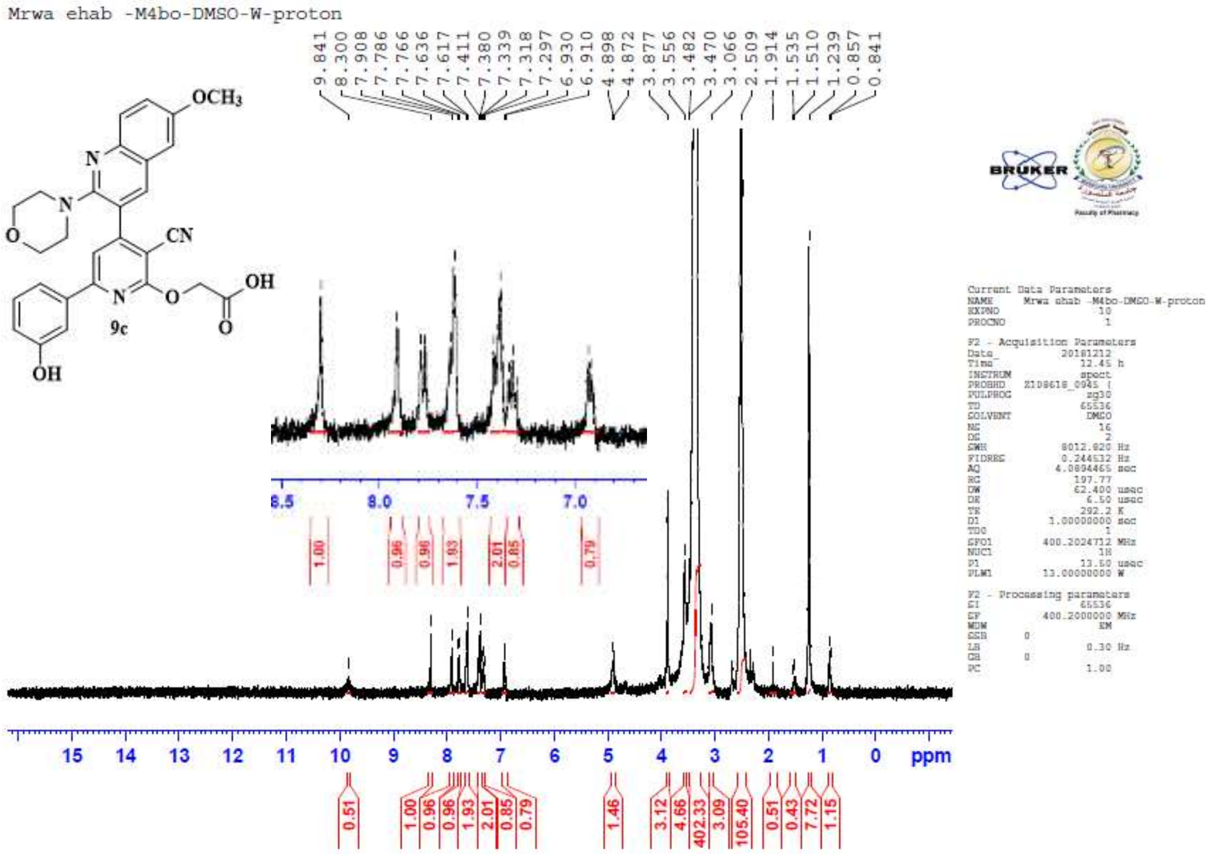

H[1]-NMR spectra of compound 9c

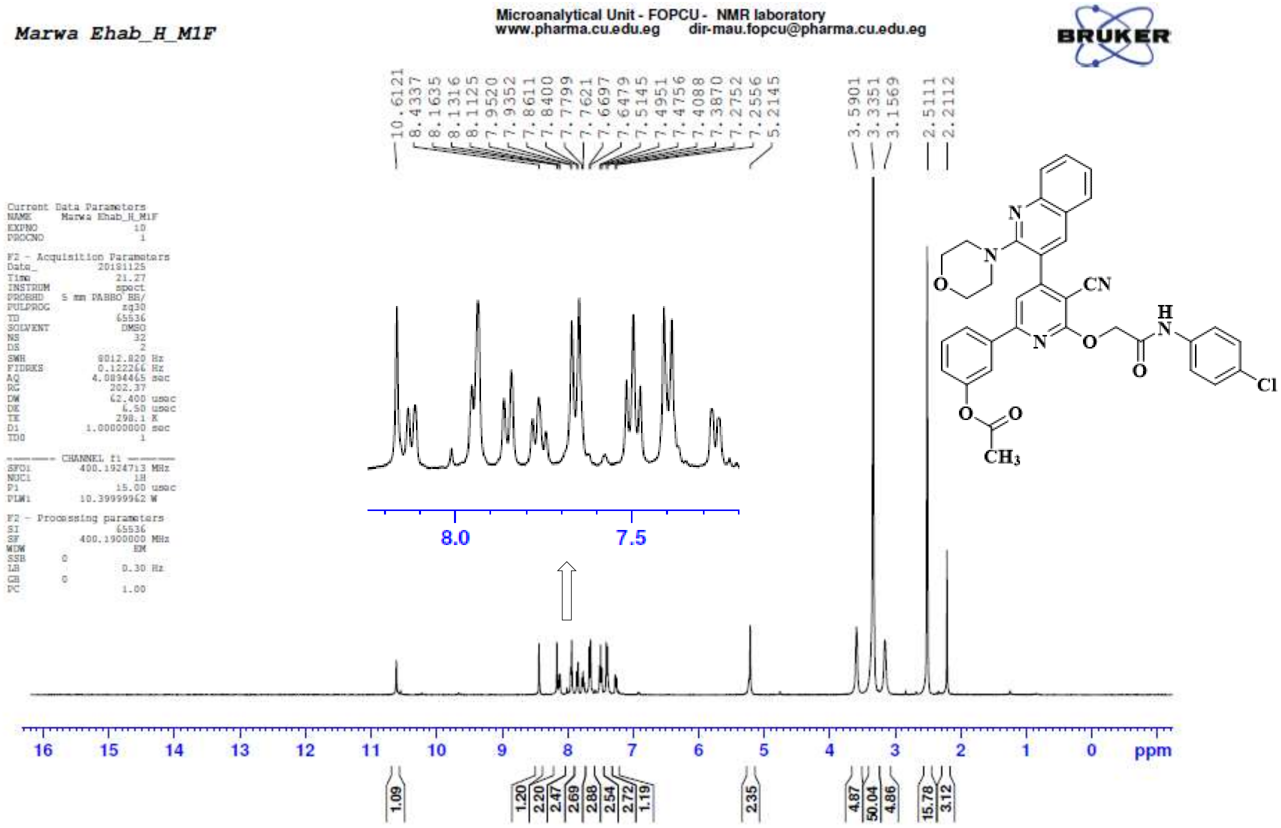

H[1]-NMR spectra of compound 10b

Information Classification: General

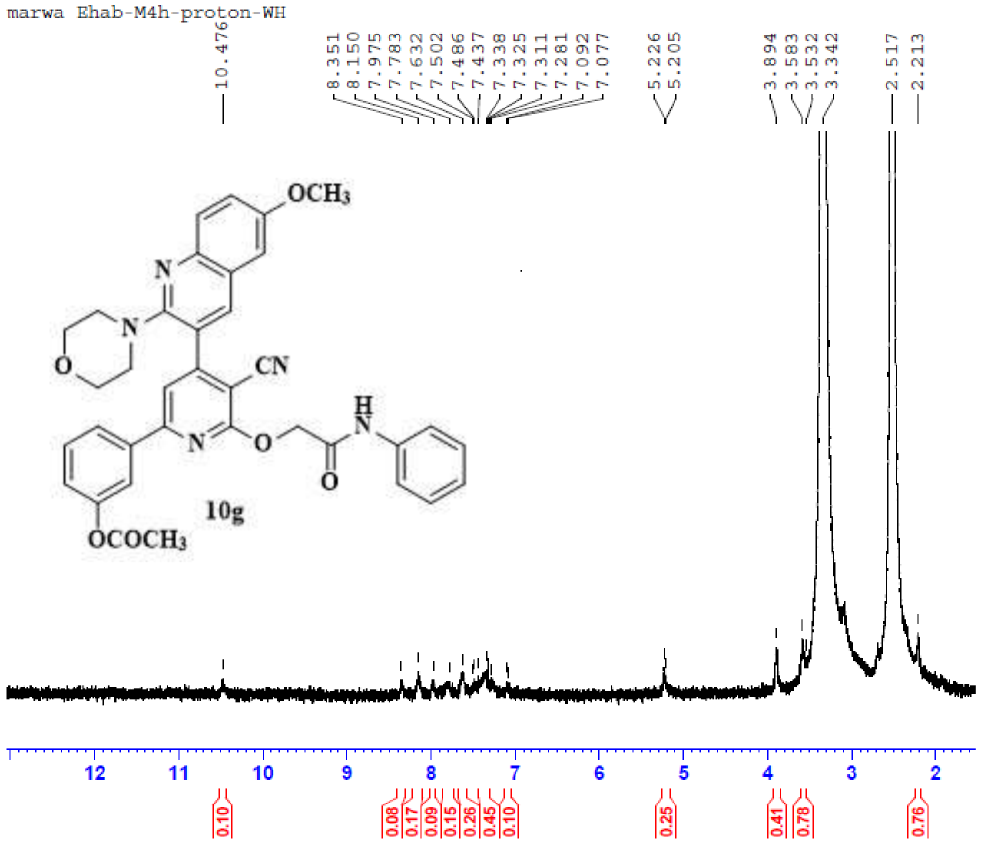

H[1]-NMR spectra of compound 10g

Information Classification: General

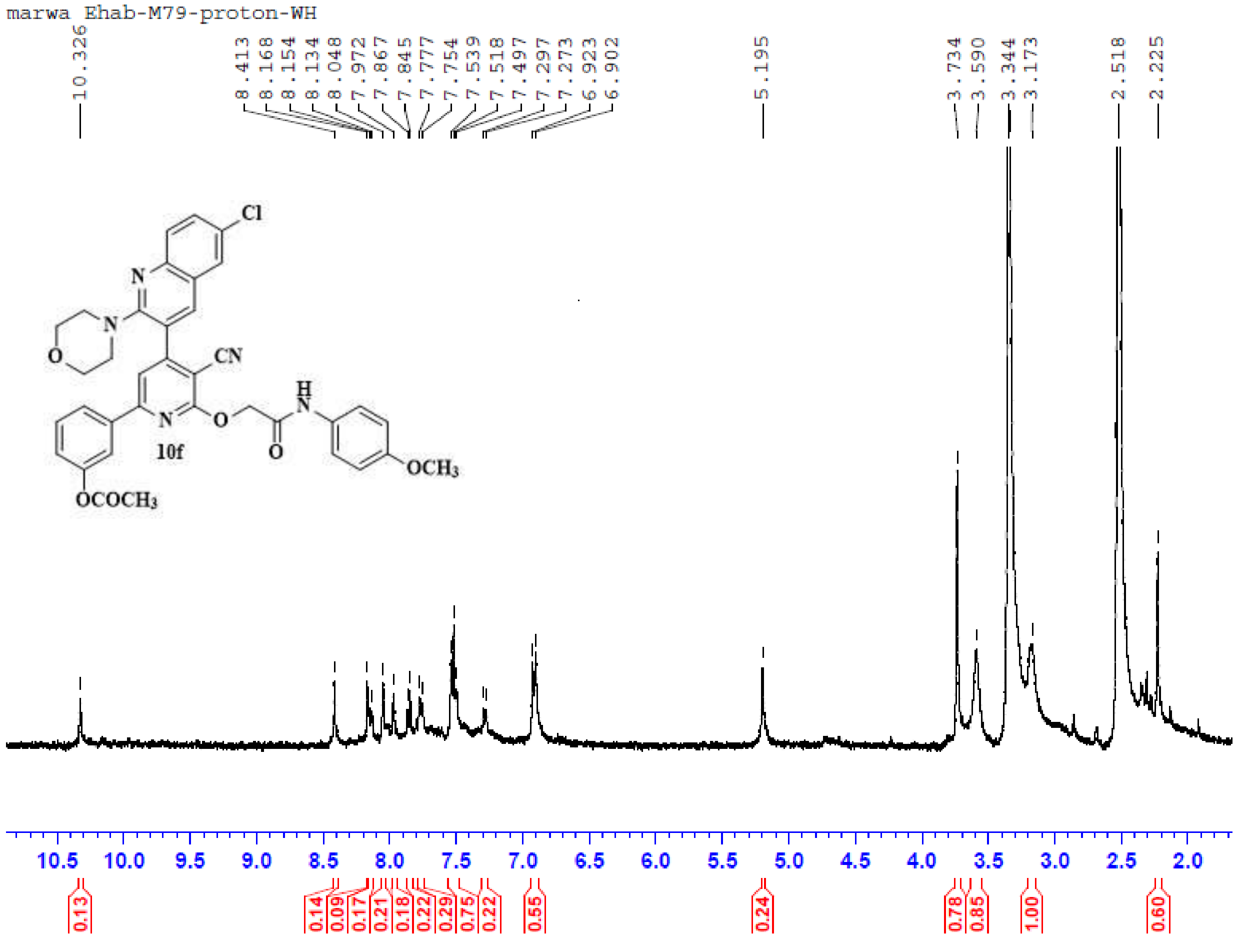

H[1]-NMR spectra of compound 10f

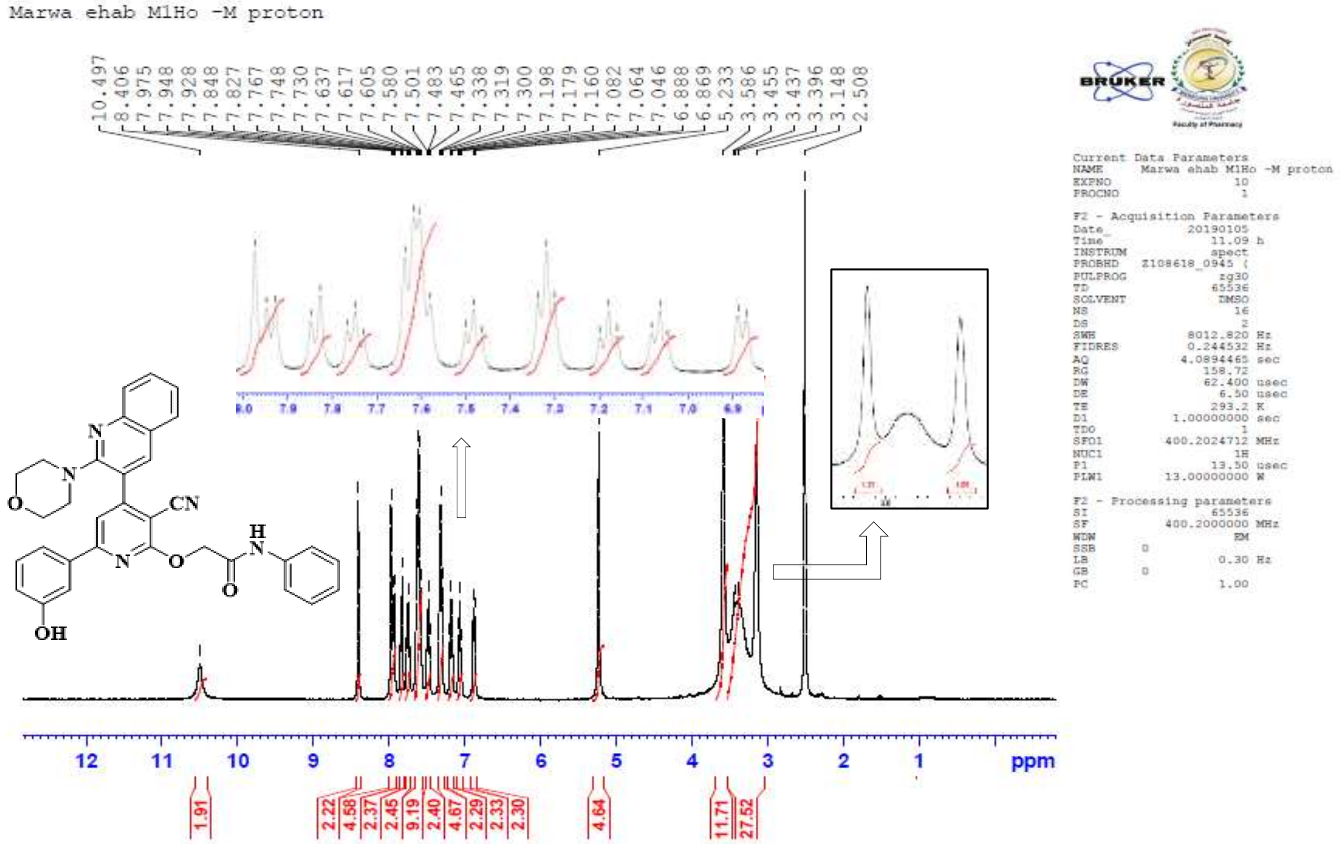

H[1]-NMR spectra of compound 11a

Information Classification: General

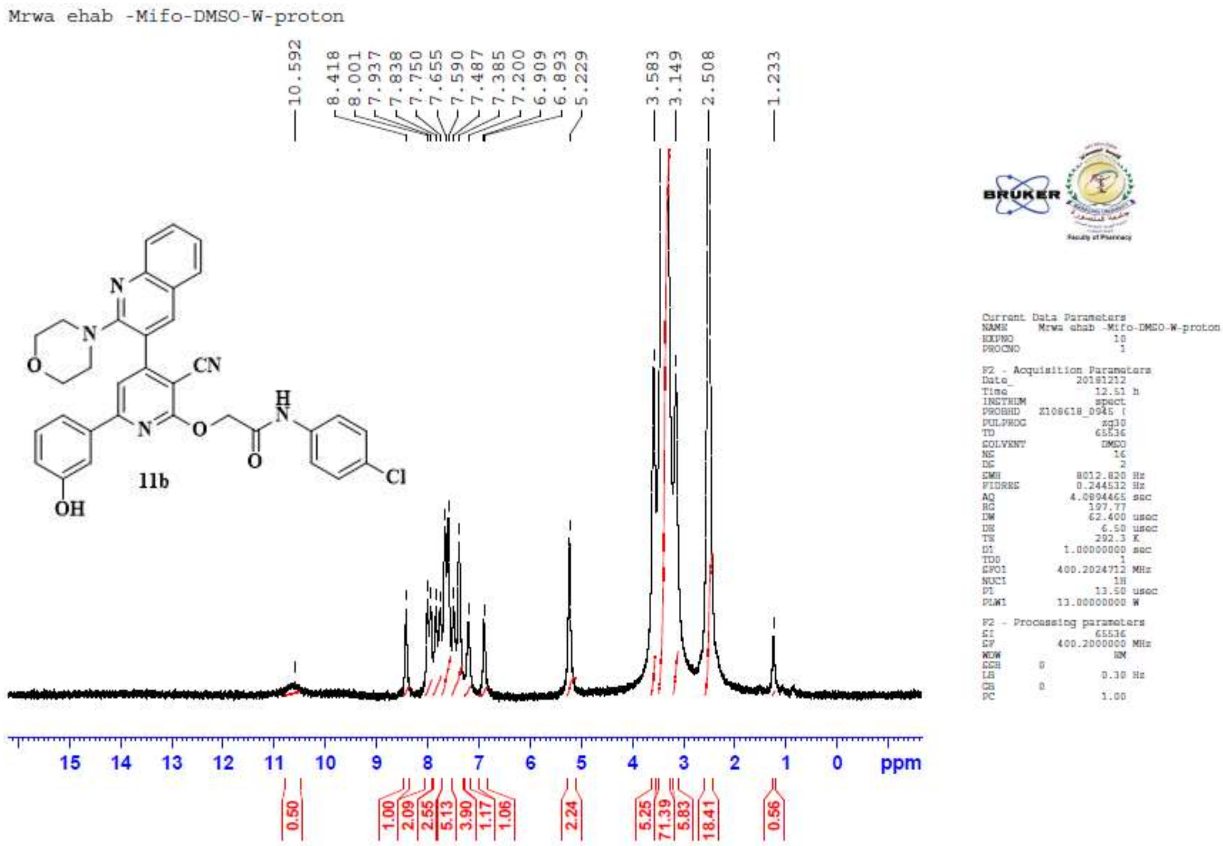

H[1]-NMR spectra of compound 11b

Information Classification: General

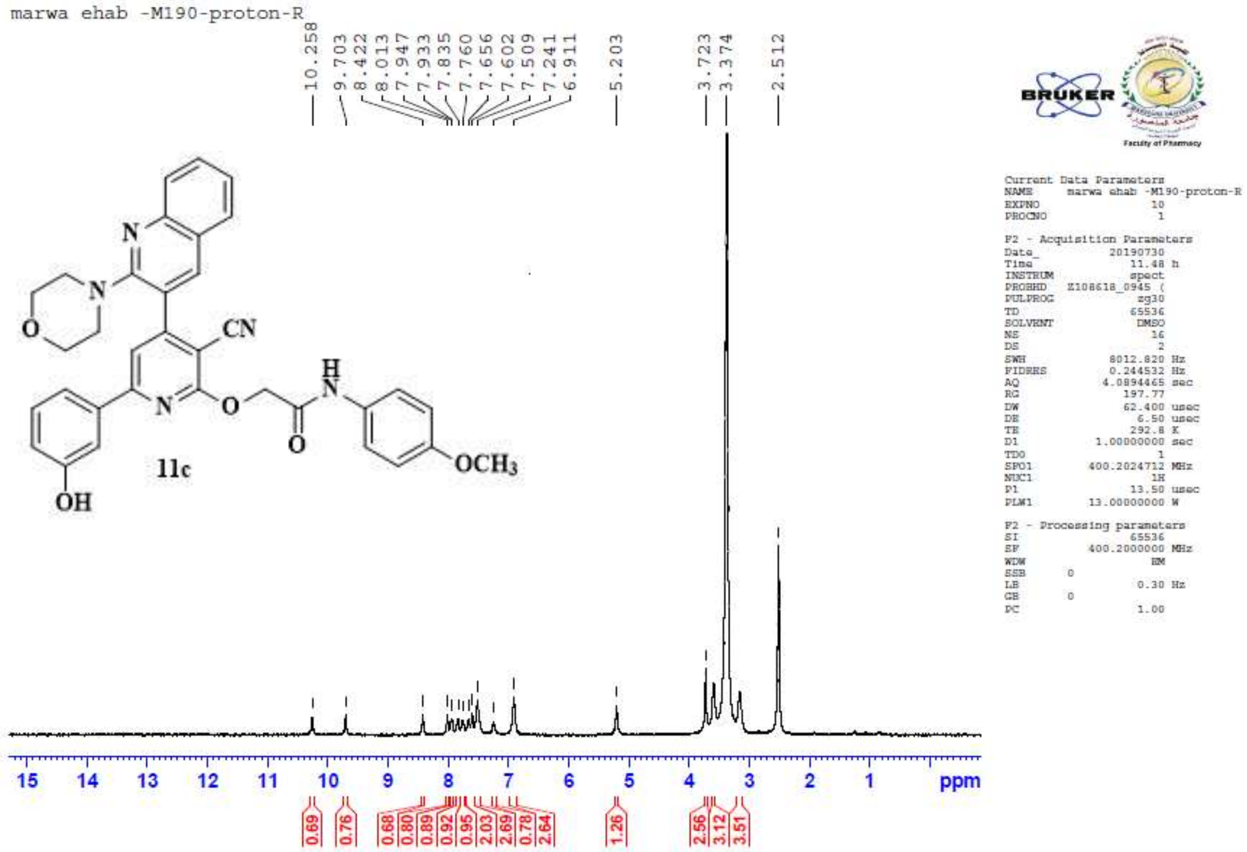

H[1]-NMR spectra of compound 11c

Information Classification: General

This report was created by ACD/NMR Processor Academic Edition. For more information go to www.acdlabs.com/nmrproc/

8/1/2019 1:48:18 PM

Acquisition Time (sec)	4.0894	Comment	Marwa Ehab-M7ho-AS-proton			Date	03 Jun 2019 10:51:12
Date Stamp	03 Jun 2019 10:51:12			File Name	D:\PhD\NMR\scheme 3\Marwa Ehab-M7ho-AS-proton\10\fid		
Frequency (MHz)	400.20	Nucleus	1H	Number of Transients	16	Origin	spect
Original Points Count	32768	Owner	nmr	Points Count	32768	Pulse Sequence	zg30
Receiver Gain	112.56	SW(cyclical) (Hz)	8012.82	Solvent	DMSO-d6	Spectrum Offset (Hz)	2471.2351
Spectrum Type	STANDARD	Sweep Width (Hz)	8012.58	Temperature (degree C)	19.913		

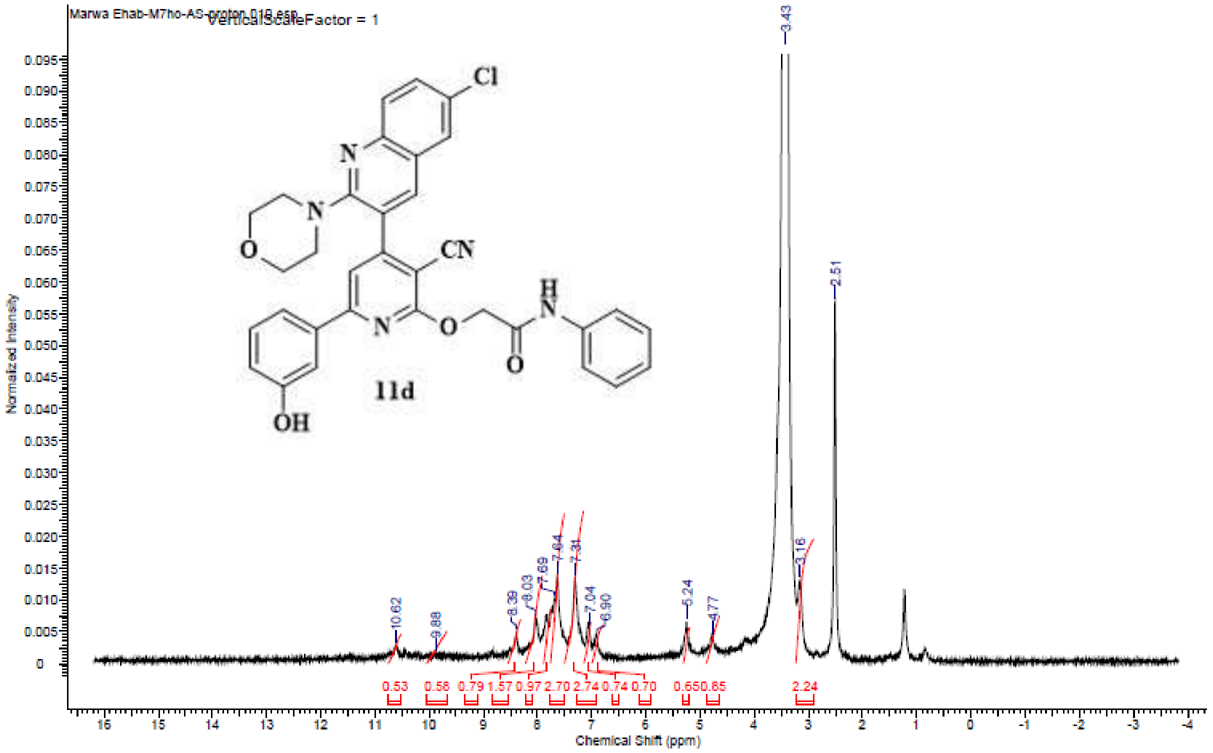

H[1]-NMR spectra of compound 11d

Information Classification: General

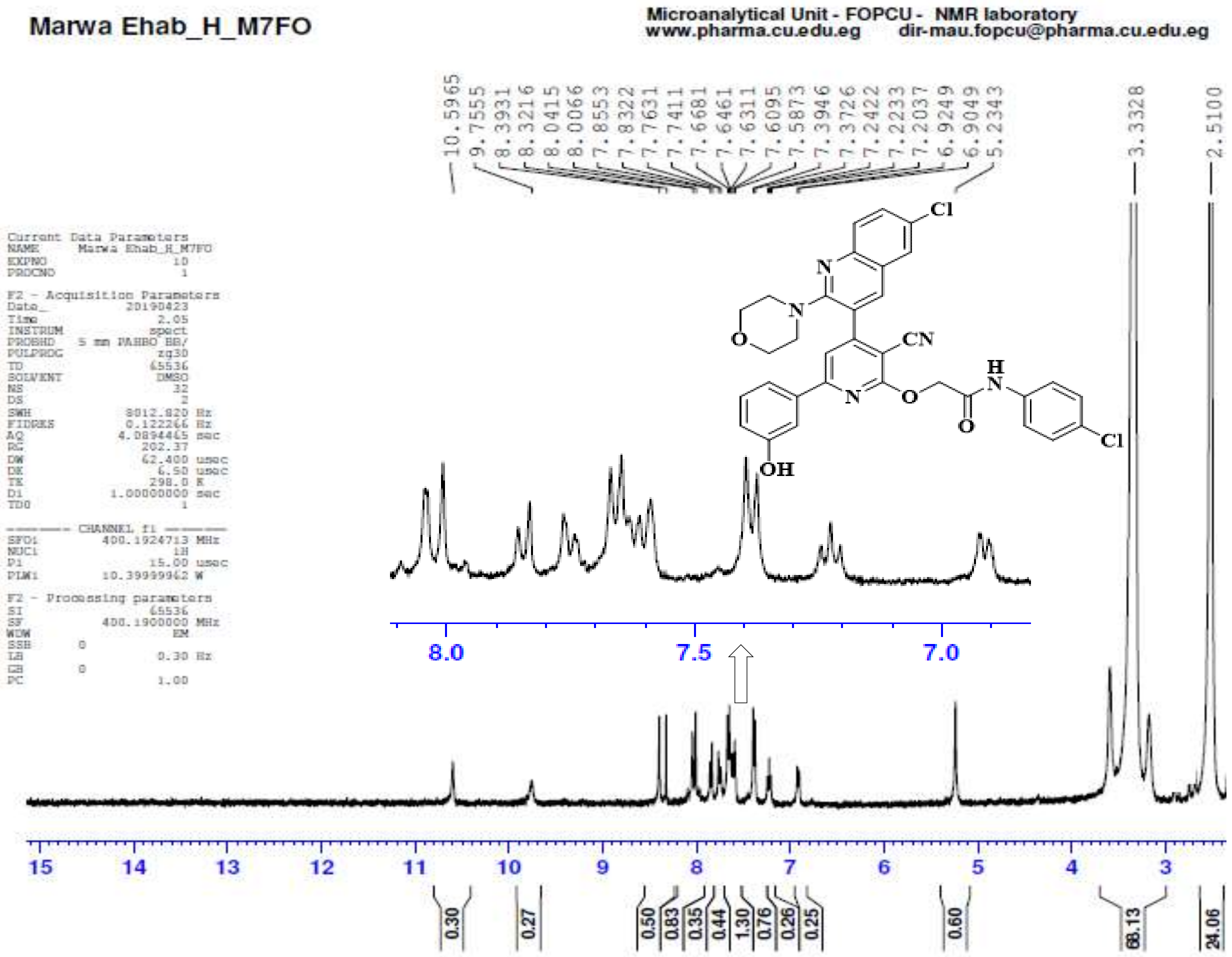

H[1]-NMR spectra of compound 11e

Information Classification: General

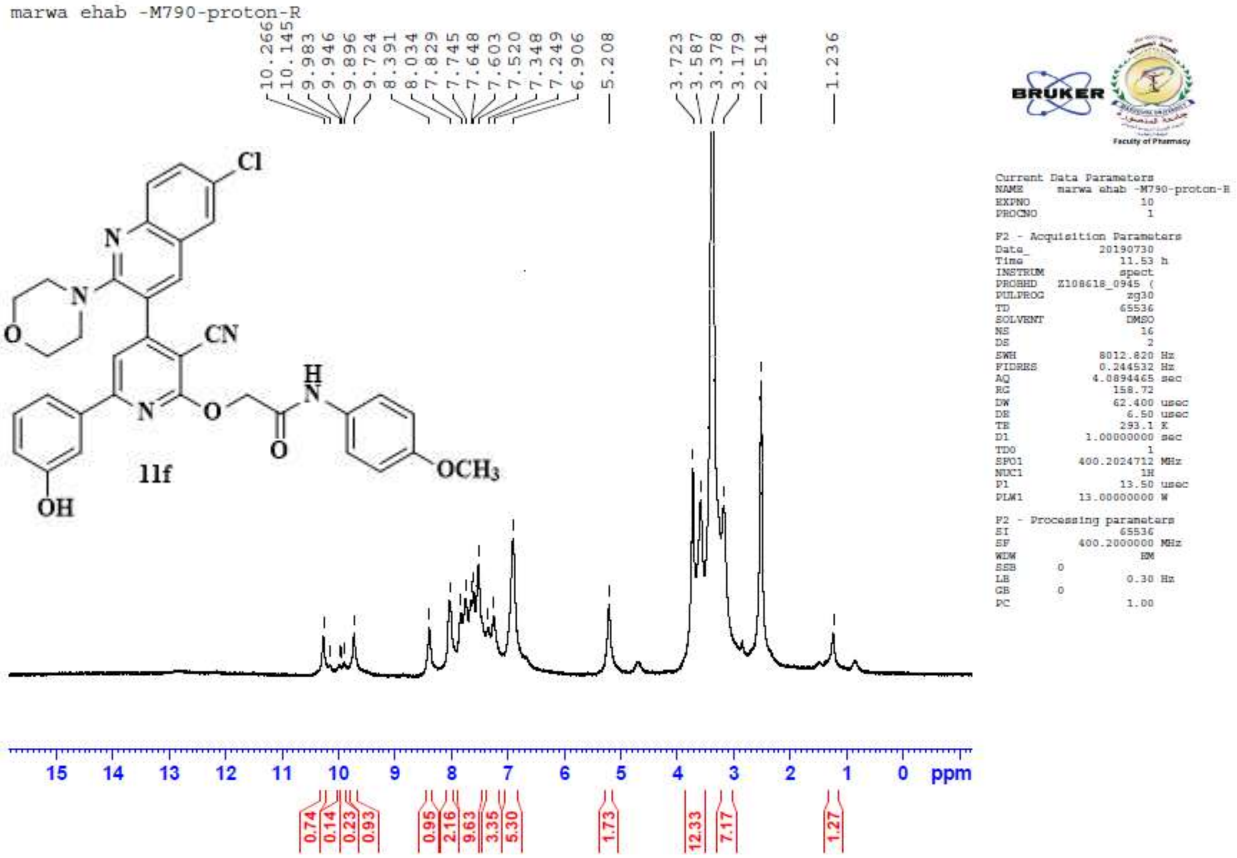

H[1]-NMR spectra of compound 11f

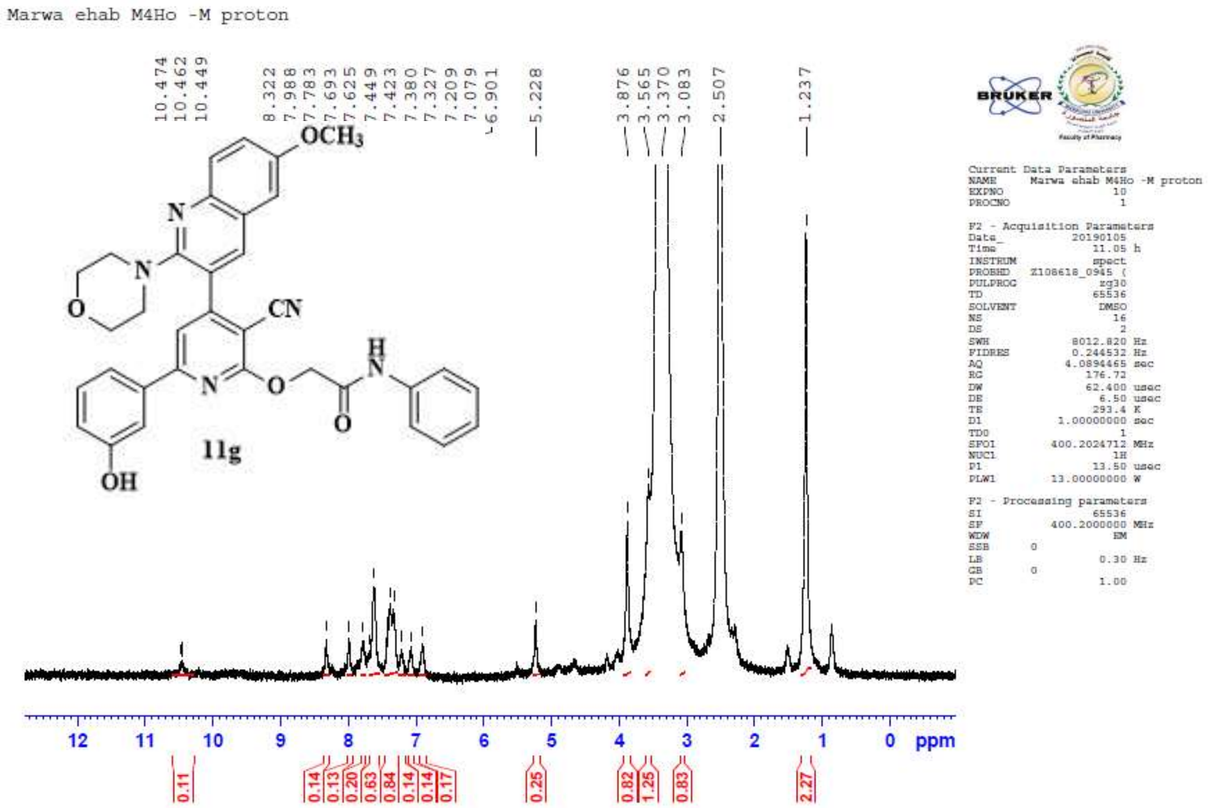

H[1]-NMR spectra of compound 11g

Information Classification: General

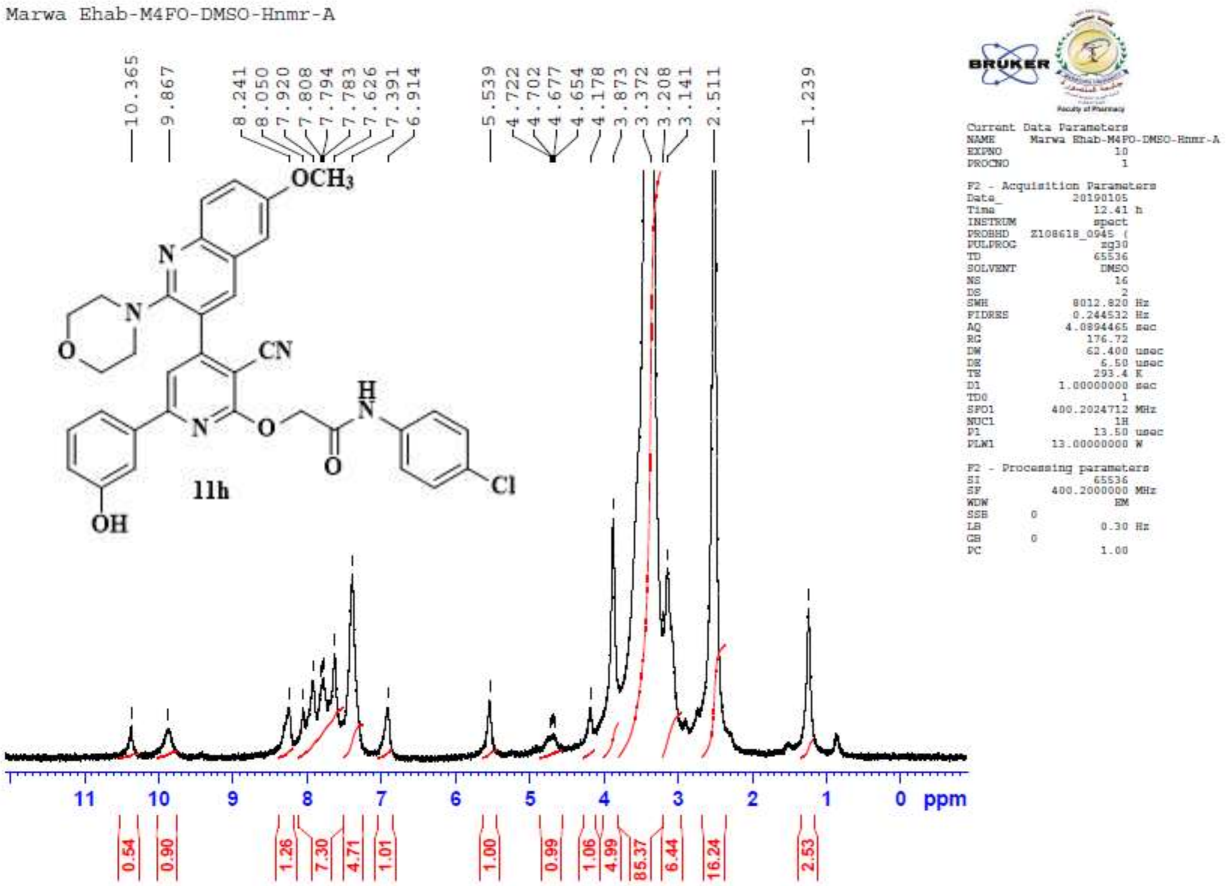

H[1]-NMR spectra of compound 11h

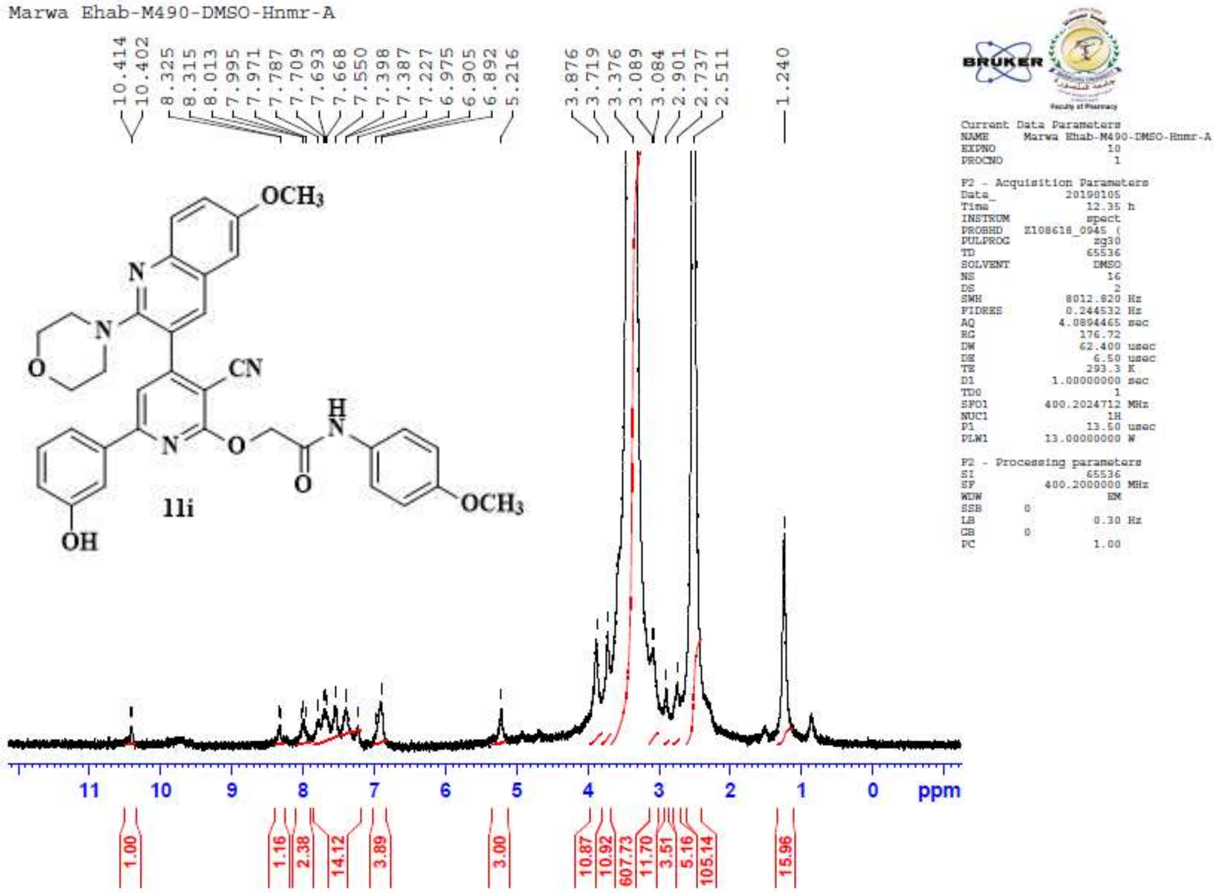

H[1]-NMR spectra of compound 11i

Information Classification: General

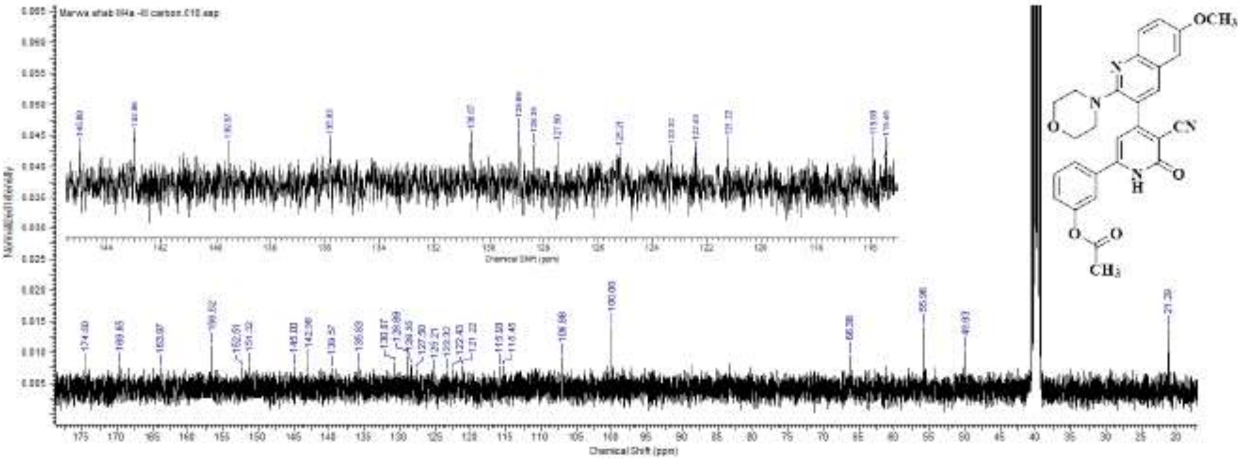

C[13]-NMR spectra of compound 7c

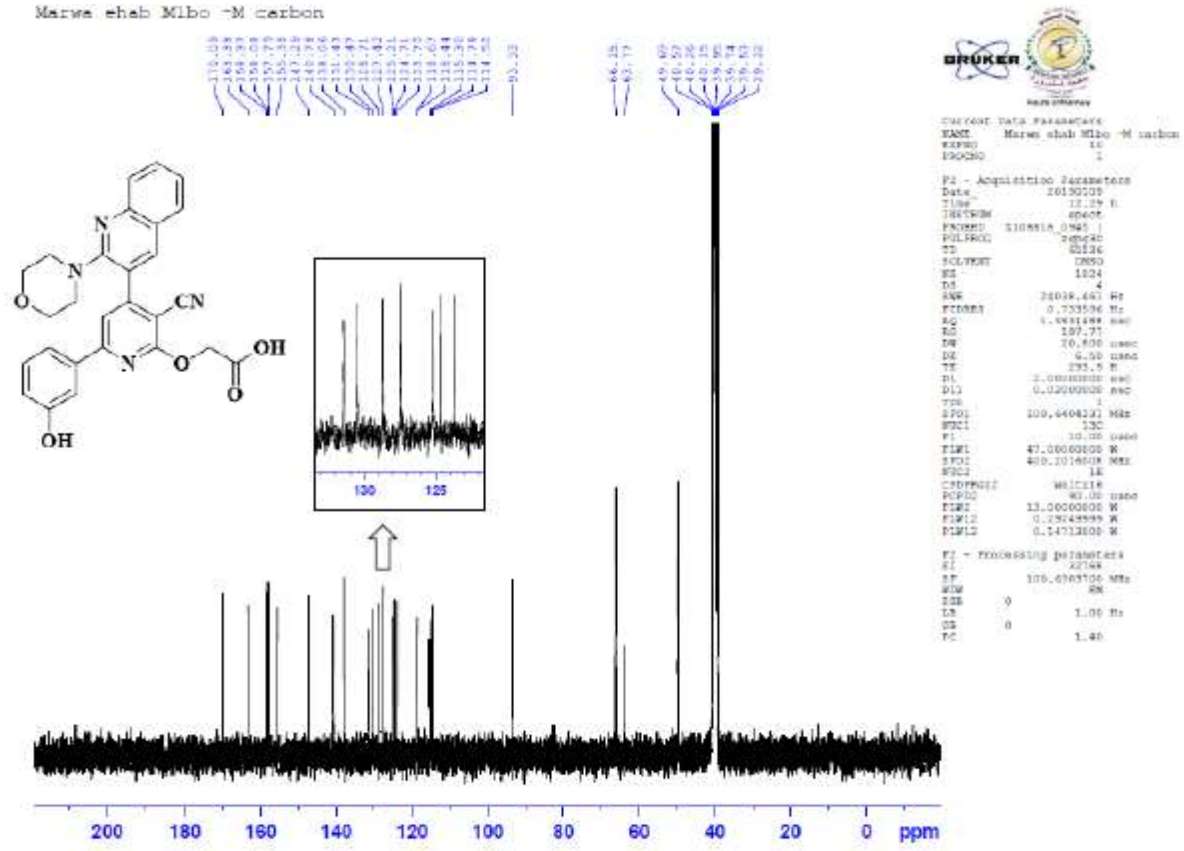

C[13]-NMR spectra of compound 9a

Information Classification: General

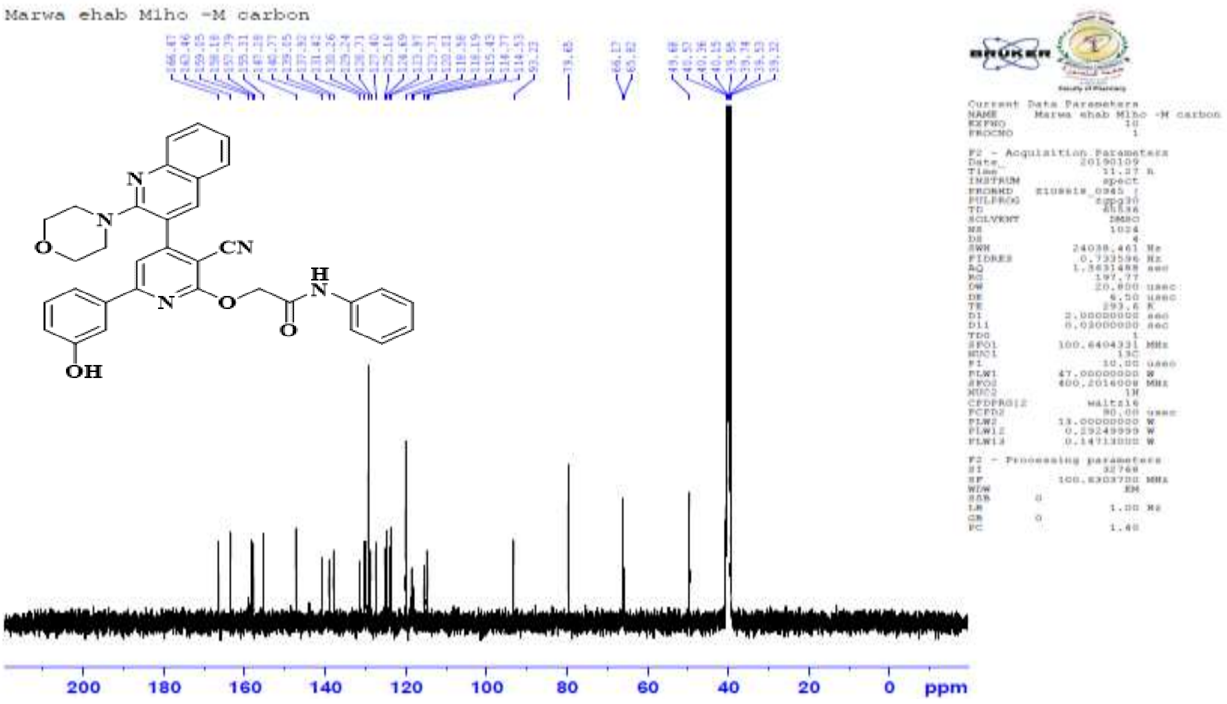

C13-NMR spectra of compound 11a

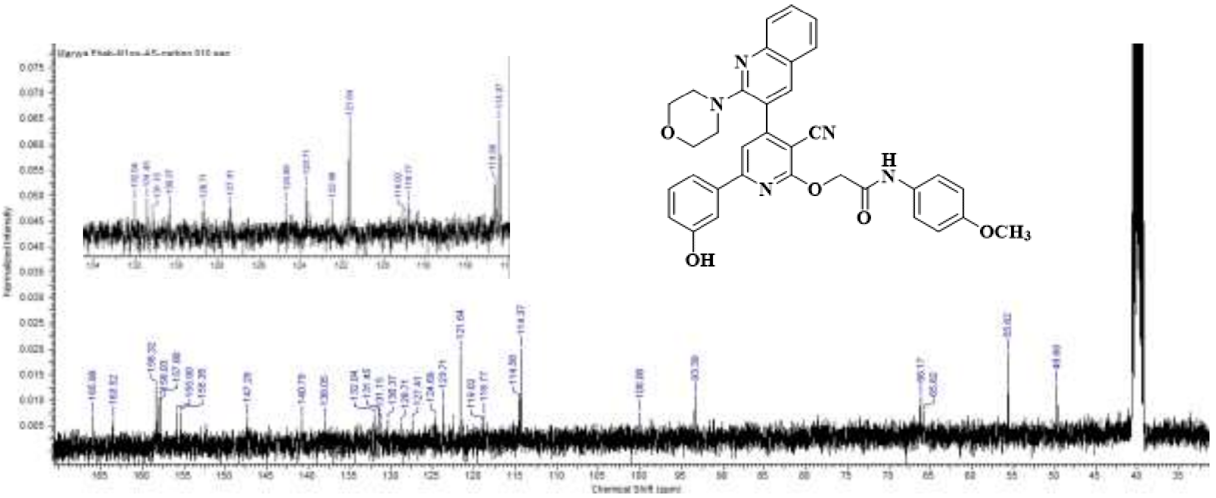

C13-NMR spectra of compound 11c

Information Classification: General

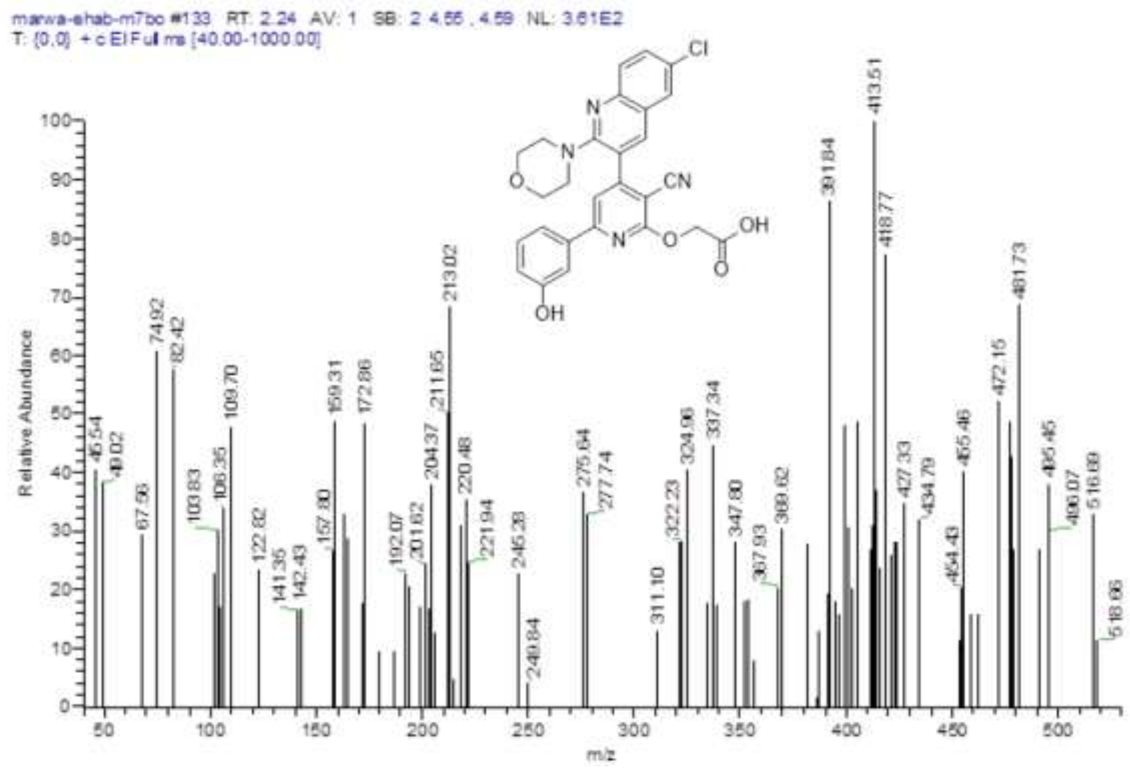

Mass spectrum for compound 9a

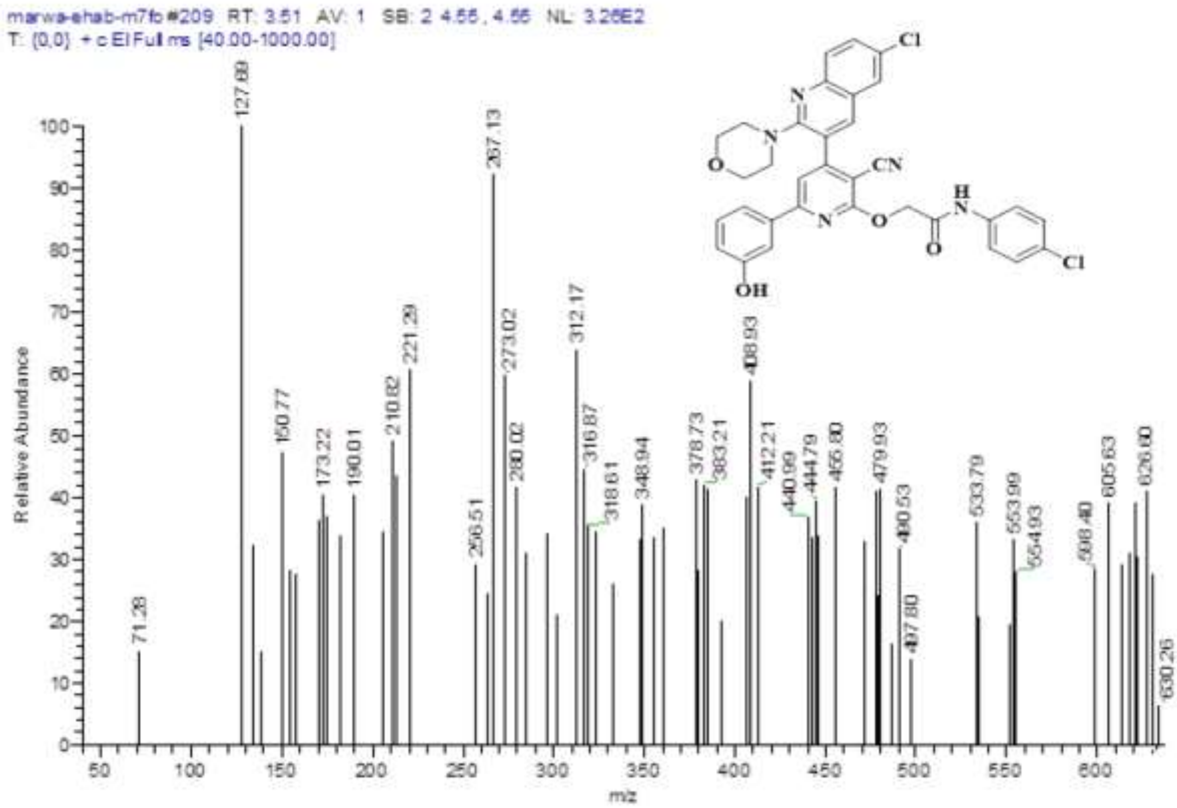

Mass spectrum for compound 11e

Information Classification: General

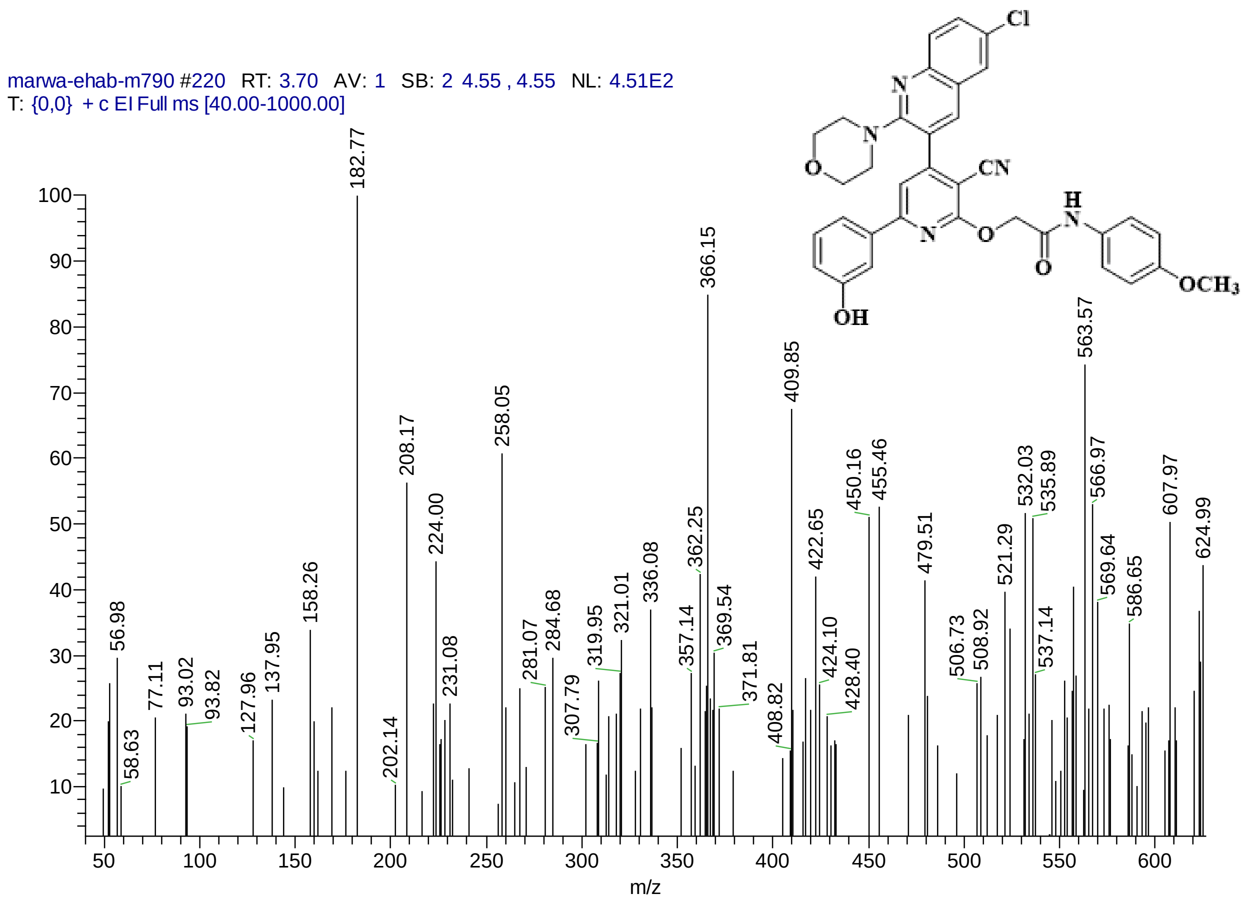

Mass spectrum for compound 11f

Information Classification: General

Al-Azhar University
The Regional Center for Mycology and Biotechnology

Requester Data:

Name: Dr. Marwa Ehab Mohamed
Authority: Faculty of Pharmacy, Alexandria University

Sample Data:

Twenty samples had been submitted for elemental analysis.

Analysis Report:

Sample Code	C%	H%	N%
M_1 eN	68.71	5.24	19.38
M_1 J	71.09	5.21	12.96
M_2	68.42	4.75	12.94
M_4 e	70.38	5.84	11.50
M_4 k	69.87	5.68	11.79
M_5	66.59	4.83	11.75
M_7 a **7b**	64.58	4.59	11.34
M_7 b **9b**	62.96	4.13	11.06
M_7 e	67.40	5.28	11.37
M_7 e N	63.26	4.69	17.58
M_7 f **11e**	63.44	4.21	11.39
M_7 h **11d**	66.81	4.62	11.94
M_7 J	66.26	4.69	11.72
M_7 k	66.49	4.88	11.69
M_8	63.45	4.11	11.94
N_2	71.47	5.28	13.02
N_5	69.43	5.34	12.13
O_1	71.49	4.06	15.95
O_4	68.95	4.32	14.67
O_7	64.71	3.19	14.62

INVESTIGATOR M. Elw

DIRECTOR

Information Classification: General

جامعة الأزهر
Al-Azhar University
المركز الإقليمى للفطريات وتطبيقاتها
The Regional Center for Mycology and Biotechnology

Requester Data:

Name: Dr. Marwa Ehab Abdel-Aziz
Authority: Faculty of Pharmacy,
Alexandria University

Sample Data:

Seven samples had been submitted for elemental analysis.

Analysis Report:

Sample Code	C%	H%	N%
M_1 go **11c**	69.32	5.13	12.18
M_1 ko	71.50	5.49	12.61
M_4 d4o	68.94	4.97	16.27
M_7 do	67.91	4.38	11.49
M_7 d2o	61.53	3.98	17.08
M_7 d3o	64.89	4.58	15.35
M_7 go **11f**	65.47	4.63	11.45

INVESTIGATOR **DIRECTOR**

Information Classification: General

[illegible]

Authority: Faculty of Pharmacy,
Alexandria University

Sample Data:

Twenty samples had been submitted for elemental analysis.

Analysis Report:

Sample Code	C%	H%	N%
M1 a 7a	69.30	4.94	12.29
M1 b 9a	67.49	4.83	11.88
M1 d	72.86	4.85	12.40
M1 e	71.88	5.78	12.23
M1 f 11b	67.13	4.60	12.06
M1 h 11a	70.89	4.96	12.31
M1 d_2	65.41	4.29	18.06
M1 d_3	68.98	4.57	16.24
M1 d_4	70.43	4.89	17.09
M4 a 7c	67.54	5.01	11.49
M4 b 9c	65.90	4.89	11.24
M4 d	70.51	5.13	11.57
M4 f 11h	65.92	4.67	11.52
M4 g 11i	68.34	5.24	11.49
M4 h 11g	69.26	4.86	11.23
M4 d_2	64.31	4.57	16.79
M4 d_3	67.52	5.04	15.45
M4 eN	66.54	5.37	17.83
O_3	68.43	4.65	12.99
O_6	66.59	4.83	12.17

INVESTIGATOR

DIRECTOR

Information Classification: General

Scheme 3:

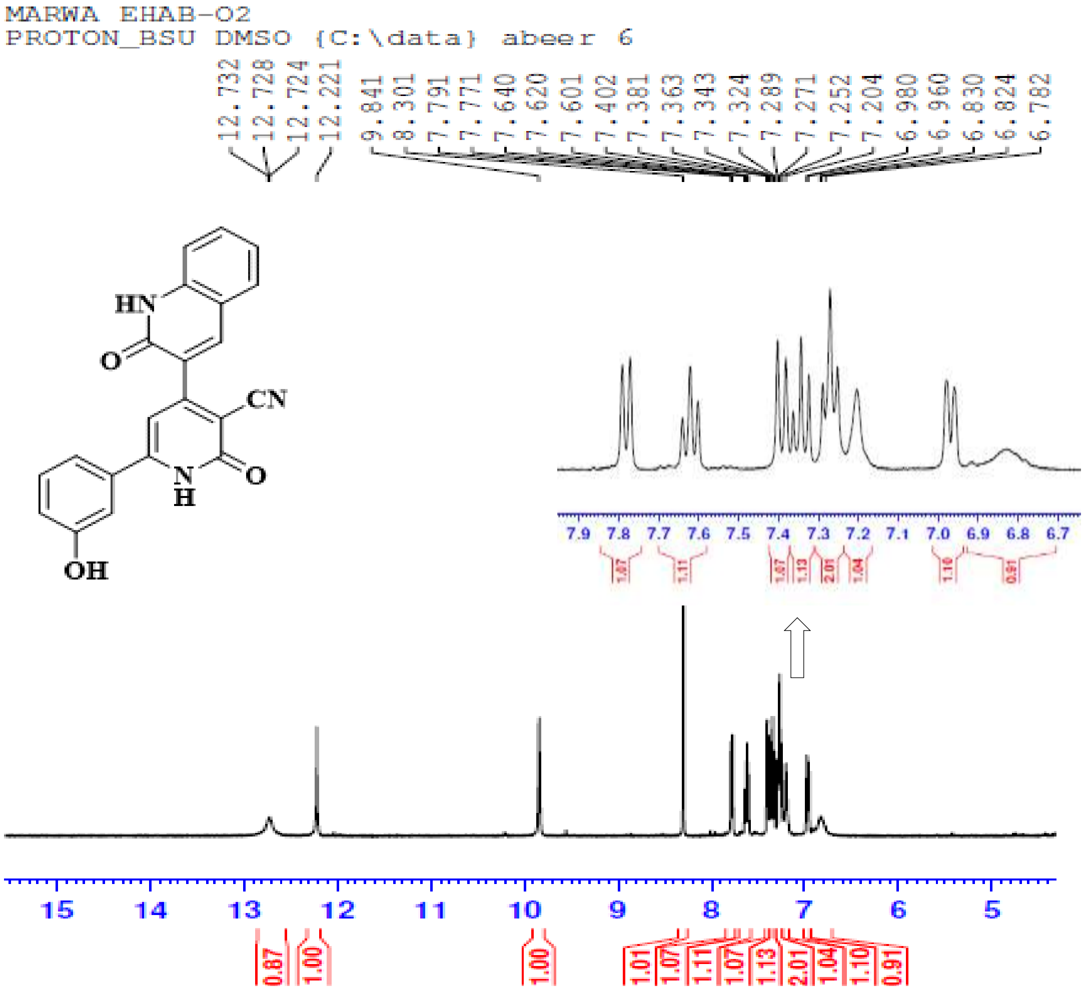

H¹-NMR spectra of compound 5a

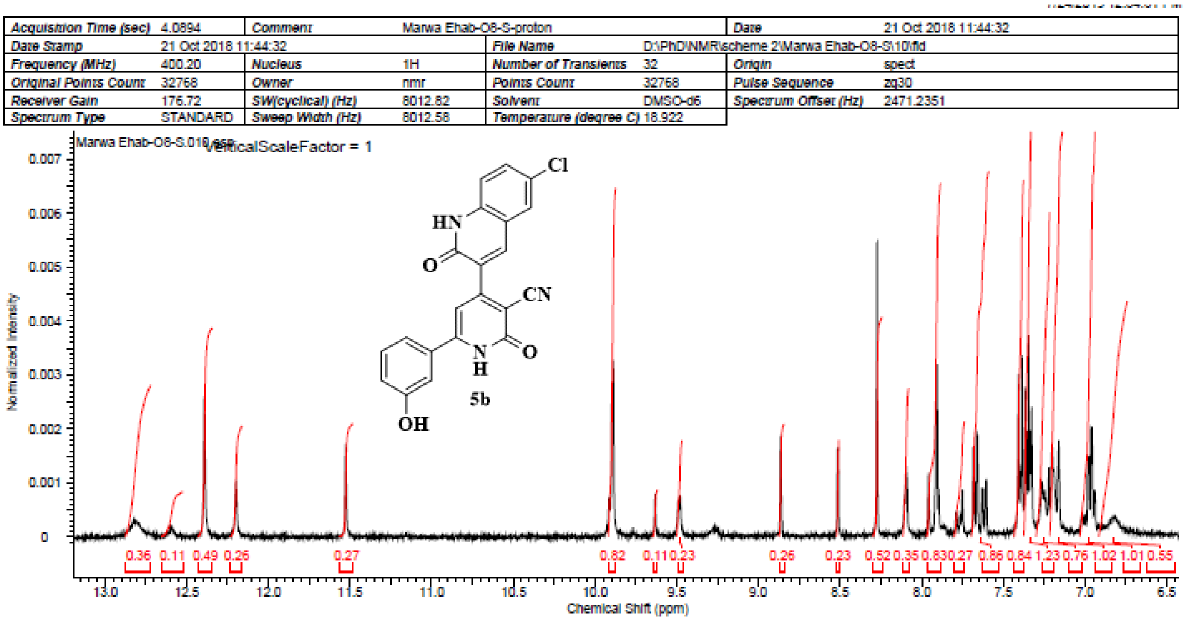

H¹-NMR spectra of compound 5b

Information Classification: General

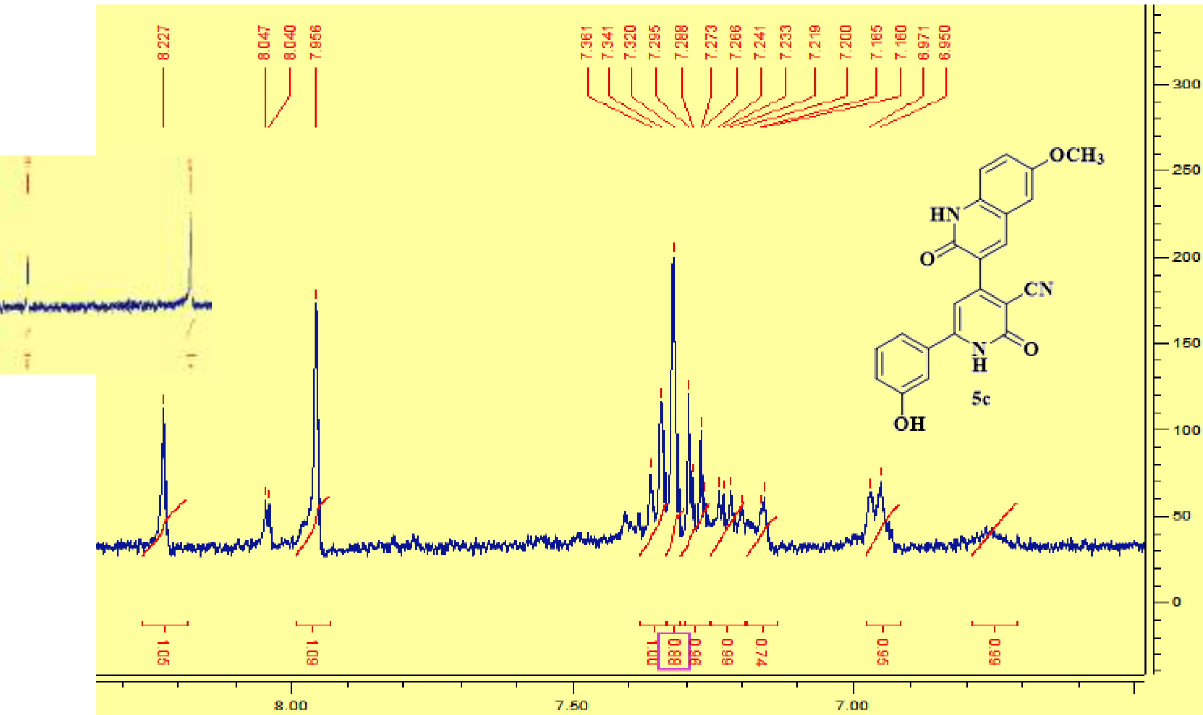

H[1]-NMR spectra of compound 5c

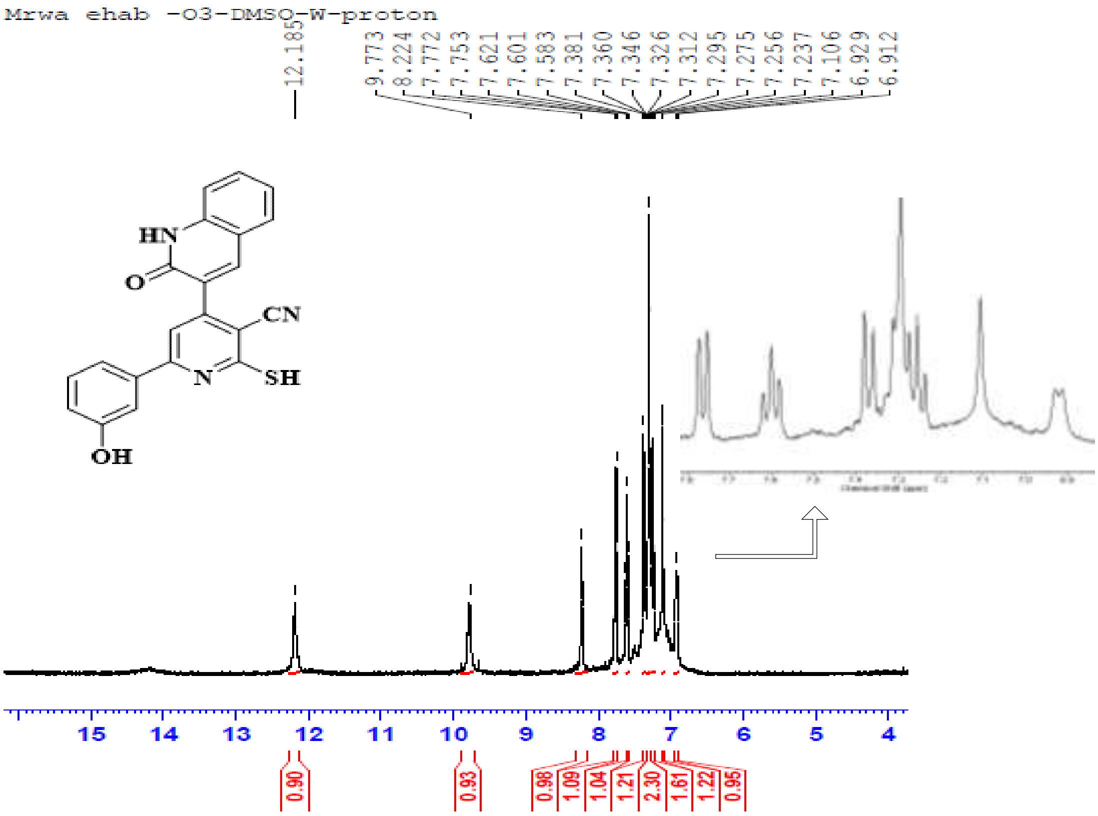

H[1]-NMR spectra of compound 6a

Information Classification: General

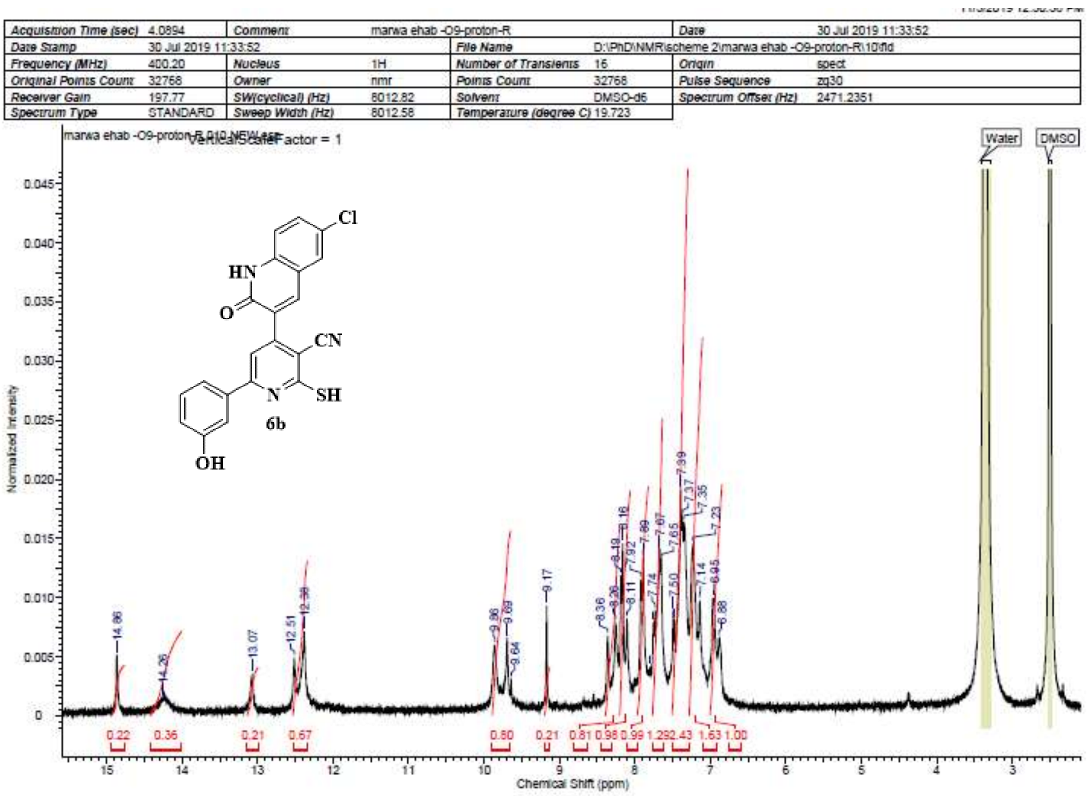

H[1]-NMR spectra of compound 6b

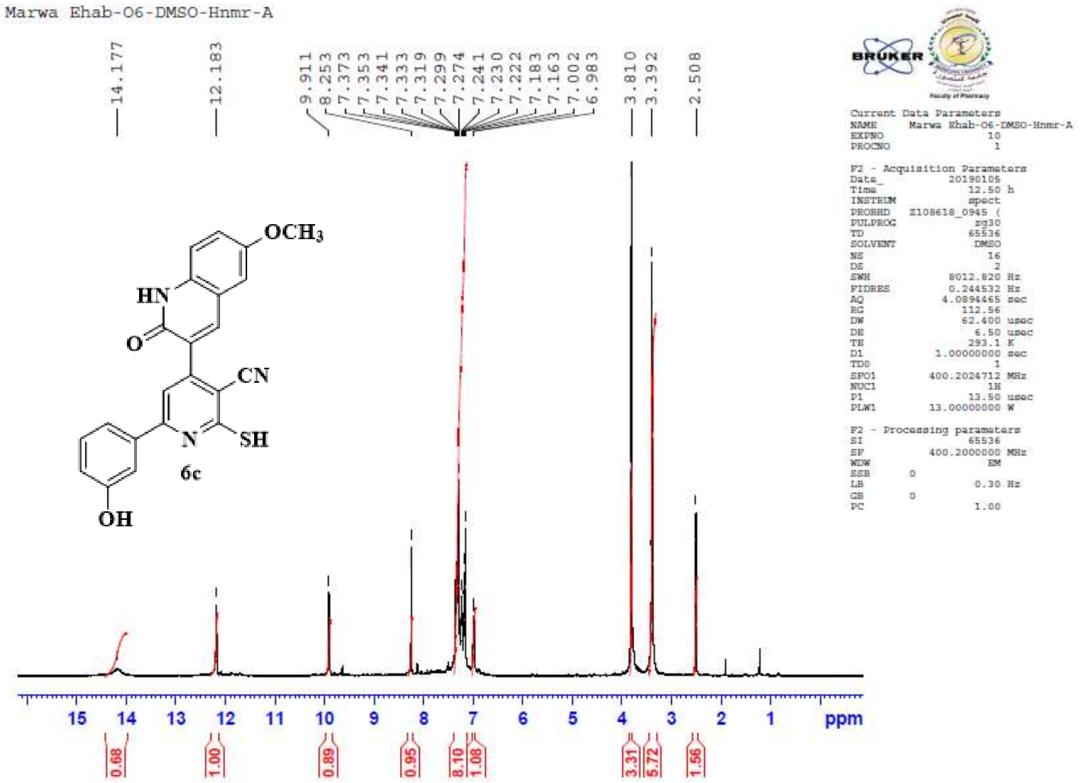

H[1]-NMR spectra of compound 6c

Information Classification: General

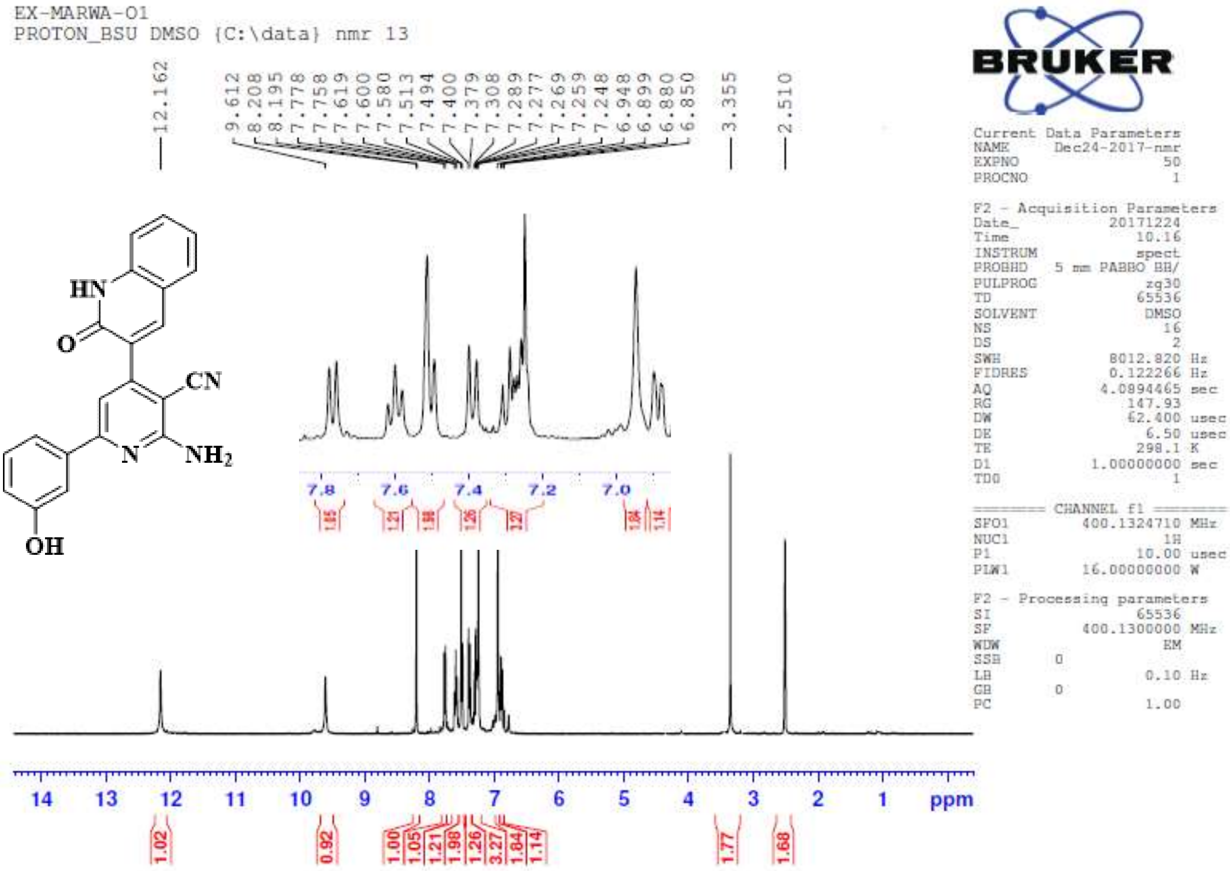

H¹-NMR spectra of compound 7a

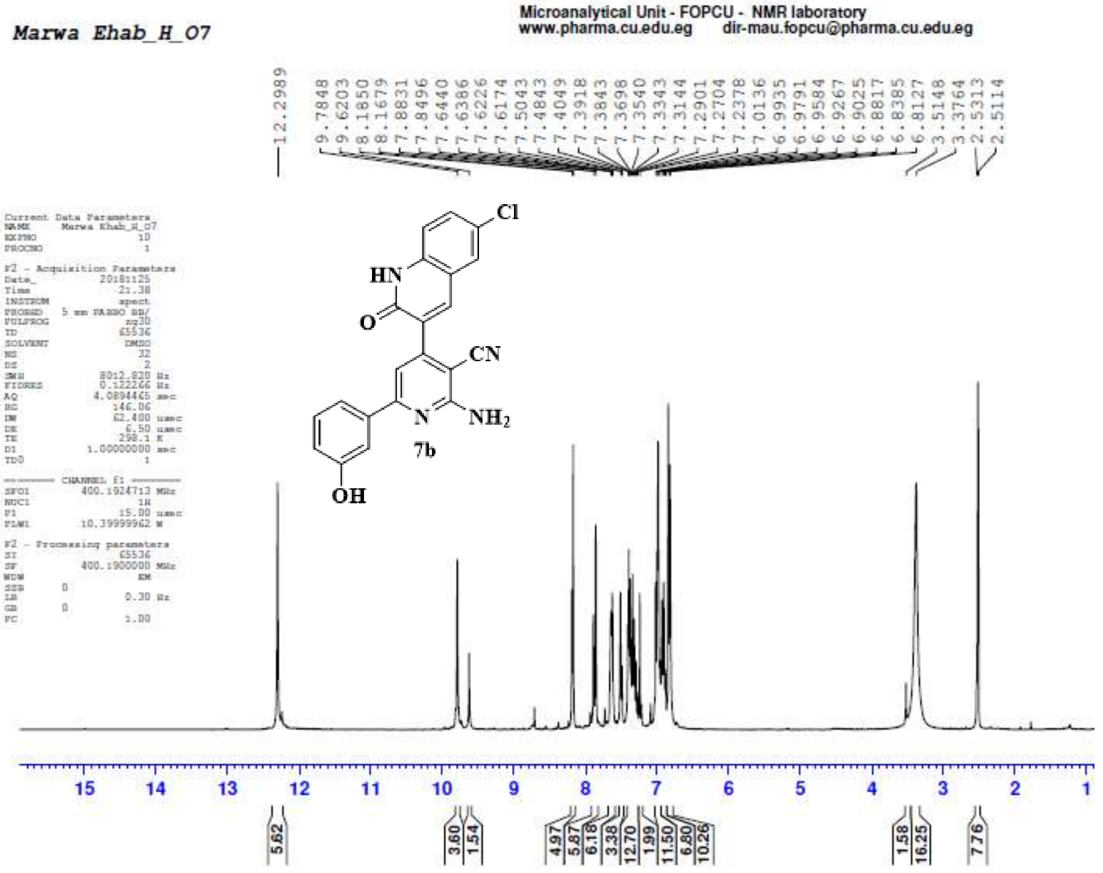

H¹-NMR spectra of compound 7b

Information Classification: General

This report was created by ACD/NMR Processor Academic Edition. For more information go to www.acdlabs.com/nmrproc/

7/24/2019 1:26:43 PM

Acquisition Time (sec)	4.0894	Comment	EX-MARWAEHAB-O4 PROTON BSU DMSO {C:\data} abeer 11				
Date	14 Aug 2018 10:27:44			Date Stamp	14 Aug 2018 10:27:44		
File Name	D:\PhD\NMR\scheme 2\O4\fid			Frequency (MHz)	400.13	Nucleus	1H
Number of Transients	16	Origin	spect	Original Points Count	32768	Owner	nmr
Points Count	32768	Pulse Sequence	zg30	Receiver Gain	147.93	SW(cyclical) (Hz)	8012.82
Solvent	DMSO-d6	Spectrum Offset (Hz)	2470.9668	Spectrum Type	STANDARD	Sweep Width (Hz)	8012.58
Temperature (degree C)	25.141						

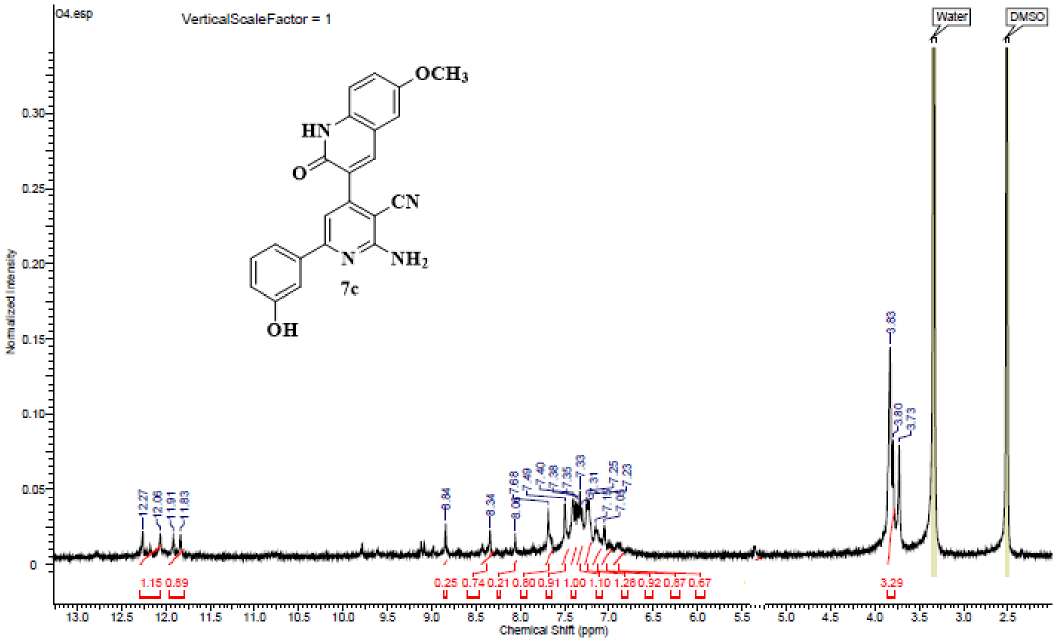

H^1-NMR spectra of compound 7c

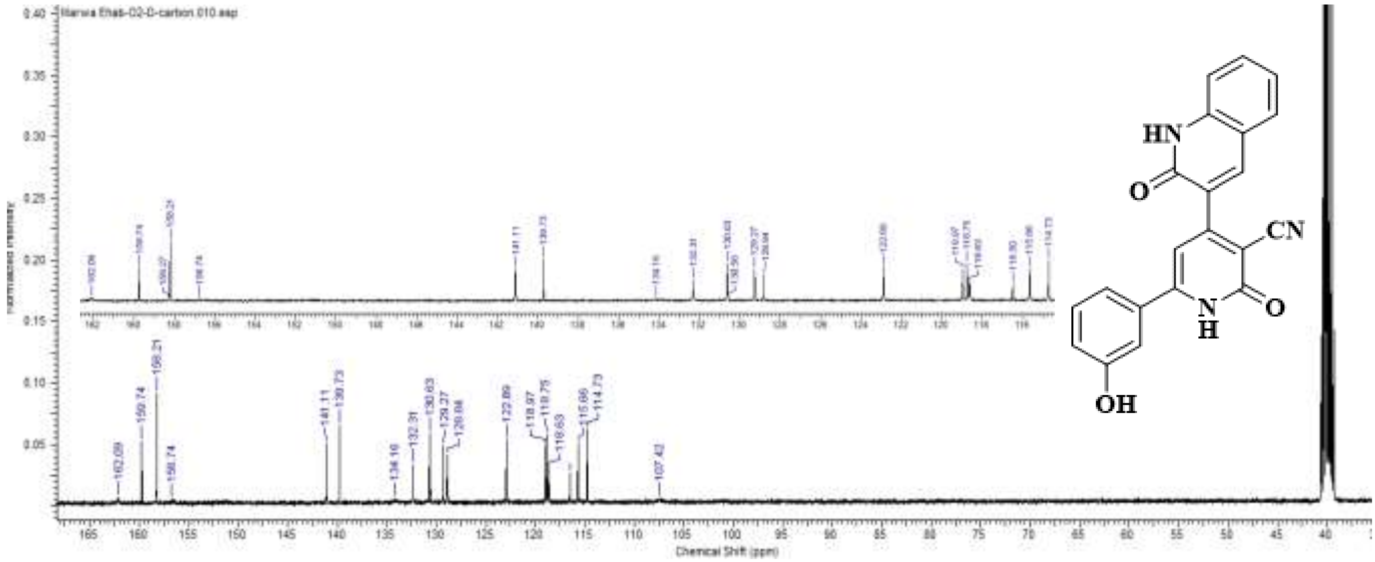

C^{13}-NMR spectra of compound 5a

Information Classification: General

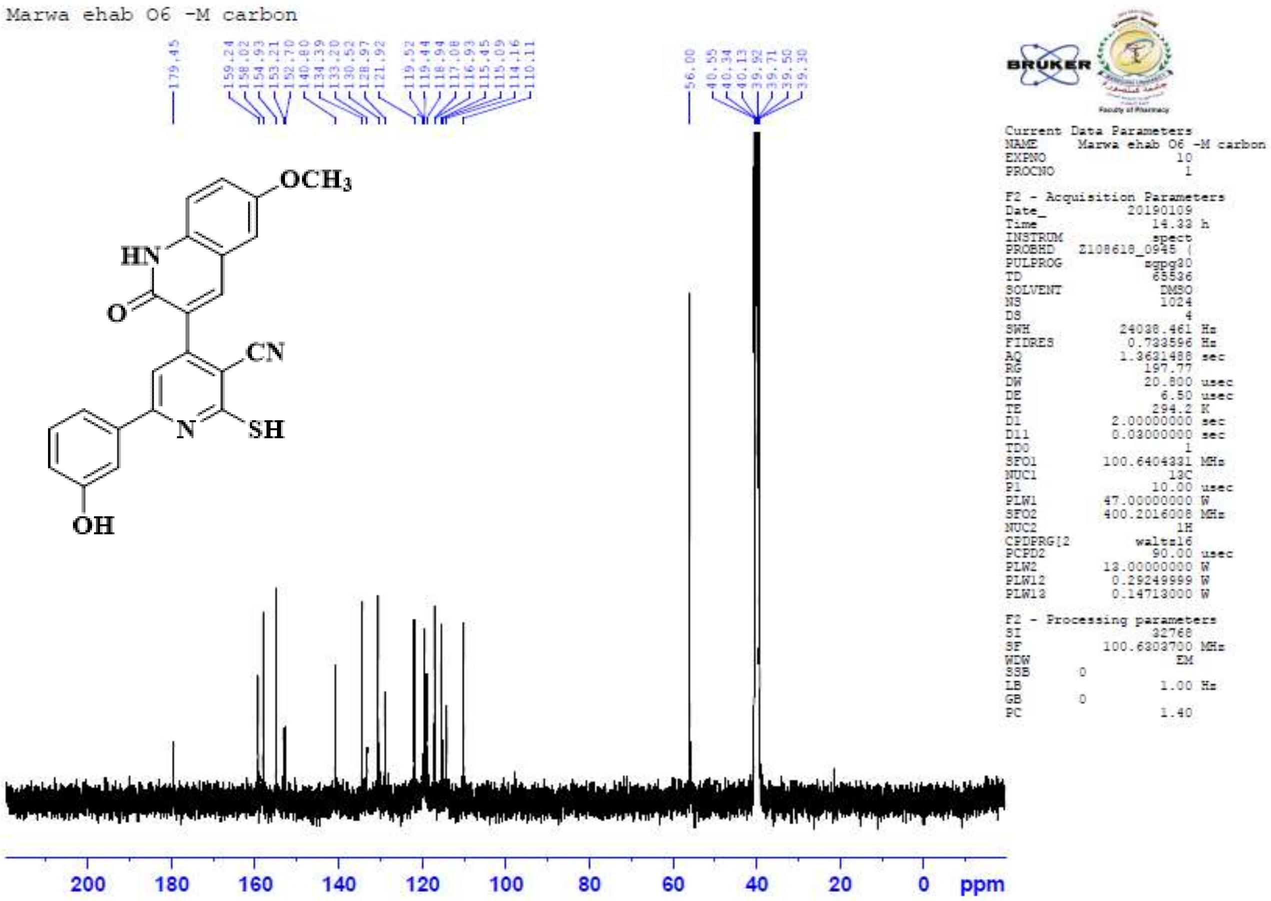

C^{13}-NMR spectra of compound 6c

Information Classification: General

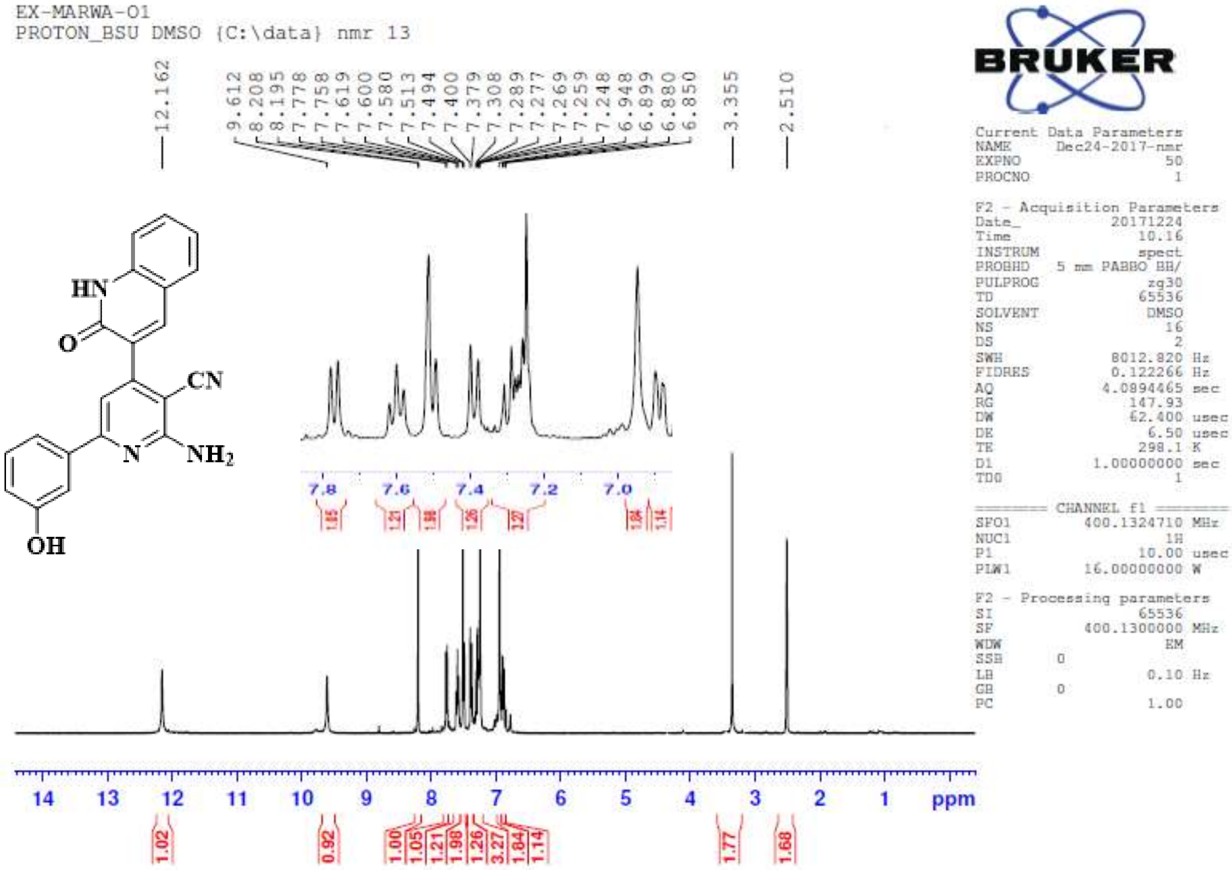

C^{13}-NMR spectra of compound 7a

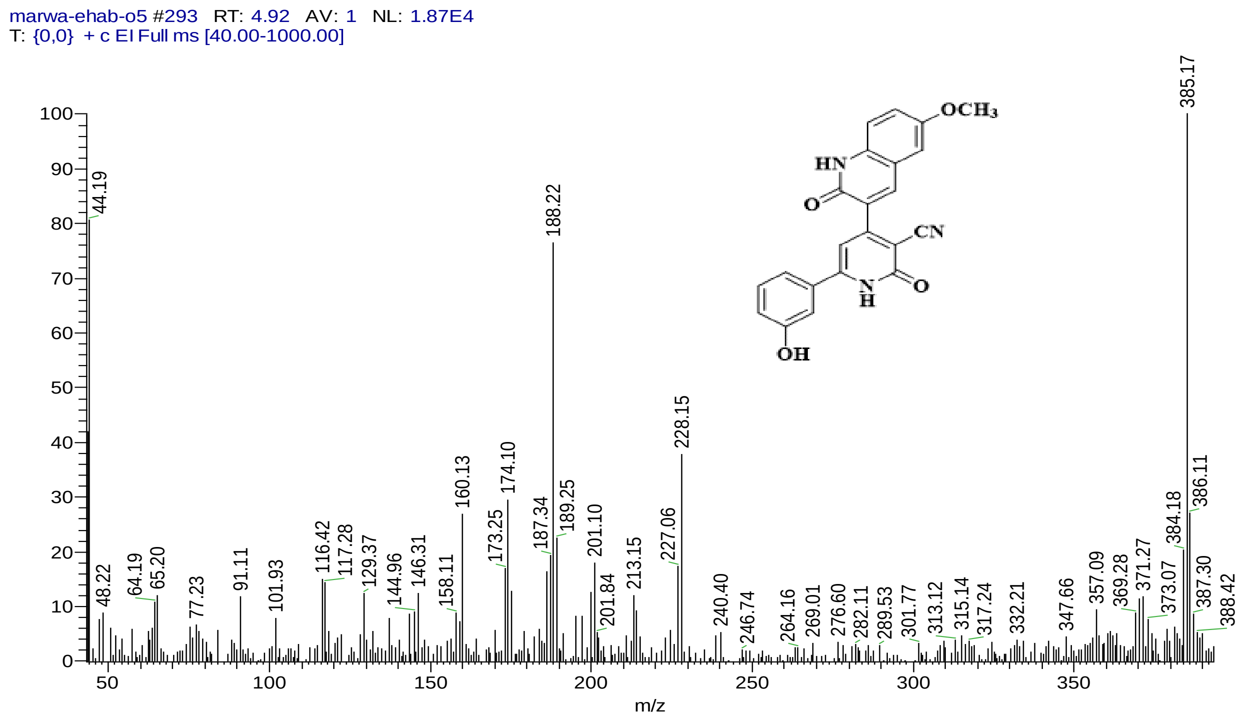

EI/MS spectrum of compound 5c

Information Classification: General

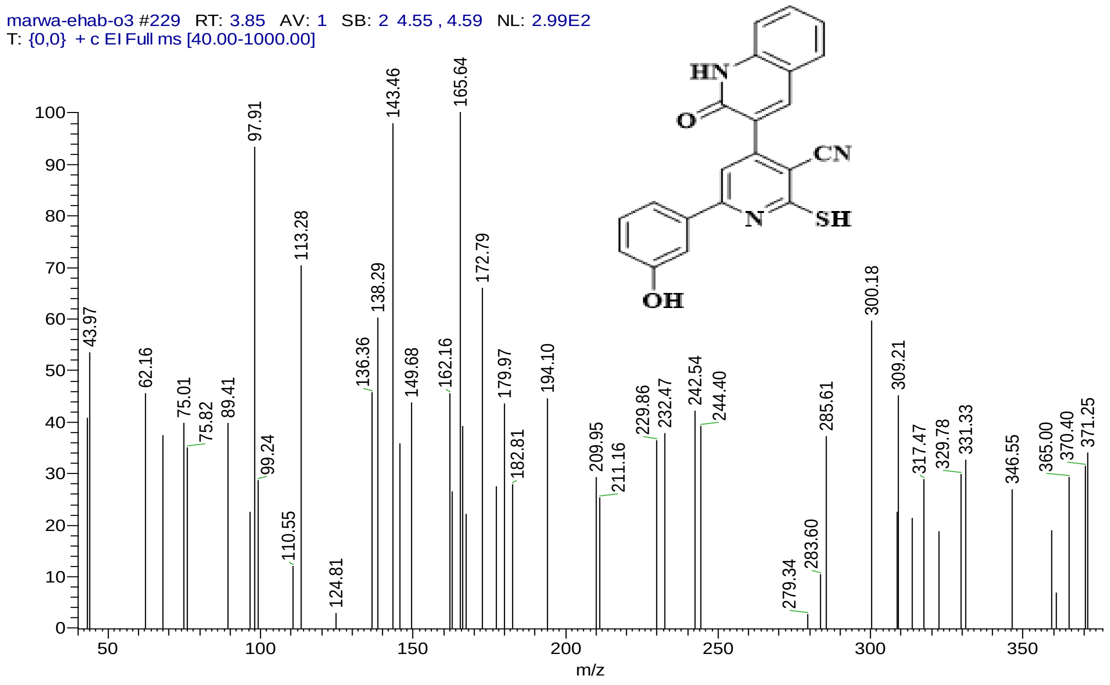

EI/MS spectrum of compound 6a

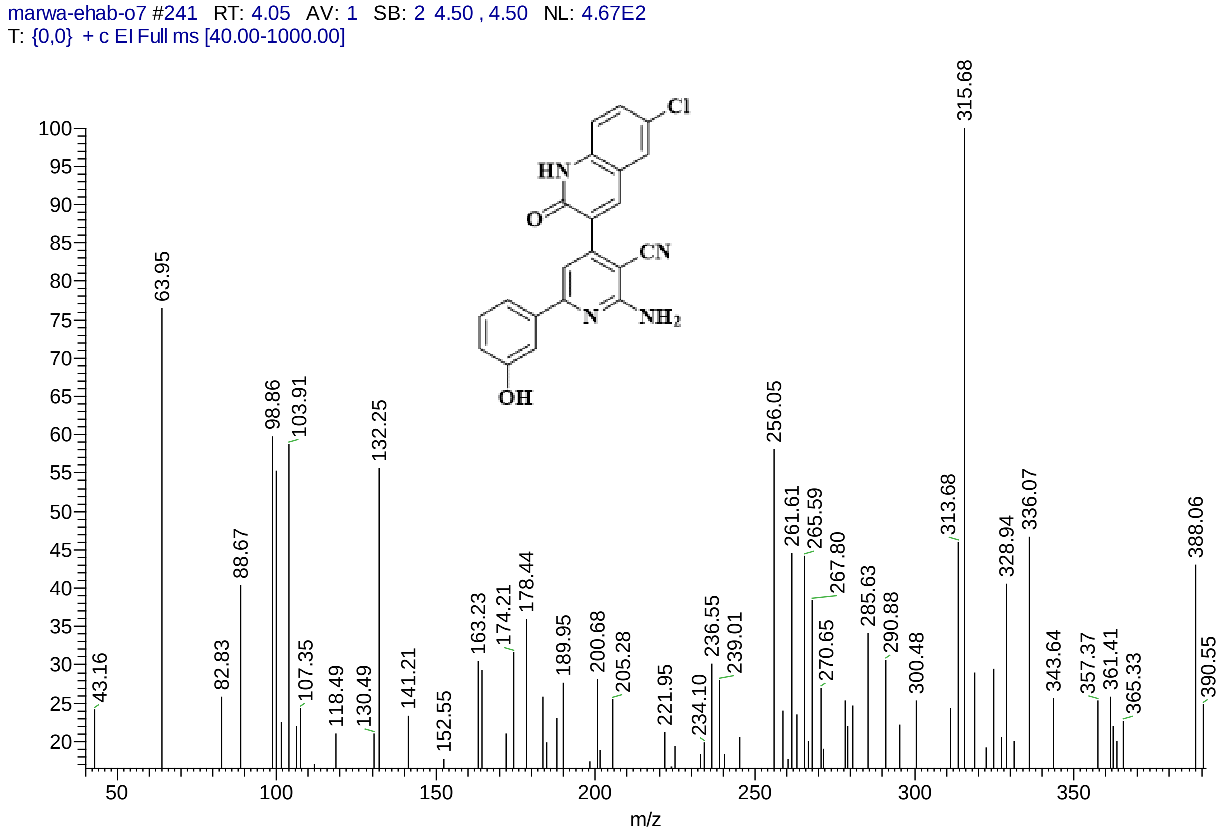

EI/MS spectrum of compound 7b

Information Classification: General

Al-Azhar University
The Regional Center for Mycology and Biotechnology

Requester Data:

Name: Dr. Marwa Ehab Mohamed
Authority: Faculty of Pharmacy,
Alexandria University

Sample Data:

Eleven samples had been submitted for elemental analysis.

Analysis Report:

Sample Code	C%	H%	N%
CH_3	68.01	3.43	7.79
CH_5	68.56	3.88	7.42
M_1	71.02	4.89	13.43
M_4	68.94	5.12	12.59
M_7	65.27	4.28	12.47
N_1	74.19	5.39	13.53
N_4	71.90	5.26	12.56
N_7	68.60	4.80	12.49
O_2 **5a**	70.69	3.84	11.97
O_5 **5c**	68.80	3.85	10.79
O_8 **5b**	64.89	3.32	10.85

INVESTIGATOR M. Elwa

DIRECTOR G. Sheh

Information Classification: General

Al-Azhar University

The Regional Center for Mycology and Biotechnology

Requester Data:

Name: Dr. Marwa Ehab Mohamed

Authority: Faculty of Pharmacy, Alexandria University

Sample Data:

Twenty samples had been submitted for elemental analysis.

Analysis Report:

Sample Code	C%	H%	N%
M_1 eN	68.71	5.24	19.38
M_1 J	71.09	5.21	12.96
M_2	68.42	4.75	12.94
M_4 e	70.38	5.84	11.50
M_4 k	69.87	5.68	11.79
M_5	66.59	4.83	11.75
M_7 a	64.58	4.59	11.34
M_7 b	62.96	4.13	11.06
M_7 e	67.40	5.28	11.37
M_7 e N	63.26	4.69	17.58
M_7 f	63.44	4.21	11.39
M_7 h	66.81	4.62	11.94
M_7 J	66.26	4.69	11.72
M_7 k	66.49	4.88	11.69
M_8	63.45	4.11	11.94
N_2	71.47	5.28	13.02
N_5	69.43	5.34	12.13
O_1 **7a**	71.49	4.06	15.95
O_4 **7c**	68.95	4.32	14.67
O_7 **7b**	64.71	3.19	14.62

INVESTIGATOR M. Ehab

DIRECTOR

Information Classification: General

جامعة الأزهر
Al-Azhar University
المركز الإقليمى للفطريات وتطبيقاتها
The Regional Center for Mycology and Biotechnology

Requester Data:

Name: Dr. Marwa Ehab Abdel-Aziz
Authority: Faculty of Pharmacy,
Alexandria University

Sample Data:

Nine samples had been submitted for elemental analysis.

Analysis Report:

Sample Code	C%	H%	N%
M_3	73.68	5.12	13.89
M_3 r	70.69	5.23	16.75
M_6	71.43	5.24	12.97
N_3	76.71	5.70	13.96
N_6	74.52	5.68	12.59
N_8	65.89	4.65	11.76
O_3 **6a**	67.69	3.75	11.48
O_6 **6c**	65.70	3.98	10.60
O_9 **6b**	62.41	3.16	10.44

INVESTIGATOR **DIRECTOR**

Information Classification: General

Table 1: Anticancer activities IC_{50} (µM) of the tested compounds against human cancer cells, In vitro cytotoxicity and selectivity index (SI) values of the tested compounds.

Cpd No	Wi-38		HepG-2		Caco-2		PC-3		NFS-60	
	EC_{50}(µM)	EC_{100}(µM)	IC_{50}(µM)	SI	IC_{50}(µM)	SI	IC_{50}(µM)	SI	IC_{50}(µM)	SI
**5a**	0.0603±0.04	0.0192±0.003	0.0344±0.006	1.753	0.0388±0.008	1.554	0.0716±0.0.4	0.842	0.03162±0.0001	1.907
**5b**	0.171±0.002	0.048±0.0001	**0.00528±0.0001**	32.386	**0.0453±0.001**	3.775	**0.0261±0.004**	6.552	**0.00932±0.004**	18.348
**5c**	0.717±0.002	0.361±0.014	**0.0076±0.0001**	94.342	**0.0361±0.0001**	19.861	**0.0272±0.003**	26.360	**0.0181±0.001**	39.613
**5d**	0.0299±0.0001	0.0239±0.0003	0.0897±0.013	0.333	0.0386±0.001	0.775	0.05341±0.004	0.560	0.07126±0.003	0.420
**5e**	0.0276±0.001	0.0234±0.001	0.03612±0.001	0.764	0.0517±0.005	0.534	0.0409±0.001	0.675	0.06267±0.027	0.440
**5f**	0.0298±0.002	0.0258±0.008	0.0378±0.01	0.788	0.0508±0.004	0.587	0.0451±0.0001	0.661	0.04283±0.0003	0.696
**6a**	0.181±0.005	0.0785±0.003	0.074±0.002	2.446	0.104±0.004	1.740	0.127±0.002	1.425	0.135±0.002	1.341
**6b**	0.138±0.002	0.0493±0.001	0.0466±0.001	2.961	0.0552±0.003	2.500	0.119±0.014	1.160	0.204±0.006	0.676
**6c**	0.376±0.001	0.177±0.011	0.0848±0.002	4.434	0.0999±0.003	3.764	0.1008±0.0001	3.730	0.145±0.015	2.593
**6d**	0.759±0.025	0.212±0.009	0.0876±0.002	8.664	0.0708±0.003	10.720	0.135±0.011	5.622	0.217±0.022	3.498
**6e**	0.155±0.003	0.0277±0.0001	**0.0122±0.0001**	12.705	**0.027±0.002**	5.741	**0.0429±0.003**	3.613	**0.0539±0.0001**	2.876
**7a**	0.174±0.005	0.102±0.01	0.0495±0.0001	3.515	0.0799±0.003	2.178	0.140±0.02	1.243	0.145±0.002	1.200
**7b**	0.140±0.009	0.110±0.002	0.1016±0.001	1.378	0.119±0.016	1.176	0.135±0.002	1.037	0.107±0.001	1.308
**7c**	0.137±0.0001	0.094±0.005	0.1421±0.001	0.964	0.127±0.014	1.079	0.101±0.001	1.356	0.153±0.034	0.895
**9a**	0.159±0.004	0.0254±0.002	0.0782±0.002	2.033	0.0739±0.0001	2.152	0.0847±0.003	1.877	0.0923±0.0001	1.723
**9b**	0.171±0.001	0.134±0.01	0.0454±0.003	3.767	0.0552±0.002	3.098	0.0973±0.008	1.757	0.380±0.01	0.450
**9c**	0.276±0.002	0.0754±0.003	0.0867±0.003	3.183	0.0526±0.0001	5.247	0.0462±0.002	5.974	0.0961±0.002	2.872

Information Classification: General

**Cpd No**	**Wi-38**		**HepG-2**		**Caco-2**		**PC-3**		**NFS-60**	
	**EC_{50}(µM)**	**EC_{100}(µM)**	**IC_{50}(µM)**	**SI**	**IC_{50}(µM)**	**SI**	**IC_{50}(µM)**	**SI**	**IC_{50}(µM)**	**SI**
**11a**	0.144±0.001	0.0906±0.012	0.336±0.022	0.429	0.158±0.002	0.911	0.158±0.026	0.911	0.126±0.006	1.143
**11b**	0.337±0.024	0.00232±0.001	0.0715±0.001	4.713	0.063±0.002	5.349	0.130±0.011	2.592	0.181±0.007	1.862
**11c**	0.101±0.001	0.0483±0.004	0.281±0.007	0.359	0.100±0.014	1.010	0.159±0.007	0.635	0.110±0.002	0.918
**11d**	0.107±0.001	0.0455±0.002	0.0458±0.001	2.336	0.0521±0.002	2.054	0.0594±0.001	1.801	0.0927±0.0001	1.154
**11e**	0.085±0.003	0.0178±0.0001	0.0416±0.004	2.043	0.0311±0.006	2.733	0.0444±0.004	1.914	0.0532±0.002	1.598
**11f**	0.181±0.013	0.0347±0.003	0.0363±0.001	4.986	0.0344±0.003	5.262	0.0476±0.0001	3.803	0.0998±0.011	1.814
**11g**	0.186±0.01	0.0450±0.005	0.0628±0.001	2.962	0.0626±0.001	2.971	0.0839±0.003	2.217	0.103±0.009	1.806
**11h**	0.166±0.003	0.0182±0.001	0.074±0.002	2.243	0.0829±0.003	2.002	0.0709±0.0001	2.341	0.0781±0.007	2.125
**11i**	0.258±0.031	0.148±0.0001	0.0486±0.001	5.309	0.0347±0.003	7.435	0.0634±0.002	4.069	0.523±0.04	0.493
**13a**	0.0255±0.004	0.0145±0.0001	**0.00238±0.0001**	10.714	**0.0228±0.001**	1.118	**0.0200±0.003**	1.275	**0.009±0.0001**	2.833
**13b**	0.0486±0.016	0.017±0.002	0.0309±0.001	1.573	0.04836±0.005	1.005	0.0388±0.003	1.253	0.03373±0.002	1.441
**13c**	0.0348±0.003	0.0242±0.001	**0.00377±0.0001**	9.231	**0.0337±0.006**	1.033	**0.0225±0.002**	1.547	**0.0088±0.0001**	3.955
**14a**	0.299±0.004	0.134±0.003	**0.0127±0.003**	23.543	**0.0141±0.004**	21.206	**0.0168±0.0001**	17.798	**0.0232±0.002**	12.888
**14b**	0.196±0.012	0.153±0.005	0.125±0.002	1.568	0.146±0.019	1.342	0.140±0.004	1.400	0.135±0.003	1.452
**14c**	0.166±0.009	0.0972±0.005	0.0557±0.003	2.980	0.0842±0.005	1.971	0.0626±0.0001	2.652	0.0965±0.004	1.720
**15a**	0.202±0.003	0.0663±0.003	0.108±0.007	1.870	0.191±0.019	1.058	0.242±0.037	0.835	0.247±0.016	0.818
**15b**	0.264±0.006	0.169±0.009	0.299±0.008	0.883	0.194±0.002	1.361	0.196±0.061	1.347	0.323±0.007	0.817
**15c**	0.121±0.002	0.101±0.005	0.107±0.011	1.131	0.141±0.021	0.858	0.143±0.013	0.846	0.122±0.001	0.992
**11a**	0.144±0.001	0.0906±0.012	0.336±0.022	0.429	0.158±0.002	0.911	0.158±0.026	0.911	0.126±0.006	1.143
**11b**	0.337±0.024	0.00232±0.001	0.0715±0.001	4.713	0.063±0.002	5.349	0.130±0.011	2.592	0.181±0.007	1.862
**11c**	0.101±0.001	0.0483±0.004	0.281±0.007	0.359	0.100±0.014	1.010	0.159±0.007	0.635	0.110±0.002	0.918
**11d**	0.107±0.001	0.0455±0.002	0.0458±0.001	2.336	0.0521±0.002	2.054	0.0594±0.001	1.801	0.0927±0.0001	1.154

Information Classification: General

Cytotoxicity screening

Cytotoxicity of the tested compounds was evaluated using normal human lung fibroblast Wi-38 cell line compared to currently used anticancer drug (doxorubicin). Firstly, Wi-38 cell line was subcultured in DMEM medium-contained 10% fetal bovine serum (FBS), then, seeded as $5x10^3$ cells per well in 96-well cell culture plate and incubated at 37ºC in 5% CO_2 incubator. Serial concentrations of these compounds and doxorubicin (Dox) were incubated with Wi-38 cells for 72 h after 24 h for cell attachment. MTT method was used for assaying cell viability[25]. Twenty microliters of 5 mg/ml MTT (Sigma, USA) was added to each well and the plate was incubated at 37ºC for 3 h. Then MTT solution was removed, 100 µl DMSO was used and the absorbance of each well was measured with a microplate reader (BMG LabTech, Germany) at 570 nm. The effective concentration (IC_{50}) and safe dose (EC_{100}) values of the tested compounds that cause 50% and 100% cell viability were estimated by the Graphpad Instat software.

Determination of the anticancer activity

Anticancer effect of the above-mentioned compounds was evaluated using four human cancer cell lines. Colon cancer cells (Caco-2) were maintained in DMEM (Lonza, USA) containing 10% FBS while myeloid leukemia cell line (NFS-60), liver cancer cell line (HepG-2) and prostate cancer cell line (PC-3) were cultured in RPMI-1640 (Lonza, USA) supplemented with 10% FBS. All cancer cells ($5x10^3$ cells/well) were seeded in sterile 96-well plates. After 24h, serial concentrations of the tested compounds and Dox were incubated with four cancer cell lines for 72 h at 37ºC in 5% CO_2 incubator. MTT method was done as described above. The Graphpad Instat software was used for calculating the half maximal inhibitory concentration (IC_{50}) values. Furthermore, phase contrast inverted microscope with a digital camera (Olympus, Japan) was used for investigating cellular morphological changes before and after treatment with the most effective and safest anticancer compounds.

Flow cytometric analysis of apoptosis

The IC_{50} of the most effective compounds and Dox was incubated for 72 h with HepG2, Caco-2, PC-3 and NFS-60 cell lines. After trypsinization, the untreated and treated cells were incubated with annexin V/PI for 15 min. Then cells were fixed and incubated with streptavidin-fluorescein (5 µg/mL) for 15 min. The apoptosis-dependent anticancer effect was determined by quantification of annexin-stained apoptotic cells using the FITC signal detector (FL1) against the phycoerythrin emission signal detector (FL2).

Information Classification: General

Caspase 3/7 activation assay

The Apo-ONE ® caspase 3/7 kit following the manufacturer's instructions was used for quantifying the percentage of caspase 3/7 activation. The fluorescence substrate present in the kit was cut by caspases to produce fluorescence signal. This signal was measured by the fluorescence omega microplate reader (BMG LabTech, Germany) at 490 nm excitation and 520 nm emission.

Statistical Analysis

Data were expressed as mean ± standard error of the mean (SEM). Statistical significance was estimated by the multiple comparisons Tukey post-hoc analysis of variance (ANOVA) using the SPSS16 program. When $p < 0.05$, the differences were marked as statistically significant.

PIM-1 and PIM-2 kinase inhibitory activity

The most active anticancer compounds were tested for their ability to *in-vitro* inhibit PIM-1 and PIM-2 kinase utilizing PIM-1 and PIM-2 Kinase Assay Kit – Promega Corporation catalog #V4032, following the manufacturer's instructions [26]

Information Classification: General
